# Association of vitamin D status with arterial blood pressure and hypertension risk: a mendelian randomisation study

**DOI:** 10.1016/S2213-8587(14)70113-5

**Published:** 2014-06-25

**Authors:** Karani S Vimaleswaran, Alana Cavadino, Diane J Berry, LifeLines Cohort Study investigators, Rolf Jorde, Aida Karina Dieffenbach, Chen Lu, Alexessander Couto Alves, Hiddo J Lambers Heerspink, Emmi Tikkanen, Joel Eriksson, Andrew Wong, Massimo Mangino, Kathleen A Jablonski, Ilja M Nolte, Denise K Houston, Tarunveer Singh Ahluwalia, Peter J van der Most, Dorota Pasko, Lina Zgaga, Elisabeth Thiering, Veronique Vitart, Ross M Fraser, Jennifer E Huffman, Rudolf A de Boer, Ben Schöttker, Kai-Uwe Saum, Mark I McCarthy, Josée Dupuis, Karl-Heinz Herzig, Sylvain Sebert, Anneli Pouta, Jaana Laitinen, Marcus E Kleber, Gerjan Navis, Mattias Lorentzon, Karen Jameson, Nigel Arden, Jackie A Cooper, Jayshree Acharya, Rebecca Hardy, Olli Raitakari, Samuli Ripatti, Liana K Billings, Jari Lahti, Clive Osmond, Brenda W Penninx, Lars Rejnmark, Kurt K Lohman, Lavinia Paternoster, Ronald P Stolk, Dena G Hernandez, Liisa Byberg, Emil Hagström, Håkan Melhus, Erik Ingelsson, Dan Mellström, Östen Ljunggren, Ioanna Tzoulaki, Stela McLachlan, Evropi Theodoratou, Carla M T Tiesler, Antti Jula, Pau Navarro, Alan F Wright, Ozren Polasek, International Consortium for Blood Pressure (ICBP), Cohorts for Heart and Aging Research in Genomic Epidemiology (CHARGE) consortium, Global Blood Pressure Genetics (Global BPGen) consortium, Caroline Hayward, James F Wilson, Igor Rudan, Veikko Salomaa, Joachim Heinrich, Harry Campbell, Jacqueline F Price, Magnus Karlsson, Lars Lind, Karl Michaëlsson, Stefania Bandinelli, Timothy M Frayling, Catharina A Hartman, Thorkild I A Sørensen, Stephen B Kritchevsky, Bente Lomholt Langdahl, Johan G Eriksson, Jose C Florez, Tim D Spector, Terho Lehtimäki, Diana Kuh, Steve E Humphries, Cyrus Cooper, Claes Ohlsson, Winfried März, Martin H de Borst, Meena Kumari, Mika Kivimaki, Thomas J Wang, Chris Power, Hermann Brenner, Guri Grimnes, Pim van der Harst, Harold Snieder, Aroon D Hingorani, Stefan Pilz, John C Whittaker, Marjo-Riitta Järvelin, Elina Hyppönen

**Affiliations:** Population, Policy and Practice, UCL Institute of Child Health, London, UK (K S Vimaleswaran, PhD, A Cavadino, MSc, D J Berry, PhD, Prof. C Power, PhD, Prof. E Hyppönen, PhD); Hugh Sinclair Unit of Human Nutrition, Department of Food & Nutritional Sciences, School of Chemistry, Food & Pharmacy, University of Reading, Reading, UK (K S Vimaleswaran); Tromsø Endocrine Research Group, Department of Clinical Medicine, University of Tromsø, Tromsø, Norway (Prof. R Jorde, PhD, G Grimnes, MD); Division of Clinical Epidemiology and Aging Research, German Cancer Research Center, Heidelberg, Germany (A K Dieffenbach, PhD, B Schöttker, PhD, K-U Saum, MPH, Prof. H Brenner, MD); Department of Biostatistics, Boston University School of Public Health, Boston, MA, USA (C Lu, PhD, Prof. J Dupuis, PhD); Department of Epidemiology and Biostatistics (A C Alves, PhD, Prof. M-R Järvelin, PhD), Faculty of Medicine, School of Public Health (I Tzoulaki, PhD), and MRC-PHE Centre for Environment & Health (M-R Järvelin), Imperial College London, London, UK; Department of Clinical Pharmacology (H J L Heerspink, PhD), Department of Epidemiology (I M Nolte, PhD, Prof. H Snieder, PhD, P J van der Most, MSc, Prof. R P Stolk, PhD), Department of Psychiatry (C A Hartman, PhD), Department of Cardiology (Prof. R A de Boer, MD, P van der Harst, MD), and Department of Internal Medicine, Division of Nephrology (Prof. G Navis, MD, M H de Borst, MD), University Medical Center, University of Groningen, Groningen, Netherlands; Institute for Molecular Medicine Finland, Tukholmankatu, Finland (E Tikkanen, PhD); Centre for Bone and Arthritis Research, Institute of Medicine, Sahlgrenska University Hospital, Gothenburg, Sweden (J Eriksson, MD, Prof. M Lorentzon, MD, Prof. D Mellström, MD, Prof. C Ohlsson, MD); MRC Unit for Lifelong Health and Ageing (A Wong, PhD, Prof. R Hardy, PhD, Prof. D Kuh, PhD), Cardiovascular Genetics, BHF Laboratories, Institute of Cardiovascular Science (J A Cooper, MSc, J Acharya, PhD, Prof. S E Humphries, FRCP), Genetic Epidemiology Group (Prof. A D Hingorani, PhD), and Department of Epidemiology and Public Health (Prof. M Kumari, PhD, Prof. M Kivimaki, PhD), University College London, London, UK; Department of Twin Research & Genetic Epidemiology, King’s College London, St Thomas’ Campus, London, UK (M Mangino, PhD, Prof. T D Spector, FRCP); Biostatistics Center, Department of Epidemiology and Biostatistics, School of Public Health, George Washington University, Rockville, MD, USA (K A Jablonski, PhD); Gerontology and Geriatric Medicine, Department of Internal Medicine, and J Paul Sticht Center on Aging (D K Houston, PhD, Prof. S B Kritchevsky, PhD), and Department of Biostatistical Sciences, Division of Public Health Sciences (K K Lohman, MStat), Wake Forest School of Medicine, Winston-Salem, NC, USA; Metabolic Genetics, Novo Nordisk Foundation Centre for Basic Metabolic Research (T S Ahluwalia, PhD, Prof. T I A Sørensen, DrMedSci), and Copenhagen Prospective Studies on Asthma in Childhood (T S Ahluwalia), Faculty of Health and Medical Sciences, University of Copenhagen, Copenhagen, Denmark; Institute of Preventive Medicine, Bispebjerg and Frederiksberg Hospital, Copenhagen, Denmark (T I A Sørensen); Danish Pediatric Asthma Center, Gentofte Hospital, Copenhagen, Denmark (T S Ahluwalia); Genetics of Complex Traits, University of Exeter Medical School, Exeter, UK (Dorota Pasko, MSc, Prof. T M Frayling, PhD); Centre for Population Health Sciences (L Zgaga, PhD, Prof. H Campbell, MD, E Theodoratou, PhD, R M Fraser, PhD, J F Wilson, DPhil, Prof. I Rudan, MD, J F Price, MD, S McLachlan, MD), and MRC Human Genetics Unit, Institute of Genetics and Molecular Medicine (V Vitart, PhD, P Navarro, PhD, J E Huffman, MSc, C Hayward, PhD, Prof. A F Wright, PhD), University of Edinburgh, Edinburgh, UK; Department of Public Health and Primary Care, Trinity College Dublin, Dublin, Ireland (L Zgaga); Institute of Epidemiology I, Helmholtz Zentrum München—German Research Center for Environmental Health, Neuherberg, Germany (E Thiering, MSc, C M T Tiesler, MSc, J Heinrich, PhD); Division of Metabolic Diseases and Nutritional Medicine (E Thiering), and Institute of Medical Informatics, Biometry and Epidemiology (C M T Tiesler), Ludwig Maximilian University of Munich, Dr von Hauner Children’s Hospital, Munich, Germany; Oxford Centre for Diabetes, Endocrinology and Metabolism (Prof. M I McCarthy, MD), Wellcome Trust Centre for Human Genetics (M I McCarthy, Prof. E Ingelsson, MD), and NIHR Oxford Musculoskeletal Biomedical Research Unit (Prof. C Cooper, FMedSci, Prof. N Arden, DM), University of Oxford, Oxford, UK; Oxford NIHR Biomedical Research Centre, Oxford, UK (M I McCarthy); National Heart, Lung, and Blood Institute’s Framingham Heart Study, Framingham, MA, USA (J Dupuis); Institute of Biomedicine (Prof. K H Herzig, MD), Biocenter Oulu (K H Herzig, S Sebert, PhD, M-R Järvelin), and Institute of Health Sciences (M-R Järvelin, A C Alves, S Sebert), University of Oulu, Oulu, Finland; Medical Research Center Oulu (K H Herzig), and Obstetrics and Gynecology, Department of Clinical Sciences (A Pouta, MD), and Unit of Primary Care (M-R Järvelin), Oulu University Hospital, Oulu, Finland; National Institute for Health and Welfare, Oulu, Finland (A Pouta, M-R Järvelin); Finnish Institute of Occupational Health, Helsinki, Finland (J Laitinen, PhD); Medical Clinic V (Nephrology, Hypertensiology, Rheumatology, Endocrinology, Diabetology), Mannheim Medical Faculty, University of Heidelberg, Mannheim, Germany (M E Kleber, PhD, Prof. W März, MD); MRC Lifecourse Epidemiology Unit, University of Southampton, Southampton, UK (K Jameson, MSc, C Cooper, N Arden, Prof. C Osmond, PhD); Research Centre of Applied and Preventive Cardiovascular Medicine, University of Turku, Turku, Finland (Prof. O Raitakari, MD); Department of Clinical Physiology and Nuclear Medicine, Turku University Hospital, Turku, Finland (O Raitakari); Institute for Molecular Medicine Finland (Prof. S Ripatti, PhD, E Tikkanen), Hjelt Institute (S Ripatti, E Tikkanen), Institute of Behavioural Sciences (J Lahti, PhD), and Department of General Practice and Primary Health Care (Prof. J G Eriksson, MD), University of Helsinki, Helsinki, Finland; Department of Psychiatry, EMGO Institute, VU University Medical Centre, Amsterdam, Netherlands (Prof. B W Penninx, PhD); Center for Human Genetic Research and Diabetes Research Center (Diabetes Unit), Massachusetts General Hospital, Boston, MA, USA (L K Billings, MD, Prof. J C Florez, MD); Program in Medical and Population Genetics, Broad Institute, Cambridge, MA, USA (L K Billings, J C Florez); Department of Medicine, Harvard Medical School, Boston, MA, USA (L K Billings, J C Florez); NorthShore University HealthSystem, Evanston, IL, USA (L K Billings); Department of Endocrinology and Internal Medicine, Aarhus University Hospital, Aarhus, Denmark (L Rejnmark, MD, Prof. B L Langdahl, MD); MRC Integrative Epidemiology Unit, School of Social and Community Medicine, University of Bristol, Bristol, UK (L Paternoster, PhD); Laboratory of Neurogenetics, National Institute on Aging, National Institutes of Health, Bethesda, MD, USA (D G Hernandez, MS); Department of Surgical Sciences (L Byberg, PhD, Prof. K Michaëlsson, MD), Uppsala Clinical Research Centre, Department of Medical Sciences (E Hagström, MD, Prof. H Melhus, MD, E Ingelsson, Prof. Ö Ljunggren, MD, L Lind, MD), and Molecular Epidemiology and Science for Life Laboratory (E Ingelsson), Uppsala University, Uppsala, Sweden; Department of Chronic Disease Prevention, National Institute for Health and Welfare, Turku, Finland (A Jula, MD); Croatian Centre for Global Health, University of Split Medical School, Split, Croatia (O Polasek, MD); National Institute for Health and Welfare, Helsinki, Finland (Prof. V Salomaa, MD, J G Eriksson); Clinical and Molecular Osteoporosis Research Unit, Department of Clinical Sciences and Orthopaedic Surgery, Lund University, Skåne University Hospital, Malmö, Sweden (Prof. M Karlsson, MD); Geriatric Unit, Azienda Sanitaria Firenze, Florence, Italy (S Bandinelli, MD); Vasa Central Hospital, Vasa, Finland (J G Eriksson); Folkhälsan Research Centre, Helsinki, Finland (J G Eriksson); Unit of General Practice, Helsinki University Central Hospital, Helsinki, Finland (J G Eriksson); Department of Clinical Chemistry, Fimlab Laboratories and School of Medicine, University of Tampere, Tampere, Finland (Prof. T Lehtimäki, MD); Synlab Academy, Mannheim, Germany (W März); Division of Cardiovascular Medicine, Vanderbilt University, Nashville, TN, USA (Prof. T J Wang, MD); Department of Internal Medicine, Division of Endocrinology and Metabolism, and Clinical Institute of Medical and Chemical Laboratory Diagnostics, Medical University of Graz, Graz, Austria (Prof. S Pilz, MD, W März); Quantitative Sciences, GlaxoSmithKline, Stevenage, UK (Prof. J C Whittaker, PhD); School of Population Health, Sansom Institute for Health Research, University of South Australia, Adelaide, SA, Australia (E Hyppönen); and South Australian Health and Medical Research Institute, Adelaide, SA, Australia (E Hyppönen)

## Abstract

**Background:**

Low plasma 25-hydroxyvitamin D (25[OH]D) concentration is associated with high arterial blood pressure and hypertension risk, but whether this association is causal is unknown. We used a mendelian randomisation approach to test whether 25(OH)D concentration is causally associated with blood pressure and hypertension risk.

**Methods:**

In this mendelian randomisation study, we generated an allele score (25[OH]D synthesis score) based on variants of genes that affect 25(OH)D synthesis or substrate availability (*CYP2R1* and *DHCR7*), which we used as a proxy for 25(OH)D concentration. We meta-analysed data for up to 108 173 individuals from 35 studies in the D-CarDia collaboration to investigate associations between the allele score and blood pressure measurements. We complemented these analyses with previously published summary statistics from the International Consortium on Blood Pressure (ICBP), the Cohorts for Heart and Aging Research in Genomic Epidemiology (CHARGE) consortium, and the Global Blood Pressure Genetics (Global BPGen) consortium.

**Findings:**

In phenotypic analyses (up to n=49 363), increased 25(OH)D concentration was associated with decreased systolic blood pressure (β per 10% increase, −0·12 mm Hg, 95% CI −0·20 to −0·04; p=0·003) and reduced odds of hypertension (odds ratio [OR] 0·98, 95% CI 0·97−0·99; p=0·0003), but not with decreased diastolic blood pressure (β per 10% increase, −0·02 mm Hg, −0·08 to 0·03; p=0·37). In meta-analyses in which we combined data from D-CarDia and the ICBP (n=146 581, after exclusion of overlapping studies), each 25(OH)D-increasing allele of the synthesis score was associated with a change of −0·10 mm Hg in systolic blood pressure (−0·21 to −0·0001; p=0·0498) and a change of −0·08 mm Hg in diastolic blood pressure (−0·15 to −0·02; p=0·01). When D-CarDia and consortia data for hypertension were meta-analysed together (n=142 255), the synthesis score was associated with a reduced odds of hypertension (OR per allele, 0·98, 0·96−0·99; p=0·001). In instrumental variable analysis, each 10% increase in genetically instrumented 25(OH)D concentration was associated with a change of −0·29 mm Hg in diastolic blood pressure (−0·52 to −0·07; p=0·01), a change of −0·37 mm Hg in systolic blood pressure (−0·73 to 0·003; p=0·052), and an 8·1% decreased odds of hypertension (OR 0·92, 0·87–0·97; p=0·002).

**Interpretation:**

Increased plasma concentrations of 25(OH)D might reduce the risk of hypertension. This finding warrants further investigation in an independent, similarly powered study.

## Introduction

Low vitamin D status has been associated with an increased risk of cardiovascular disease and all-cause mortality, and the possible benefits of vitamin D supplementation are being actively investigated and debated.^1,2^ In observational studies, low plasma 25-hydroxyvitamin D (calcidiol, 25[OH]D) concentration is associated with an increased risk of hypertension.^3^ However, few large randomised controlled trials of vitamin D supplementation with primary cardiovascular outcomes have been done, and secondary analyses from other trials have provided little evidence to support an effect of vitamin D supplementation on cardiovascular outcomes.^1,4,5^ The largest of the randomised controlled studies was the Women’s Health Initiative trial^4^ (n=36 282), the results of which did not show any changes in blood pressure or hypertension after 7 years of follow-up.^4^ However, the vitamin D dose used in that trial was quite small (400 IU per day), and women in both treatment and placebo groups were allowed to take up to 1000 IU per day of additional open-label vitamin D supplementation. Some evidence for possible effects of vitamin D supplementation on blood pressure has been obtained from randomised controlled trials with higher doses^6^ and those investigating individuals with cardio-metabolic risk;^5^ however, as Elamin and colleagues have previously noted,^7^ the quality of the available evidence is “low to moderate at best”.

In this study, we explored the possible causal relation between vitamin D status and blood pressure and hypertension using a genetic approach. Mendelian randomisation exploits the fact that individual genotypes are assigned randomly at meiosis, so the effect of genetics on disease is generally unaffected by confounding or reverse causality.^8^ Recent genome-wide association studies (GWAS) have identified several variants that affect circulating concentrations of 25(OH)D.^9^ If 25(OH)D concentrations are causally involved in determining blood pressure or the risk of hypertension, then the genetic variants that affect circulating concentrations of 25(OH)D could be expected to affect blood pressure and hypertension risk. This assumption seems to be valid for at least two of the genes that affect 25(OH)D, namely *CYP2R1* (encoding cytochrome P450, family 2, subfamily R, polypeptide 1) and *DHCR7* (encoding 7-dehydrocholesterol reductase). These genes function upstream of 25(OH)D production and affect vitamin D synthesis or substrate availability.^10,11^ Two further downstream variants affect 25(OH)D, *GC* (encoding group-specific component [vitamin D binding protein]) and *CYP24A1* (encoding cytochrome P450, family 24, subfamily A, polypeptide 1), but both are known to have pleiotropic effects.^12,13^ In this study, we used genetic variants that affect vitamin D synthesis as proxy markers for lifelong differences in vitamin D status to test for a causal association with blood pressure and hypertension.

## Methods

### Study design and population

**Figure 1:**
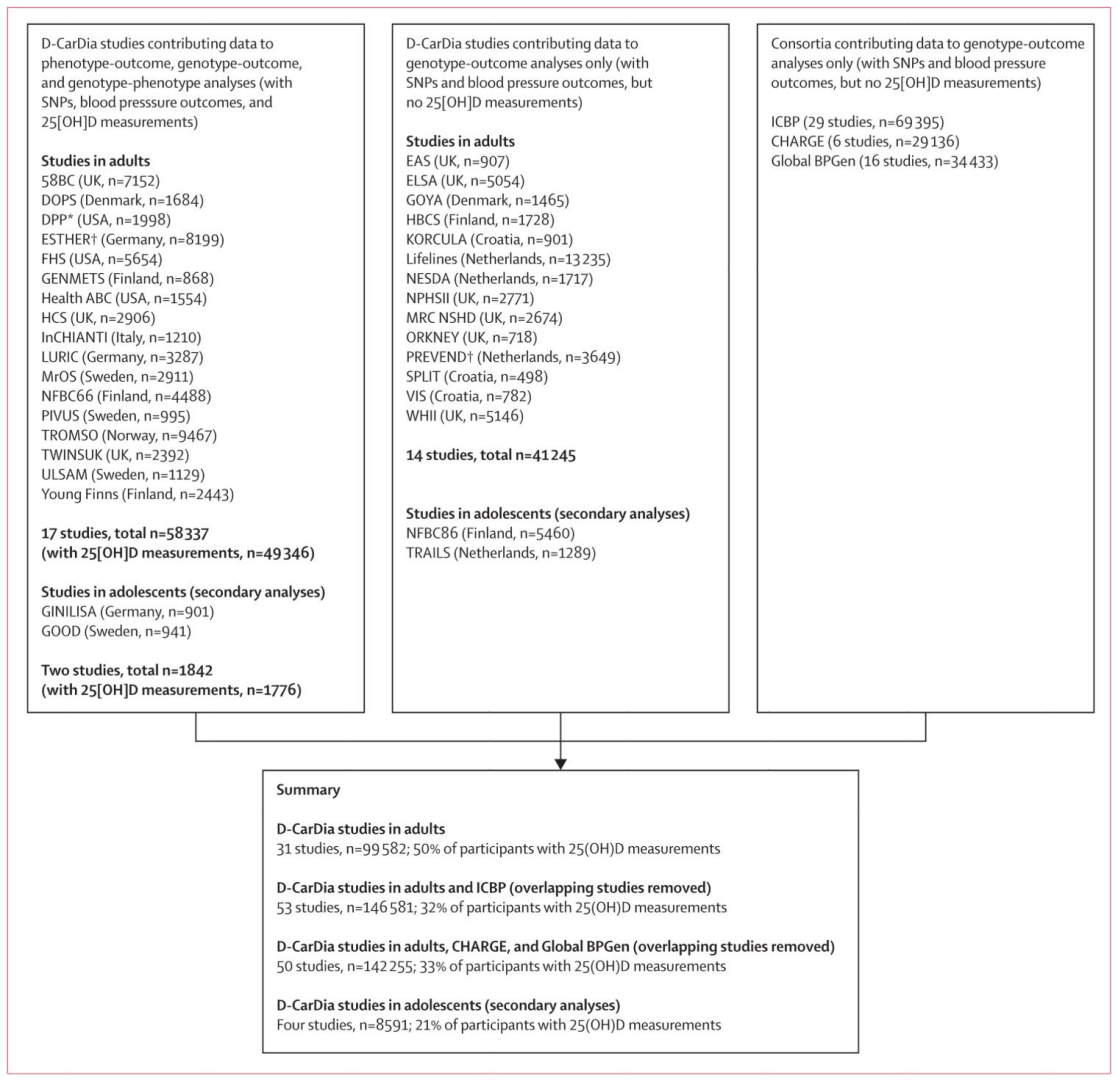
Flow chart showing the sample sizes available at each stage of the meta-analyses 25(OH)D=25-hydroxyvitamin D. ICBP=International Consortium for Blood Pressure. CHARGE=Cohorts for Heart and Aging Research in Genomic Epidemiology. Global BPGen=Global Blood Pressure Genetics. *Did not contribute data to analyses with the synthesis score single-nucleotide polymorphisms (SNPs) because of unavailability of the *CYP3R3* SNP. ^†^Did not contribute data to analyses with the metabolism score SNPs because of unavailability of *GC* or *CYP33A3* SNPs.

We used a mendelian randomisation approach to investigate the association between genetic variants that affect concentrations of circulating 25(OH)D and blood pressure measurements. We meta-analysed data from 35 studies in the D-CarDia collaboration, with results complemented by previously published summary statistics from other large-scale consortium efforts.^14–16^ D-CarDia is a collaboration of studies, consisting of cohorts of European ancestry from Europe and North America, that investigates the association of vitamin D and the risk of cardiovascular disease and related traits.^11^ We meta-analysed directly genotyped and imputed single-nucleotide polymorphisms (SNPs) from 31 adult (aged 31–92 years, n=99 582) and four adolescent (aged 10–20 years, n=8591) cohorts in the D-CarDia collaboration (table 1, figure 1). All participants provided written informed consent, and all participating studies received approval from local research ethics committees. The appendix (pp 2–19) includes descriptions of all the studies included in the analysis.

To further increase the statistical power of our study, we meta-analysed our results in adults with data from the International Consortium for Blood Pressure (ICBP)^14^ when examining systolic or diastolic blood pressure as the outcome (n=146 581, after exclusion of overlapping studies; figure 1). At the time of the study, hypertension had not been formally examined as an outcome in the ICBP consortium, and related coefficients were not available. Therefore, we used summary data from Cohorts for Heart and Aging Research in Genomic Epidemiology (CHARGE; n=29 136)^16^ and Global Blood Pressure Genetics (Global BPGen) (n=34 433)^15^ consortia when examining hypertension as the outcome (n=142 255 after exclusion of overlapping studies; figure 1).

### Phenotypic measures

Hypertension was defined as systolic blood pressure of 140 mm Hg or higher, diastolic blood pressure of 90 mm Hg or higher, or current use of antihypertensive drugs. For participants taking antihypertensive drugs, we added 15 mm Hg to systolic and 10 mm Hg to diastolic blood pressure to correct for the effect of the treatment.^14^

25(OH)D concentrations were available for 19 of the 35 studies in the D-CarDia collaboration (n=51 122), with values expressed in nmol/L for all studies. The appendix (pp 2–19) includes details about the methods used to measure 25(OH)D concentration in each study.

### Selection of SNPs and allele scores

To create vitamin D allele scores, we selected four vitamin D-related SNPs (*DHCR7* rs12785878, *CYP2R1* rs12794714, *GC* rs2282679, and *CYP24A1* rs6013897) based on the results of the GWAS by the SUNLIGHT Consortium,^9^ with two SNPs in genes located upstream (*DHCR7* and *CYP2R1*) and two downstream (*GC* and *CYP24A1*) of the 25(OH)D metabolite.^10^ All but one (*CYP2R1*) were selected as the top hit; for *CYP2R1* we used an alternative SNP also identified by the GWAS by the SUNLIGHT Consortium (p=1·84 × 10^−9^ for association with 25[OH]D concentration) because it was a functional variant in moderate linkage disequilibrium (*r^2^*=0·41) with the first-stage GWAS top hit rs10741657.^9^ The appendix (pp 2–25) includes a detailed description of the genotyping and imputation methods and effect allele frequencies for all the studies included in the meta-analysis.

We created two separate vitamin D allele scores: a synthesis allele score, created by summing the vitamin D-increasing alleles in the genes located upstream (*DHCR7* and *CYP2R1*; score range 0–4), and a metabolism allele score, created by summing the vitamin D-increasing alleles in the genes located downstream (*GC* and *CYP24A1*; score range 0–4) of the 25(OH)D metabolite.^10,11^ The synthesis allele score can be regarded as an instrument for 25(OH)D concentration when testing for causal association in mendelian randomisation analyses because it consists of variants that directly affect substrate availability or synthesis of 25(OH)D. Components included in the metabolism score are relevant for the transfer and clearance of 25(OH)D and could provide insights into the effect of vitamin D metabolism on blood pressure. However, the use of the metabolism score as a formal instrument in mendelian randomisation analyses is not possible because of problems with quantification of expected associations, pleiotropic effects,^12,13^ and the metabolic feedback loops associated with the clearance of vitamin D-related metabolites by CYP24A1.^17^ Investigations with the vitamin D metabolism score were therefore exploratory only.

### Statistical analysis

Statistical analyses in each of the D-CarDia studies were done in accordance with a standard analysis plan. We used the natural-log transformation for 25(OH)D concentrations to achieve a closer approximation of the normal distribution, and to remove non-linearity in the association with the outcomes. Additive models with systolic blood pressure, diastolic blood pressure, and hypertension as outcomes were adjusted for age, age-squared, BMI, sex, geographical region, or principal components (as relevant for the study); models with 25(OH)D concentration as the outcome were additionally adjusted for month of blood sample collection and laboratory batch, as relevant.

With respect to the phenotypic analyses, confounding factors that affect 25(OH)D concentrations were assessed previously with the 1958 British birth cohort^10^ and in selected D-CarDia studies with individual-level data (appendix pp 26–28). To assess the association of 25(OH)D concentration with systolic blood pressure, diastolic blood pressure, and hypertension, the investigators of each of the D-CarDia studies did linear regression analyses, adjusting for the key covariates as adjusted for in the additive models, and the models were repeated stratified by sex (appendix p 39).

With respect to genetic effects on 25(OH)D concentration, the effect allele was the 25(OH)D-increasing allele, as established by the SUNLIGHT Consortium.^9^ We tested the association of the four individual vitamin D-related genetic markers, and the two vitamin D allele scores, with 25(OH)D concentrations using linear regression models, adjusting for the same covariates as adjusted for in the additive models. We tested for associations of the synthesis score, and its components, with several confounders: age, sex, season, BMI, total cholesterol, and triglycerides (appendix pp 29–32). To examine variations that could affect the validity of the instruments, we used meta-regression to assess heterogeneity in the associations between the SNPs and 25(OH)D concentration by study-level factors: sex, age, method of blood pressure measurement (manual, automated, or random-zero manometer), proportion of hypertensive participants, geographical region (UK, central and southern Europe, North America, Scandinavia, or Finland), and BMI. Models were repeated with adjustment for serum triglycerides and total cholesterol (to exclude pleiotropic effects through lipid metabolism, since 25[OH]D is a cholesterol derivative) in addition to the the covariates as adjusted for in the additive models when examining systolic blood pressure, diastolic blood pressure, and hypertension as outcomes.

To examine the strength of the synthesis allele score as an instrument, we calculated the *F*-statistic from the proportion of variation in the respective phenotype (*R*^2^) explained by the allele score (*F*-statistic=(*R*^2^×(n–2))/(1–*R*^2^)].^18^ We used the inverse of the *F*-statistic to calculate the relative bias of the instrumental variable ratio compared with ordinary least-squares linear regression.^19^ Because external weights were not available and the use of internal weights could bias the instrumental variable results, we did an unweighted allele score analysis for the vitamin D SNPs (appendix p 39).

We did the formal mendelian randomisation analyses to estimate the possible causal relationship between 25(OH)D concentration and systolic blood pressure, diastolic blood pressure, and hypertension using the instrumental variable ratio method.^20^ To estimate the instrumental variable ratio for the effect of 25(OH)D concentration on systolic blood pressure, diastolic blood pressure, and hypertension, we divided the meta-analysed association of the vitamin D synthesis allele score with systolic blood pressure, diastolic blood pressure, and hypertension by the association of vitamin D synthesis allele score with 25(OH)D concentration. We estimated the variance for the instrumental variable ratio using a Taylor expansion.^21^

We used summary statistics for the four vitamin D SNPs from the ICBP,^14^ Global BPGen^15^ and CHARGE^16^ consortia to increase the statistical power of our analyses of the association between the vitamin D allele scores and blood pressure outcomes. We used an approximation method that has been previously described^14^ to combine SNPs into the synthesis and metabolism allele scores.

In the presence of heterogeneity of association between the studies, we used random-effects meta-analyses; otherwise, we tested fixed-effects models. We investigated sources of heterogeneity with univariate meta-regression models.

Four studies in the D-CarDia collaboration are in adolescents (aged 10–20 years, n=8591). The main meta-analyses were restricted to adult populations (aged 31–92 years, n=99 582), and only exploratory analyses were done in adolescents because of insufficient sample sizes (appendix pp 20, 33, and 39).

We did additional sensitivity analyses to examine the effect of adjusting for lipids (appendix p 34) of the quality of genetic information (appendix p 35), and of the adjustment applied for the use of antihypertensive drugs (appendix p 36), as well as to compare the two-stage instrumental variable ratio method with the meta-analysis of study-specific instrumental variable ratios (appendix p 37).

All meta-analyses were done at the UCL Institute of Child Health (University College London, London, UK) with Stata version 12.

### Role of the funding source

The funders of the study had no role in study design, data collection, data analysis, data interpretation, or writing of the report. The corresponding author had full access to summary data from all studies and had final responsibility for the decision to submit for publication.

## Results

All four vitamin D-related SNPs were strongly associated with 25(OH)D concentrations (p<2·22 × 10^−12^ for all comparisons; appendix p 33). As previously reported,^10,11^ the synthesis and metabolism allele scores were strongly associated with 25(OH)D concentrations (synthesis score β 2·83%, 95% CI 2·48–3·18, p=2·70 × 10^−55^, R^2^=0·5%; metabolism score β 5·38%, 4·67–6·08, p=5·93 × 10^−50^, *R*^2^=1·4%; appendix p 33). There was no evidence for heterogeneity in the association between the synthesis score and 25(OH)D concentration (*I*^2^=0%, p=0·48). Heterogeneity was seen for the metabolism score (*I*^2^=57%, p=0·003), with evidence of variation in the association between metabolism score and 25(OH)D concentration by mean study BMI (p=0·02). The *F*-statistic for the synthesis allele score was 219·7, which suggests a strong composite instrument. The relative bias of the instrumental variable ratio compared with ordinary least-square linear regression was small (0·5%).

Increased 25(OH)D concentrations were associated with reduced systolic blood pressure (β per 10% change, −0·12 mm Hg, 95% CI −0·20 to −0·04; p=0·003) and reduced odds of hypertension (odds ratio [OR] 0·98, 95% CI 0·97–0·99; p=0·0003); however, we did not see an association between 25(OH)D concentration and diastolic blood pressure (β −0·02 mm Hg, −0·08 to 0·03; p=0·37; appendix p 40). Despite evidence for heterogeneity in the phenotypic association between 25(OH)D concentration and the outcomes within the studies done in adults (systolic blood pressure, *I*^2^=73%, p=9·19×10^−07^; diastolic blood pressure, *I*^2^=78%, p=5·00×10^−09^; hypertension, *I*^2^=62%, p=0·001), the observed association between 25(OH)D concentration and systolic blood pressure, diastolic blood pressure, or hypertension between studies did not vary by age (meta-regression p≥0·09 for all comparisons), sex (meta-regression p≥0·65), method of blood pressure measurement (meta-regression p≥0·14), geographical region (meta-regression p≥0·39), or BMI (meta-regression p≥0·10). However, for the association between 25(OH)D concentration and diastolic blood pressure, there was variation across the proportion of hypertensive participants (meta-regression p=0·01).

**Figure 2:**
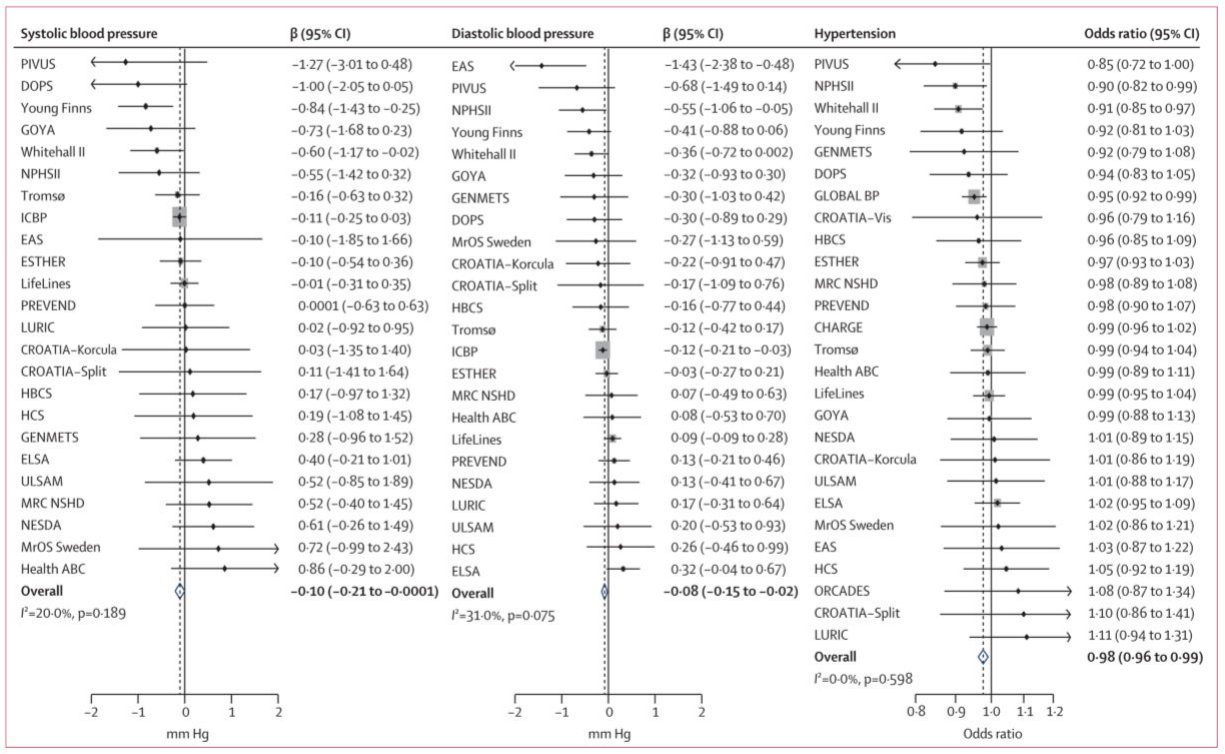
Meta-analysis of D-CarDia studies with summary data from the ICBP, CHARGE, and Global BPGen consortia Association of the synthesis score with systolic blood pressure, diastolic blood pressure, and hypertension. Includes data for 146 581 individuals, after exclusion of overlapping studies. The area of the grey boxes around a point estimate is proportional to the study’s weight in the meta-analysis. ICBP=International Consortium for Blood Pressure. CHARGE=Cohorts for Heart and Aging Research in Genomic Epidemiology. Global BPGen=Global Blood Pressure Genetics.

In the meta-analyses of the D-CarDia studies (n=108 173), there was no association of the synthesis allele score with systolic blood pressure (β per 25[OH]D-increasing allele, −0·10 mm Hg, 95% CI −0·23 to 0·02; p=0·11), diastolic blood pressure (β −0·07 mm Hg, −0·15 to 0·01; p=0·07), or hypertension (OR 0·99, 95% CI 0·97–1·00; p=0·08). After increasing the sample size by meta-analysing the D-CarDia results with the summary data from the ICBP consortium (total n=146 581, after exclusion of overlapping studies), the precision of estimation was improved, but the estimated strengths of these associations remained unchanged. The synthesis score was associated with both systolic blood pressure (β −0·10 mm Hg, −0·21 to −0·0001; p=0·0498) and diastolic blood pressure (β −0·08 mm Hg, −0·15 to −0·02; p=0·01; figure 2). For hypertension as the outcome, we meta-analysed the summary results from the CHARGE and Global BPGen consortia with the study results from adults in the D-CarDia collaboration (total n=142 255, after exclusion of overlapping studies). This analysis showed that the synthesis score was associated with hypertension (OR for increase per allele, 0·98, 0·96–0·99; p=0·001; figure 2). The metabolism allele score was not associated with any blood pressure outcomes. For analyses with maximum samples sizes, β for systolic blood pressure was −0·001 mm Hg (95% CI −0·12 to 0·12; p=0·99), β for diastolic blood pressure was 0·005 mm Hg (−0·07 to 0·08; p=0·90), and the OR for hypertension was 0·99 (0·98–1·01; p=0·48; appendix p 44).

**Figure 3:**
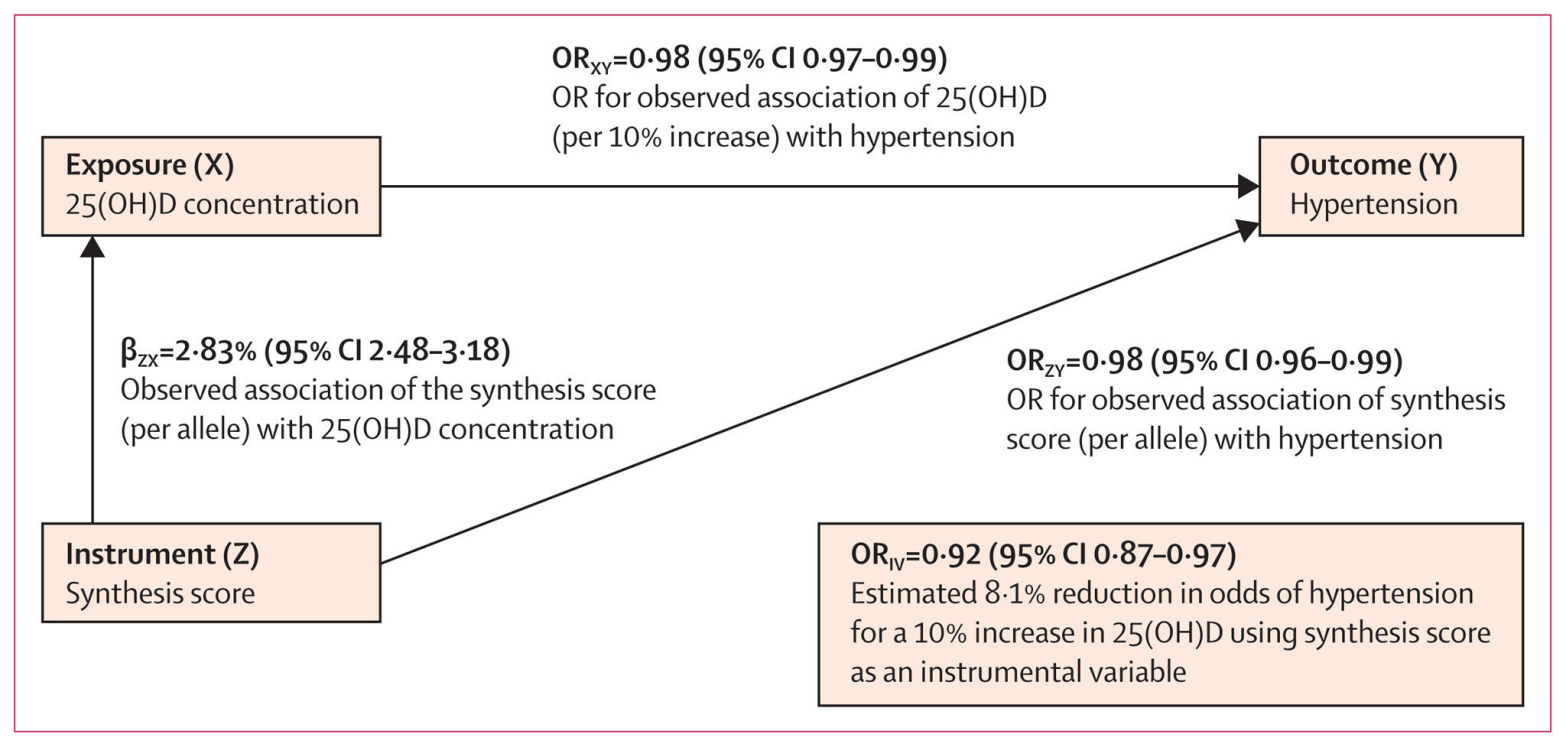
Mendelian randomisation triangulation for hypertension Instrumental variable ratio calculation done with the natural log of the odds (β_ZY_). OR=odds ratio. 25(OH)D=25-hydroxyvitamin D.

In the instrumental variable analysis, in which the synthesis score was used as an instrument, the direction of association between 25(OH)D concentration and all outcomes was compatible with that suggested by the observational phenotypic associations (table 2). Every 10% relative increment in genetically instrumented 25(OH)D concentration was associated with 0·29 mm Hg lower diastolic blood pressure (95% CI 0·07 to 0·52; p=0·01) and a 0·37 mm Hg lower systolic blood pressure (−0·003 to 0·73; p=0·052). Every 10% increment in 25(OH)D concentration was associated with an 8·1% reduced odds of hypertension (OR 0·92, 95% CI 0·87–0·97; p=0·002) in the instrumental variable ratio analyses (table 2, figure 3).

The *CYP2R1* SNP was individually associated with reduced diastolic blood pressure (per allele, β −0·09 mm Hg, 95% CI −0·18 to −0·01; p=0·03) and reduced odds of hypertension (OR for increase per allele, 0·98, 0·96–1·00; p=0·02), but no individual associations were seen for the *DHCR7* SNP, or either of the downstream metabolism SNPs (*GC* and *CYP24A1*), with any of the blood pressure outcomes (appendix pp 41–43).

## Discussion

The results of our mendelian randomisation analysis provide evidence for a causal effect of low vitamin D status on increasing blood pressure and risk of hypertension. This finding lends support to continued efforts to prevent vitamin D deficiency. In view of the costs and side-effects associated with antihypertensive drugs, the potential to reduce hypertension by vitamin D is very attractive. However, because we cannot exclude the possibility that our findings were caused by chance, they need to be replicated in an independent, similarly powered study.

Evidence from randomised controlled trials to assess the effectiveness of vitamin D supplementation in reducing blood pressure have not provided consistent evidence of a benefit.^1^ In subgroup analyses done within meta-analyses of these trials,^5,22^ some reductions in diastolic blood pressure were reported for participants with hypertension or cardiometabolic disease, and when studies that used higher doses were compared with those that used lower doses of vitamin D.^23^ Although the investigators of one study^6^ reported dose-dependent reductions in systolic blood pressure after 3 months of supplementation with 1000 IU, 2000 IU, and 4000 IU of vitamin D per day (0·66, 3·4, and 4·0 mm Hg, respectively), no effect was seen in another trial^24^ in which participants were given a bolus supplement of 100 000 IU every 3 months. These inconsistencies could be attributed to differences in the mode of administration, dose, and duration of supplementation, or to baseline differences in 25(OH)D concentrations or blood pressure, or other sources of heterogeneity between the studies. Thus, the evidence remains inconclusive. Nevertheless, these exploratory randomised controlled trials have paved the way for large trials (with upwards of 18 000 participants) that are being undertaken to examine the benefits of vitamin D for the prevention of cardiovascular disease outcomes.^25^

Our findings are biologically plausible. Inappropriate activation of the renin-angiotensin system increases blood pressure and the risk of cardiovascular disease.^26^ Studies in animals have shown that 1,25-dihydroxy-vitamin D (calcitriol, 1,25[OH] _2_D) suppresses the expression of the renin gene by a vitamin D receptor-dependent mechanism, thereby lowering blood pressure.^27^ In an open-label, blinded-endpoint trial^28^ in 101 patients with chronic heart failure who were randomly assigned to receive 2000 IU of oral vitamin D3 per day for 6 weeks or control (no treatment), treatment led to a significant decrease in plasma renin activity (p=0·002) and concentration (p=0·02). However, some findings have raised concerns about whether activation of the renin-angiotensin system has a role in the vitamin D-deficient state in human beings.^29^ Vitamin D metabolites could also exert antihypertensive effects through various other molecular mechanisms. Vitamin D is indirectly related to blood pressure through its regulation of calcium absorption from the gut and its interaction with parathyroid hormone in the maintenance of calcium homeostasis. The reno-protective and anti-inflammatory actions of vitamin D metabolites and their analogues suggest a possible role for vitamin D deficiency in cardiovascular morbidity and mortality in patients with chronic kidney disease.^30^ Furthermore, adipocyte inflammation has a crucial role in hypertension: in an in-vitro study,^31^ 1,25(OH)_2_D inhibited lipopolysaccharide-stimulated cytokine secretion in two human adipocyte models through direct inhibition of nuclear factor-κB.

On the basis of our effect size estimates, the genetic associations of the synthesis allele score with systolic or diastolic blood pressure were less pronounced than that seen for hypertension (represented as a binary trait). This finding might suggest that adequate 25(OH)D concentrations are particularly important for the prevention of hypertension as a clinical outcome. This interpretation is also supported by the results of sensitivity analyses in normotensive individuals, which showed that both the phenotypic association and the genetically indexed association between 25(OH)D concentrations and blood pressure were substantially weaker in this subgroup than in the full sample (data not shown).

Although the mendelian randomisation approach is helpful in testing for underlying causality,^20^ an imbalance in the possible overestimation or underestimation of the genetic associations for the outcome and exposure could affect the quantification of the effect.^32^ The weaker association with blood pressure than with hypertension could also reflect a greater measurement error or heterogeneity in the assessment of gradual increases across the range of the blood pressure distribution, compared with the classification of hypertension by raised blood pressure or use of antihypertensive drugs. Such noise or heterogeneity in the blood pressure measurements in our meta-analyses could also account for why the estimated strength of the associations between 25(OH)D concentrations and blood pressure outcomes were weaker than we would have expected on the basis of previous observational analyses or of power calculations that were done with data from one of the D-CarDia studies (1958 British birth cohort, n=6877).^10^

The main strength of our study is in the large sample size (up to n=146 581), which allowed us to assess the consistency of associations across several studies and to gain sufficient power for conclusive analyses. This study shows the benefits of the mendelian randomisation approach: although the phenotypic associations between 25(OH)D concentrations and blood pressure or hypertension were very heterogeneous across the studies, notably less heterogeneity was seen for the genetic associations. Age and adiposity are issues that would be expected to affect 25(OH)D concentrations and bias the phenotypic association it might have with blood pressure, but participating studies included both young and old cohorts, and both lean and obese participants. By contrast, genetic variants used in mendelian randomisation would be expected to reflect lifelong differences in 25(OH)D concentrations and would therefore be less affected by temporal variations in individual characteristics.

Potential limitations with the mendelian randomisation approach include the requirement of large sample sizes, the possibility of population stratification, canalisation, pleiotropy, and an inability to generalise findings to people in other ethnic groups. A limitation of our study was that we only looked at associations with blood pressure and hypertension, and whether these associations are related to differences in the risks of rarer disease outcomes remains uncertain. Population stratification is unlikely to have had a major effect on our main findings, since participants were all of European descent, and we adjusted for geographical region and principal components from the population stratification analysis in the statistical models.

The synthesis or metabolism SNPs might have led to biological adaptations during development (ie, canalisation),^8^ although this possibility seems unlikely in view of the similar associations with 25(OH)D seen in the analyses done in adolescents and adults. Pleiotropic effects, wherein the genetic instruments might affect other metabolic pathways independent of their influence on 25(OH)D concentration, are more likely. Our main instrument was a composite score consisting of two independent SNPs. Although differential associations between the components included in the score can suggest a possible pleiotropic effect, in our case the associations for *CYP2R1* and *DHCR7* were similar for all three outcomes (appendix p 38). Furthermore, because 25(OH)D is a secosteroid, we explicitly sought to exclude pleiotropic effects through lipid metabolism by adjusting for serum triglycerides and total cholesterol in addition to other covariates, and noted no differences in our findings (appendix p 34). However, pleiotropy is an issue with the metabolism variants included in our secondary analyses. The *GC* allele that is associated with increased 25(OH)D concentrations also leads to reduced bioavailability of active 1,25(OH)_2_D,^12^ hence increased serum concentrations of the 25(OH)D substrate are a possible consequence of reduced uptake by the cells. *CYP24A1* in turn acts as a hydroxylase for other vitamin D metabolites in addition to 25(OH)D, and the activity of the enzyme probably reflects the absolute 25(OH)D concentration, raising uncertainty about the association we would expect to see with the outcome. Indeed, we noted no evidence for associations between the metabolism SNPs and the blood pressure outcomes.

***Panel:* Research in context**

**Systematic review**

Investigators of several systematic reviews^1,5,7,23^ have summarised evidence for the phenotypic association between 25-hydroxyvitamin D (25[OH]D) and blood pressure and assessed the cardiovascular effects of vitamin D supplementation in randomised controlled trials. A prospective phenotypic association is well established, but few large randomised controlled trials of vitamin D with primary cardiovascular outcomes have been done. Evidence is largely restricted to secondary analyses of mostly small trials that were initially established to assess the effects of vitamin D supplementation on bone health. Some effects have been reported from subgroup analyses of trials focused on individuals with cardiometabolic disease,^5,22^ but the quality of the available evidence has been criticised.^7^ Previous studies that have used mendelian randomisation analyses to examine the association of 25(OH)D and cardiovascular outcomes have been underpowered and evidence for causality of association is inconclusive.^22,33^

**Interpretation**

Our results suggest that people who have genetic variants associated with low endogenous production of 25(OH)D have an increased risk of hypertension, emphasising the need for further, well-designed randomised controlled trials to assess causality and the potential clinical benefits of vitamin D supplementation. In view of the costs and side-effects associated with antihypertensive drugs, the possibility of preventing or reducing hypertension with vitamin D supplementation is very attractive. However, because we cannot exclude the possibility that the findings from this study were caused by chance, they need to be replicated in an independent, similarly powered study.

Overall, our study provides genetic evidence that increased 25(OH)D concentrations are causally associated with reduced blood pressure and hypertension risk (panel). If replicated in an independent, similarly powered study, these findings will strengthen the case for appropriately powered, well-designed randomised clinical trials to investigate the necessary vitamin D doses and appropriate target groups for the prevention or treatment of hypertension.

## Supplementary Material

Supplementary Appendix

## Figures and Tables

**Table 1:** Characteristics of the D-CarDia study cohorts, stratified by sex

	Sample size	Men				Women				All participants			
		Age (years)	Systolic blood pressure (mm Hg)	Diastolic blood pressure (mm Hg)	Participants with hyper- tension	Age (years)	Systolic blood pressure (mm Hg)	Diastolic blood pressure (mm Hg)	Participants with hyper- tension	Age (years)	Systolic blood pressure (mm Hg)	Diastolic blood pressure (mm Hg)	Participants with hyper- tension
**Studies in adults**													
NFBC 1966	4488 (2270 men, 2218 women)	31·2 (0·4)	130·5 (13·2)	80·4 (11·7)	697 (31%)	31·2 (0·3)	120·5 (19·1)	75·4 (10·7)	280 (13%)	31·2 (0·3)	125·6 (13·9)	77·9 (11·5)	977 (22%)
Young Finns	2443 (1123 men, 1320 women)	37·6 (5·1)	126·8 (14·2)	79·5 (11·8)	239 (21%)	37·6 (5·0)	117·6 (15·0)	74·0 (11·8)	167 (13%)	37·6 (5·1)	122·2 (14·6)	76·8 (11·8)	406 (17%)
NESDA	1717 (551 men, 1166 women)	43·7 (12·3)	145·1 (19·5)	85·1 (12·0)	317 (58%)	40·5 (12·7)	130·6 (19·1)	80·0 (11·6)	336 (29%)	41·5 (12·3)	135·3 (19·2)	81·6 (11·7)	653 (38%)
1958BC	7152 (3525 men, 3627 women)	45·0 (0·0)	133·7 (15·6)	82·6 (10·8)	1229 (35%)	45·0 (0·0)	120·9 (16·1)	75·9 (10·8)	580 (16%)	45·0 (0·0)	127·2 (17·1)	79·3 (11·3)	1809 (25%)
GOYA	1465 (1465 men, 0 women)	45·6 (7·1)	143·3 (19·3)	93·4 (12·3)	986 (67%)	··	··	··	··	45·6 (7·1)	143·3 (19·3)	93·4 (12·3)	986 (67%)
FHS	5654 (2677 men, 2977 women)	46·7 (12·9)	125·7 (17·1)	79·3 (10·4)	582 (22%)	46·2 (12·9)	118·4 (19·6)	73·7 (10·5)	778 (26%)	46·5 (12·9)	122·1 (18·4)	76·5 (10·5)	1360 (24%)
LifeLines	13 235 (5532 men, 7703 women)	49·0 11·7)	135·6 (16·1)	79·7 (10·0)	2186 (40%)	48·5 (11·3)	127·3 (18·0)	74·8 (9·7)	2089 (27%)	48·7 (11·7)	130·8 (17·2)	76·8 (9·8)	4275 (32%)
SPLIT	498 (213 men, 285 women)	47·9 (15·5)	134·4 (18·9)	80·9 (11·9)	80 (38%)	49·9 (13·9)	127·9 (21·4)	76·8 (12·5)	92 (32%)	49·1 (15·3)	131·2 (20·1)	78·9 (12·2)	172 (35%)
Twins UK	2392 (189 men, 2203 women)	48·6 (12·4)	130·2 (14·1)	80·3 (9·9)	53 (28%)	49·1 (12·3)	121·8 (16·0)	76·8 (10·6)	488 (22%)	49·1 (12·4)	122·6 (16·4)	77·2 (10·8)	541 (25%)
PREVEND	3649 (1880 men, 1769 women)	51·0 (13·0)	136·0 (20·0)	78·0 (11·0)	732 (39%)	48·0 (12·0)	125·0 (22·0)	72·0 (10·0)	479 (27%)	49·6 (12·9)	130·6 (21·0)	75·1 (10·5)	1211 (33%)
DOPS	1684 (0 men, 1684 women)	··	··	··	··	50·1 (2·8)	131·8 (20·6)	83·0 (11·6)	743 (44%)	50·1 (2·8)	131·8 (20·6)	83·0 (11·6)	743 (44%)
GENMETS	868 (421 men, 447 women)	49·2 (10·4)	131·6 (18·8)	83·2 (10·9)	157 (37%)	52·0 (11·6)	130·8 (20·9)	80·1 (11·1)	163 (36%)	50·7 (10·5)	131·2 (19·9)	81·6 (10·9)	320 (37%)
DPP	1998 (691 men, 1307 women)	55·1 (10·9)	129·7 (16·5)	81·7 (11·0)	264 (38%)	50·4 (10·2)	124·9 (16·8)	78·8 (10·1)	335 (26%)	51·9 (10·9)	126·5 (16·7)	79·8 (10·4)	599 (30%)
MRC NSHD	2674 (1340 men, 1334 women)	53·0 (0·0)	141·8 (21·3)	88·5 (13·1)	767 (57%)	53·0 (0·0)	134·6 (21·5)	832 (126)	574 (43%)	53·0 (0·0)	138·2 (21·4)	85·9 (12·9)	1341 (50%)
ORCADES	718 (334 men, 384 women)	54·3 (15·7)	137·5 (19·8)	80·0 (10·7)	145 (43%)	53·0 (15·7)	130·1 (22·8)	77·1 (11·6)	136 (35%)	53·6 (15·7)	133·8 (21·3)	78·6 (11·2)	281 (39%)
NPHSII	2771 (2771 men, 0 women)	56·1 (3·4)	139·7 (20·5)	85·4 (12·2)	1460 (53%)	··	··	··	··	56·1 (3·4)	139·7 (20·5)	85·4 (12·2)	1460 (53%)
KORCULA	901 (332 men, 569 women)	57·5 (14·2)	146·3 (22·1)	86·3 (10·5)	190 (57%)	55·6 (13·7)	140·6 (25·9)	82·4 (11·8)	274 (48%)	56·3 (14·2)	143·5 (24·0)	84·3 (11·2)	464 (52%)
VIS	782 (323 men, 459 women)	56·0 (15·0)	141·6 (23·1)	86·1 (14·2)	152 (47%)	57·1 (15·9)	139·9 (27·9)	83·0 (13·7)	219 (48%)	56·7 (15·1)	140·8 (25·5)	84·6 (13·9)	371 (47%)
Tromsø	9467 (4482 men, 4985 women)	58·7 (13·1)	146·4 (22·5)	85·7 (13·5)	2690 (60%)	60·4 (14·1)	147·7 (28·1)	83·2 (15·2)	2819 (57%)	59·6 (13·7)	147·1 (25·6)	84·4 (14·5)	5509 (58%)
Whitehall II	5146 (3797 men, 1349 women)	60·8 (5·9)	132·6 (18·2)	77·7 (11·6)	1556 (48%)	61·2 (6·1)	130·7 (20·6)	76·0 (12·1)	578 (43%)	60·9 (5·9)	132·1 (18·8)	77·2 (11·8)	2134 (41%)
HBCS	1728 (737 men, 991 women)	61·4 (2·8)	152·9 (22·2)	94·8 (12·2)	571 (77%)	61·6 (3·1)	149·6 (23·9)	90·9 (12·1)	713 (72%)	61·5 (2·8)	150·9 (23·2)	92·5 (12·3)	1284 (74%)
ESTHER	8199 (4598 men, 3601 women)	62·1 (6·7)	139·4 (20·2)	83·4 (10·2)	2665 (58%)	62·2 (6·5)	140·9 (18·7)	84·3 (10·4)	2260 (63%)	62·1 (6·6)	140·0 (19·6)	83·8 (10·3)	4925 (60%)
LURIC	3287 (2293 men, 994 women)	61·2 (10·7)	154·2 (23·9)	90·4 (11·9)	2146 (94%)	64·8 (10·2)	154·0 (25·9)	88·0 (12·0)	911 (92%)	62·7 (10·7)	154·2 (24·6)	89·6 (11·9)	3057 (93%)
EAS	907 (448 men, 459 women)	64·6 (5·6)	141·7 (22·7)	77·5 (11·4)	222 (50%)	64·2 (5·7)	144·5 (24·4)	76·9 (12·9)	245 (53%)	64·4 (5·6)	143·1 (23·6)	77·2 (12·2)	467 (52%)
HCS	2906 (1536 men, 1370 women)	65·7 (2·9)	139·5 (20·7)	78·4 (11·7)	855 (56%)	66·7 (2·7)	137·8 (22·7)	69·7 (12·1)	741 (54%)	66·1 (2·9)	138·7 (21·7)	74·3 (12·6)	1596 (55%)
ELSA	5054 (2299 men, 2755 women)	65·9 (9·4)	138·0 (18·7)	77·3 (11·8)	1098 (48%)	66·3 (9·8)	136·6 (20·9)	76·2 (11·3)	1261 (46%)	66·1 (9·4)	137·2 (19·9)	76·7 (11·5)	2359 (47%)
InCHIANTI	1210 (540 men, 670 women)	67·2 (15·4)	149·6 (21·8)	86·4 (10·0)	400 (74%)	69·1 (15·6)	153·2 (26·6)	86·6 (11·8)	485 (72%)	68·3 (15·4)	151·6 (24·4)	86·5 (11·0)	885 (73%)
PIVUS	995 (498 men, 497 women)	70·1 (0·2)	150·7 (25·4)	82·5 (12·2)	347 (70%)	70·2 (0·2)	157·7 (26·1)	81·0 (12·2)	366 (74%)	70·2 (0·2)	154·2 (25·9)	81·8 (12·2)	713 (72%)
ULSAM	1129 (1129 men, 0 women)	71·0 (0·64)	150·8 (21·9)	86·4 (11·7)	790 (70%)	··	··	··	··	71·0 (0·64)	150·8 (21·9)	86·4 (11·7)	790 (70%)
Health ABC	1554 (826 men, 728 women)	74·9 (2·9)	137·6 (21·3)	74·7 (12·0)	528 (64%)	74·7 (2·8)	141·6 (23·7)	73·4 (12·6)	474 (65%)	74·8 (2·9)	139·6 (22·5)	74·0 (12·3)	1002 (64%)
MrOS Sweden	2911 (2911 men, 0 women)	75·4 (3·2)	155·2 (23·9)	83·0 (12·1)	1355 (47%)	··	··	··	··	75·4 (3·2)	155·2 (23·9)	83·0 (12·1)	1335 (47%)
**Studies in adolescents**													
LISAplus and GINIplus	901 (480 men, 421 women)	10·2 (0·2)	108·5 (9·8)	61·9 (8·1)	··	10·2 (0·2)	108·8 (9·8)	63·2 (8·5)	··	10·2 (0·2)	108·6 (9·8)	62·5 (8·3)	··
NFBC 1986	5460 (2769 men, 2691 women)	16·0 (0·0)	121·1 (12·3)	68·6 (7·9)	··	16·0 (0·0)	110·2 (10·7)	66·9 (7·2)	··	16·0 (0·0)	115·6 (11·5)	67·8 (7·6)	··
TRAILS	1289 (619 men, 670 women)	16·2 (0·06)	122·1 (12·3)	60·3 (6·9)	54 (9%)	16·2 (0·7)	114·5 (11·2)	61·7 (6·8)	17 (3%)	16·2 (0·08)	118·1 (11·7)	61·0 (6·8)	71 (6%)
GOOD	941 (941 men, 0 women)	18·9 (0·6)	130·7 (13·3)	68·4 (7·9)	240 (26%)	··	··	··	··	18·9 (0·6)	130·7 (13·3)	68·4 (7·9)	240 (26%)

Data are n, mean (SD), or n (%).

**Table 2:** Summary of coefficients for instrumental variable ratio analyses, with the synthesis score as an instrumental variable

	Estimate from phenotypic analyses, per 10% increase in 25(OH)D concentration		Synthesis score with outcome, per allele	Instrumental variable estimate for causal effect, per 10% increase in 25(OH)D concentration	
	Coefficient* (95% CI)	p value	Coefficient* (95% CI)	Coefficient* (95% CI)	p value
Systolic blood pressure (mm Hg)	−0·12 (−0·20 to −0·04)	0·003	−0·10 (−0·21 to −0·0001)	−0·37 (−0·73 to 0·003)	0·052
Diastolic blood pressure (mm Hg)	−0·02 (−0·08 to 0·03)	0·374	−0·08 (−0·15 to −0·02)	−0·29 (−0·52 to −0·07)	0·01
Hypertension (odds ratio)	0·98 (0·97 to 0·99)	0·0003	0·98 (0·96 to 0·99)	0·92 (0·87 to 0·97)	0·002

Results include the D-CarDia studies (in adults only) and consortium summary statistics from the International Consortium for Blood Pressure (ICBP), the Cohorts for Heart and Aging Research in Genomic Epidemiology (CHARGE) consortium, and the Global Blood Pressure Genetics (Global BPgen) consortium. Association of the synthesis score with 25-hydroxyvitamin D (25[OH]D): coefficient per allele, 2·83% (95% CI 2·48–3·18).

* Coefficient represents the difference in blood pressure (mm Hg) or the odds ratio.
