# Supplementary material for: Association of vitamin D status with arterial blood pressure and hypertension risk: a mendelian randomisation study
Source: Lancet Diabetes Endocrinol. Author manuscript; Available in PMC 2015 Sep 25. (PMC4582411; doi:10.1016/S2213-8587(14)70113-5)
Supplement: Supplementary Appendix [file NIHMS721970-supplement-Supplementary_Appendix.pdf]

THE LANCET Diabetes & Endocrinology

Supplementary appendix

This appendix formed part of the original submission and has been peer reviewed. We post it as supplied by the authors.

Supplement to: Vimaleswaran KS, Cavadino A, Berry DJ, et al. Association of vitamin D status with arterial blood pressure and hypertension risk: a mendelian randomisation study. *Lancet Diabetes Endocrinol* 2014; published online June 26. http://dx.doi.org/10.1016/S2213-8587(14)70113-5.

Supplementary material

Index

Sr. No:	Title	Page number
**I**	Study details	2 - 19
**II**	Additional information	20
**III**	References	21, 22
**IV**	Supplementary tables 1 – 10	23-38
**V**	Supplementary figures 1 – 4	39-44
**VI**	Expanded author list	45-48
**VII**	Study acknowledgments	49-57

I. Study details

1. 1958 Birth cohort (1958BC)

Data collection: 1958BC includes all births during one week in March in England, Scotland and Wales.[1] Participants in the study have been followed regularly with information collected on a wide-range of factors related to health, lifestyle, growth and development. At age 44-45y (August 2002 to March 2004) cohort members were invited to a biomedical survey, which included measurement of weights and heights from 9,348 participants. 8,432 cohort members consented to genetic studies, of whom 7,414 also had information on serum 25(OH)D concentrations and BMI.

Measurement of serum 25(OH)D: Serum 25(OH)D concentrations were measured using automated application of the IDS OCTEIA ELISA on the Dade-Behring BEP2000 analyzer (sensitivity of 5.0nmol/L, linearity ≤155nmol/L, and intra-assay CV 5.5-7.2%), with adjustment according to the mean of the Vitamin D External Quality Assessment Scheme.[2]

Measurement of clinical parameters: Weight and standing height (n= 9,348) were measured without shoes and in light clothing by a trained nurse. Body mass index was calculated as weight (kg)/height $(m)^2$. Three blood pressure measurements were taken with the participant seated for at least 5 minutes using an Omron 705CP automated digital oscillometric sphygmomanometer (Omron, Tokyo, Japan). A large cuff was used if the mid-upper arm circumference was ≥32 cm. Measures deemed invalid by the nurse were excluded and an average of the remaining values was used in analyses. Non-fasting venous blood samples were analysed for total cholesterol and triglyceride levels by an autoanalyser (Olympus AU640, Japan) using enzymatic methods.

Genotyping: 3,000 DNA samples were selected as control subjects in the Welcome Trust Case Control Consortium 2 (WTCCC2). This includes an initial 1,500 sampled in the first round of WTCCC. The samples were genotyped on the Affymetrix 6.0 platform and the genotype calling algorithm was Chiamo.[3] Sample exclusions included relatedness, exceeding the heterozygosity thresholds, non-European ancestry, gender discrepancy, outlying allele intensities from a selection of SNPS compared to the larger sample and discordance with external genotyping. The SNP exclusions were done for MAF < 1%, statistical information from the genotyping call < 0.975, Hardy-Weinberg equilibrium (HWE) p-value < 1e-20, SNP missingness > 0.02 and gene-chip plate association 1e-5. An additional 2500 participants were genotyped by the Type 1 Diabetes Genetics Consortium on the Illumina 550K Infinium platform and the genotyping calling algorithm was Illuminus.[4] Sample exclusions were done on sample call rate > 3%, exceeding the heterozygosity thresholds, gender discrepancy and non-European ancestry. The SNP exclusions were done for MAF < 1%, HWE p-value < 1e-7 and SNP call rate <95%. The vitamin D SNPs were genotyped using the Taqman platform (Applied Biosystems, Warrington, UK).

2. CROATIA-Korcula

Data collection: The CROATIA-Korcula study, Croatia, is a family-based, cross-sectional study in the isolated island of Korcula that included 965 examinees aged 18-95. Blood samples were collected in 2007 along with many clinical and biochemical measures and lifestyle and health questionnaires.

Measurement of clinical parameters: Blood pressure was measured using standard procedures. Briefly, the subject was seated in a quiet room. Following 5 minutes of rest, blood pressure was recorded twice during the examination and the mean of the two readings was used for the analyses. Weight and standing height were measured without shoes and in light clothing by a trained research nurse using scales and stadiometer. Body mass index was calculated as kg/m^2. Fasting venous blood samples were analysed for total cholesterol and triglyceride levels.

Genotyping: The CROATIA-Korcula study genotyping used the Illumina HAP370CNV SNP chip. Genotype quality control excluded SNPs with a call rate <98%, MAF <0.01, HWE p<10e-6. Analysis was performed using GenABEL with first 3 principal components accounting for population stratification. SNPs were imputed to HapMap22, build 36, using MACHv1.16 and GenABEL derived residuals were analysed using ProbABEL.

3. CROATIA-Split

Data collection: The CROATIA-Split study, Croatia, is an on-going population-based, cross-sectional study in the Dalmatian City of Split that so far includes 535 examinees aged 18-95. Blood samples were collected in 2009 along with many clinical and biochemical measures and lifestyle and health questionnaires.

Measurement of clinical parameters: Blood pressure was measured using standard procedures. Briefly, the subject was seated in a quiet room. Following 5 minutes of rest, blood pressure was recorded twice during the examination and the mean of the two readings was used for the analyses. Weight and standing height were measured without shoes and in light clothing by a trained research nurse using scales and stadiometer. Body mass index was calculated as kg/m^2. Fasting venous blood samples were analysed for total cholesterol and triglyceride levels.

Genotyping: The CROATIA-Split study genotyping used the Illumina HAP370CNV SNP chip. Genotype quality control excluded SNPs with a call rate <98%, MAF <0.01, HWE p<10e-6. Analysis was performed using GenABEL with first 3 principal components accounting for population stratification. SNPs were imputed to HapMap22, build 36, using MACHv1.16 and GenABEL derived residuals were analysed using ProbABEL.

4. CROATIA-Vis

Data collection: The CROATIA-Vis study, Croatia, is a family-based, cross-sectional study in the isolated island of Vis that included 1,056 examinees aged 18-93. Blood samples were collected in 2003 and 2004 along with many clinical and biochemical measures and lifestyle and health questionnaires.

Measurement of clinical parameters: Blood pressure was measured using standard procedures. Briefly, the subject was seated in a quiet room. Following 5 minutes of rest, blood pressure was recorded twice during the examination and the mean of the two readings was used for the analyses. Weight and standing height were measured without shoes and in light clothing by a trained research nurse using scales and stadiometer. Body mass index was calculated as kg/m^2. Fasting venous blood samples were analysed for total cholesterol and triglyceride levels.

Genotyping: The CROATIA-Vis study genotyping used the Illumina HAP300v1 SNP chip. Genotype quality control excluded SNPs with a call rate <95%, MAF <0.01, HWE p<10e-6 . Analysis was performed using GenABEL with first 3 principal components accounting for population stratification. SNPs were imputed to HapMap22, build 36, using MACHv1.16. and GenABEL derived residuals were analysed using ProbABEL.

5. Danish Osteoporosis Prevention Study (DOPS)

Data collection: The study included 2016 participants recruited by direct mailing to a random sample (n=47,720) of 45–58 years old peri- or postmenopausal Danish women.[5] Inclusion criteria were: (1) Women with intact uterus aged 45–58 years and 3–24 months past last menstrual bleeding or experiencing perimenopausal symptoms (including irregular menstruations)—the latter combined with elevated serum follicular stimulating hormone (FSH), and (2) Hysterectomised women aged 45–52 years and having elevated FSH. Exclusion criteria were: (a) Metabolic bone disease, including osteoporosis defined as non-traumatic vertebral fractures on X-ray; (b) Current oestrogen use or oestrogen use within the past 3 months; (c) Current or past treatment with glucocorticoids >6 months; (d) Current or past malignancy; (e) Newly diagnosed or uncontrolled chronic disease; (f) Alcohol or drug addiction. Among included women, 1,716 accepted to donate a blood sample for DNA analyses. Women were included at four different study centers.

Measurement of serum 25(OH)D: All plasma 25OHD analyses were performed at the same lab using a competitive assay using rachitic rat binding protein measuring both vitamins D2 and D3 (Lund, B. & Sorensen, O.H. Measurement of 25-hydroxyvitamin D in serum and its relation to sunshine, age and vitamin D intake in the Danish population. Scandinavian Journal of Clinical Laboratory Investigations, 1979; 39, 23–30). The lower detection limit was 12,5 nmol/l. The intra- and interassay precisions were 8% and 10%, respectively.

Measurement of clinical parameters: Blood pressure was measured manually using a sphygmomanometer, with the participant in the recumbent position after 5 minuts of rest . All blood pressure measurements were performed in the morning. Women were classify as having hypertension using the following algorithm: a)

Measured blood pressure has been adjusted in user of blood pressure lowering drugs by adding 15 mmHg to SBP and 10 mmHg to DBP; b) Women have been classified as having hypertension if the reported use of blood pressure lowering drugs due to a diagnosis of hypertension or if they had an adjusted systolic blood pressure ≥140 mmHg, or an adjusted diastolic blood pressure ≥90 mmHg. Weight and standing height were measured without shoes and in light clothing by a study Investigator. Body mass index was calculated as weight (kg)/height(m^2). Non-fasting venous blood samples were analysed for total cholesterol and triglyceride levels by standard laboratory methods.

Genotyping: All genotyping was outsourced to KBiosciences who employ a novel form of competitive allele specific PCR (KASPar) system for genotyping. The SNP exclusions were done for MAF < 1%, statistical information from the genotyping call < 90%, HWE p-value < 0.01 and SNP missingness > 0.01.

6. Diabetes Prevention Program (DPP)

Data collection: The DPP enrolled 3,819 US adult (age ≥25 years) participants of multiple ethnicities with high risk criteria for the development of diabetes: overweight, elevated fasting glucose, and impaired glucose tolerance.[6] Of these participants, 3,234 were randomized to placebo, metformin 850 mg twice daily, or lifestyle intervention with a goal weight loss of ≥7% and ≥150 minutes of physical activity per week. A fourth troglitazone treatment arm (585 participants) was terminated early due to concerns for drug-related hepatotoxicity. 3,541 DPP participants consented to genetic investigation of whom 1899 had information on serum 25(OH)D concentrations. For the purposes of this study, 1083 individuals of European Ancestry (self-reported ethnicity of white or Caucasian) were examined for the vitamin D-related genetic variants and baseline blood pressure.

Measurement of serum 25(OH)D: Baseline total 25-hydroxyvitamin D levels were measured by liquid chromatography, tandem mass spectrometry (LC/MS/MS) (Waters ACQUITY UPLC with TQD triple quadruple mass spectrometer), certified through the national Institute of Standards and Technology (NIST) vitamin D quality assurance program.

Measurement of clinical parameters: BMI was measured by dividing weight in kilograms by the square of the standing height in meters. Blood pressure was measured on the upper arm with an appropriately sized cuff for arm size. The mean of two blood pressure measurements was used. Hypertension was defined according to World Health Organization guidelines as systolic blood pressure ≥140 mmHg, or diastolic blood pressure ≥90 mmHg, or current antihypertensive medication. Fasting total cholesterol and triglycerides were measured. Measurements of total plasma cholesterol and triglycerides were performed enzymatically on a chemistry autoanalyzer by using methods standardized to the Centers for Disease Control and Prevention reference methods. We obtained HDL fractions for cholesterol analysis by treating whole plasma with dextran sulfate magnesium chloride to precipitate all of the apolipoprotein B–containing lipoproteins. We calculated low-density lipoprotein cholesterol by using the Friedewald equation. In participants with triglyceride levels higher than 4.5 mmol/L (>400 mg/dL), the lipoprotein fractions were separated by using preparative ultracentrifugation of plasma by beta quantification.

Genotyping: A total of 4 vitamin D-related SNPs were genotyped in 3,541 DPP participants using the iPLEX-GOLD assay from Sequenom. SNPs were excluded based on the following thresholds: call rate <95%, or failed Hardy-Weinberg equilibrium with a P<0.001 in at least 1 ethnic group.

7. Edinburgh Artery Study (EAS)

Data collection: The EAS is a prospective study of 1592 European descent individuals (809 men and 783 women) aged 55 to 74 years enrolled in 1988.[7] Individuals were randomly selected from 11 general practices serving a range of socioeconomic and geographic areas throughout the city of Edinburgh. Participants completed a self-administered questionnaire at baseline and at 5 and 12 years of follow-up that included demographical and lifestyle variables, the WHO angina and intermittent claudication questionnaires. Individuals were also invited for a clinical examination at baseline and at 5 and 12 years after enrolment.

Measurement of clinical parameters: Standing height (without shoes) was measured to the nearest 5 mm with the use of a free-standing metal ruler on a heavy base. Weight (without shoes and outer clothing) was measured to the nearest 100 g on digital scales (Soehnle). Body mass index (BMI) was calculated as the weight in kilograms divided by square of the height in meters. Systolic and diastolic (phase V) blood pressures were recorded in the right arm only, after 10 minutes of rest with the patient in the supine position, with a Hawksley random zero sphygmomanometer. At baseline, a fasting 20-mL sample of venous blood was taken for estimation of biochemical, inflammatory, and hemostatic factors. Tests for serum total cholesterol, HDL cholesterol, and triglycerides were performed on a Cobas Bio analyzer (Roche Products) with standard kits.

Genotyping: DNA was extracted from 1007 participants attending the 5 year follow up examination. The genotyping was done through KBiosciences. The call rates were >95% and the HWE p values were > 0.18.

8. English Longitudinal Study of Ageing (ELSA)

Data collection: ELSA is a follow-up study of respondents to the UK Government's Health Survey for England (HSE) (at http://www.dh.gov.uk/), an annual cross-sectional survey designed to be representative of the community-living population. The ELSA sample included those aged ≥50 years seen originally either in HSE 1998, 1999 or 2001 with data collected by face-to-face interviewing in respondents' own homes in 2002 and 2004. Blood samples were taken for DNA analysis from those respondents who were willing and eligible to donate samples during a nurse visit following the 2004 questionnaire. There were 9432 interviewed respondents in ELSA 2004, of which 7666 volunteered for the nurse-led clinical visit, during which 6231 donated blood specimens.

Measurement of clinical parameters: Height, weight, and WC were measured during the nurse visit. Height without shoes was measured using a portable stadiometer. One measurement was taken with the informants stretching to the maximum height and the head in the Frankfort plane. Weight was measured using a portable electronic scale. Informants were asked to remove their shoes and any bulky clothing. For 14 informants weighing >130 kg, we used their estimated weights because the scales are inaccurate above this level. BMI is defined as weight (kg)/height (m^2) (ranged from 14.9 to 56.2 kg/m2). WC was defined as the midpoint between the lower rib and the upper margin of the iliac crest. Two measurements were taken using a tape with an insertion buckle at one end. Circumference was recorded to the nearest even millimetre and if the two measurements differed by >3 cm one-third was taken. The mean values of the two valid measurements (the two that were the closest to each other, if there were three measurements) used in the analyses. Blood pressure measurements were taken using an OMRON HEM-907 blood pressure monitor by a nurse. Three measurements in the seated position following 10 minutes of rest were taken. Heavy physical activity, smoking, and alcohol use were avoided for 30 minutes prior to recording the blood pressure measurement, the average of 3 measures were used in the analysis. Blood samples were collected and transported to the laboratory by post.

Genotyping: We extracted DNA from blood samples by using magnetic bead technology (Medical Solutions, Nottingham). Genotyping was performed using the KASPar methodology. The call rates and the concordance rates of the four SNPs were >98%. The HWE p values were P>0.08.

9. ESTHER

Data collection: ESTHER is a large population-based cohort study (ESTHER = "Epidemiologische Studie zu Chancen der Verhütung, Früherkennung und optimierten Therapie chronischer Erkrankungen in der älteren Bevölkerung") conducted in Saarland, Germany. Briefly, baseline recruitment was done in Saarland, Germany. Between July 2000 and December 2002 and included 9949 participants aged between 50 and 74 years. A standardized health questionnaire on sociodemographic, medical, and lifestyle factors was completed by each participant. Vital status of all participants was ascertained via local population registry of the Saarland through the end of 2010. Causes of death were verified by death certificates and coded based on ICD-10 classifications. The study was approved by the ethics committees of the medical faculty of the University of Heidelberg and of the medical board of the state of Saarland. Written informed consent was obtained from each participant.

Measurement of serum 25(OH)D: Baseline serum levels of 25(OH)D were measured for women and men in the framework of two different projects with the DiaSorin-Liason (Diasorin, Inc., Stillwater, USA) and the IDS-

iSYS (Immunodiagnostic Systems GmbH, Frankfurt Main, Germany) immunoassay in 2006 and 2010, respectively. All obtained 25(OH)D values were retrospectively standardized with liquid chromatography tandem-mass spectrometry (LC-MS/MS) in the Department of Clinical Chemistry, Canisius Wilhelma Hospital, Nijmegen, The Netherlands, as described previously.[8]

Measurement of clinical parameters: Height, weight and systolic blood pressure were assessed and documented on a standardized form by the general practitioners during the health check-up. Total cholesterol and triglycerides were measured from serum samples by standard high performance liquid chromatography methods in the central laboratory of the University Clinics of Heidelberg.[9]

Genotyping: The samples were genotyped using the TaqMan OpenArray^{TM} SNP Genotyping Platform (Applied Biosystems, Foster City, CA) according to the manufacturer's instructions. The overall call rate was 97.3%. The genotyping concordance was 100%.

10. Framingham Heart Study (FHS)

Data collection: The FHS consists of three cohorts. The original cohort was recruited in 1948 and includes 5,209 participants from Framingham, Massachusetts, US.[10] Clinical exams were conducted every other year to investigate cardiovascular disease and related risk factors. The Offspring cohort is comprised of 5,124 children of the Original cohort and the children's spouses. They were recruited in 1971, and have undergone examinations roughly every four years. A total 4,095 children of the Offspring cohort form the Third Generation cohort were enrolled in 2002. The current study sample consists of 1,815 individuals from the Offspring Generation who attended the 5th examination (1991-1995) and 3,841 from the Third Generation who attended the 1st examination (2002-2005).

Measurement of serum 25(OH)D: 25(OH)D was determined by a competitive protein-binding assay. Intra-assay CV for this assay was 7%.

Measurement of clinical parameters: BMI was derived as weight (in kilograms) divided by the square of height (in meters). Blood pressure was measured twice at rest using a mercury column sphygmomanometer by a physician, and the average from these two measures was taken. Lipid profiles were measured from morning fasting blood samples with standardized assays.

Genotyping: Genotyping was performed using the Affymetrix 500K SNP arrays supplemented with the MIPS 50K array. Samples were excluded if they had call rate < 97%, excess Mendelian errors (> 1000) or average heterozygosity outside of 5 SD of mean (< 25.758% or > 29.958%).

11. Gothenburg Osteoporosis and Obesity Determinants (GOOD) Study:

Data collection: The Gothenburg Osteoporosis and Obesity Determinants (GOOD) study was initiated to determine both environmental and genetic factors involved in the regulation of bone and fat mass.[11] Male study participants were randomly identified in the greater Gothenburg area in Sweden using national population registers, contacted by telephone, and invited to participate. To be enrolled in the GOOD study, participants had to be between 18 and 20 years of age. There were no other exclusion criteria, and 49% of the study candidates agreed to participate. A total of 921 individuals were included for the present study.

Measurement of serum 25(OH)D: Serum levels of 25-hydroxyvitamin D were measured by radioimmunoassay (DiaSorin Inc, Stillwater, MN) (n = 921). Intra-assay CV was 6%.

Measurement of clinical parameters: Height was measured using a wall-mounted stadiometer, and weight was measured to the nearest 0.1 kg. Body mass index was calculated as weight (kg)/height (m^2). Diastolic and systolic blood pressure was measured with a Omron 705CP after a 5 min rest. Measurements were performed (once) on the right arm after a 5 min rest with the subject lying down.

Genotyping: Genotyping was performed with Illumina HumanHap610 arrays at the Genetic Laboratory, Department of Internal Medicine, Erasmus Medical Center, Rotterdam, the Netherlands. Genotypes were called using the BeadStudio calling algorithm. Genotypes from 938 individuals passed the sample quality control

criteria [exclusion criteria: sample call rate <97.5%, gender discrepancy with genetic data from X-linked markers, excess autosomal heterozygosity >0.33 (~FDR < 0.1%), duplicates and/or first degree relatives identified using IBS probabilities (>97%), ethnic outliers (3 SD away from the population mean) using multi-dimensional scaling analysis with four principal components]. We sequentially discarded intensity-only markers and markers with a SNP call rate <95%.

12. Health2000 GenMets (GENMETS) Study

Data collection: The GENMETS sample is a subset of 2,212 individuals of the Health2000 study[9] collected as metabolic syndrome cases and their matched controls. The health 2000 survey was conducted in 2000 and 2001 in Finland. The survey included an interview on medical history, health-related lifestyle habits, and a clinical examination where a blood sample was drawn. For the present study, a total of 868 controls with genotype data, BMI and vitamin D measurements were included.

Measurement of serum 25(OH)D: Serum 25(OH)D concentrations were measured using 25-hydroxy Vitamin D RIA kit method (DiaSorin, Stillwater, MN, USA). The intra-assay coefficient of variation (CV) was 3,5 % and the interassay CV was 6.9 % at the concentration of 36 nmol/l. The limit of detection was 3,5 nmol/l. The serum specimens were stored frozen at - 70 C until analysed and protected from light when processed.

Measurement of clinical parameters: BMI was calculated as weight (kg)/height (m^2) using height and weight data from all possible sources [weight: self-reported weight and measured weight (digital scale or bioimpedance device); height: self-reported and measured height with wall-mounted stadiometer]. Blood pressures were measured after the subjects had been seated quietly in the measurement room for at least five minutes with standard mercury manometer (Mercuro 300; Speidel & Keller, Jungingen, Germany). Blood samples were analysed for total cholesterol and triglyceride levels by Olympus AU400 (Germany).

Genotyping: Genotyping was done on Illumina 610K arrays. 598,203 SNPs were successfully called with Illuminus software. Sample exclusions included sample call rate (<95%), relatedness, heterozygosity and gender discrepancy. The SNP exclusions were done for MAF < 1%, call rate < 95%, SNP clustering probability for each genotype < 95%, HWE $p < 1x10^{-6}$.

13. Health, Aging and Body Composition (Health ABC) Study

Data collection: The Health ABC study is a prospective cohort study investigating the associations between body composition, weight-related health conditions, and incident functional limitation in older adults.[12] Health ABC enrolled well-functioning, community-dwelling black (n=1,281) and white (n=1,794) men and women aged 70-79 years between April 1997 and June 1998. Participants were recruited from a random sample of white and all black Medicare eligible residents in the Pittsburgh, PA, and Memphis, TN, metropolitan areas in the United States. Participants have undergone annual exams and semi-annual phone interviews. The current study sample consists of 1,558 white participants who attended the second exam in 1998-1999 with available genotyping and serum 25(OH)D concentrations.

Measurement of serum 25(OH)D: Serum 25(OH)D concentrations were measured using fasting blood samples collected in the morning after a 12-hour fast, centrifuged, and stored at -80° C. Serum 25(OH)D was measured using a 2-step radioimmunoassay (25-Hydroxyvitamin D 125I RIA Kit, DiaSorin, Stillwater, Minn., USA). The inter- and intra-assay coefficients of variation for serum 25(OH)D were 6.78% and 9.16%, respectively.

Measurement of clinical parameters: Weight without shoes or heavy jewellery and wearing a standard clinic gown was measured in kilograms using a standard balance beam scale. Height without shoes was measured in millimetres using a Harpenden stadiometer (Holtain Ltd., Crosswell, United Kingdom). Body mass index was calculated as weight (in kg) / height (in m) squared. Two blood pressure measurements were taken with the participant seated for at least 5 minutes using a conventional mercury sphygmomanometer and averaged. Total cholesterol and triglycerides were determined by a colorimetric technique on a Vitros 950 analyzer (Johnson & Johnson, New Brunswick, NJ) after an overnight fast.

Genotyping: Genotyping was performed by the Center for Inherited Disease Research (CIDR) using the Illumina Human1M-Duo BeadChip system. Samples were excluded from the dataset for the reasons of sample

failure, genotypic sex mismatch, and first-degree relative of an included individual based on genotype data. Genotyping was successful in 1663 Caucasians. Analysis was restricted to SNPs with minor allele frequency ≥ 1%, call rate ≥97% and HWE p≥10-6. Genotypes were available on 914,263 high quality SNPs for imputation based on the HapMap CEU (release 22, build 36) using the MACH software (version 1.0.16).

14. Helsinki Birth Cohort Study (HBCS)

Data collection: HBCS is composed of 13,345 individuals born between the years 1934-44 in one of the two main maternity hospitals in Helsinki, Finland.[13] Between 2001 and 2003, a randomly selected sample of 928 males and 1,075 females participated in a clinical follow-up study with a focus on cardiovascular, metabolic and reproductive health, cognitive function and depressive symptoms. After quality control, genome-wide association (GWA) data is available in 1728 of the participants. Detailed information on the selection of the HBCS participants and on the study design can be found elsewhere. Research plan of the HBCS was approved by the Institutional Review Board of the National Public Health Institute and all participants have signed an informed consent.

Measurement of clinical parameters: Weight and standing height were measured without shoes and in light clothing by a trained nurse. Body mass index was calculated as weight (kg)/height(m)^2. Serum total and HDL cholesterol and triglyceride concentrations were measured using standard enzymatic methods. Blood pressure was measured from the right arm while the subject was in the sitting position and was recorded as the mean of two successive readings from a standard sphygmomanometer.

Genotyping: DNA was extracted from blood samples and genotyping was performed with a modified Illumina 610k chip by the Wellcome Trust Sanger Institute, Cambridge, UK. Relatedness of the participants was examined with the pair-wise IBD estimates and closely related individuals were excluded from the analyses. None of the participants showed non-European ancestry. Moreover, participants with X-chromosomal genotypes discrepant with the reported sex were removed. After quality control procedures 1720 samples remained for the analyses. SNPs were included in the analyses if they met the following conditions: call rate ≥ 0.95, minor allele frequency ≥ 0.01, and HWE test with P ≥1x10-5. Genomic coverage was extended to ~2.5M common SNPs by imputation using the HapMap phase II CEU data (NCBI build 36 (UCSC hg18)) as the reference sample and MACH software. SNPs with low imputation quality (r-squared < 0.30), low minor allele frequency (MAF < .01), or that diverged from HWE (1x10-5), were excluded from the analyses.

15. Hertfordshire cohort study (HCS)

Data collection: The Hertfordshire Cohort Study (HCS) has recruited 3000 men and women born in Hertfordshire UK during 1931-9 and still living there in adult life.[14] Participants in the study have been followed regularly with information collected on a wide range of factors related to health, lifestyle and infant growth. During 1998-2003 cohort members attended a research clinic, which included anthropometric data collection and blood sampling for genetic studies and serum 25(OH)D concentration. Full informed consent was obtained. In total, the present study included 2,997 individuals.

Measurement of serum 25(OH)D: Automated DiaSorin chemiluminescent assay (CV 10-12% within batch, and 10-15% between batch) was used to measure 25(OH)D.

Measurement of clinical parameters: Height was measured to the nearest 0.1cm using a Harpenden pocket stadiometer; weight was measured to the nearest 0.1kg on a SECA floor scale. Serum total and HDL cholesterol and triglyceride concentrations were measured using standard enzymatic methods. Blood pressure was measured from the right arm while the subject was in the sitting position and was recorded as the mean of two successive readings from a standard sphygmomanometer.

Genotyping: Genotyping was performed using TaqMan SNP genotyping assays (Applied Biosystems, Warrington, UK) according to the manufacturer's protocol. Genotype frequencies were in HWE (p>0.01). Call rates were >98%. 100% concordances of duplicates (n=300) were observed.

16. InCHIANTI study

Data collection: InCHIANTI is performed in two sites: Greve in Chianti (Area1; 11,709 inhabitants; >65 years: 19.3%) and Bagno a Ripoli (Village of Antella, Area2, 4704 inhabitants; >65 years: 20.3%). The data collection started in September 1998 and was completed in March 2003. 3 and 6-year follow-up assessment of the InCHIANTI study population were performed in the years 2001-2003 and 2004-2006. A nine-year follow-up is already planned. Interviews were conducted at the participants' homes. The participants signed an unformed participation consent that included permission to consult past and future administrative databases and medical charts, and conduct analyses on the blood cells, DNA and urine samples stored in the study biological bank. Participants came to study clinic for blood drawing tests, having fasted at least 8 and just concluded 24-hour urine collection. The participants received also a series of examinations including: a 5-slice peripheral quantitative computed tomography (pQCT), a surface electroneurography assessing nerve conduction velocity od the right peroneal nerve, a standard electrocardiogram, an ultrasound colour doppler examination of the carotid and vertebral arteries and of the veins of the lower limbs, and an assessments of the ankle-brachial index. On the second appointment a clinical evaluation and a comprehensive motor and cognitive performances session were performed.

Measurement of serum 25(OH)D: Serum levels of vitamin D (25OHD) were measured by radioimmunoassay (RIA kit; DiaSorin, Stillwater, MN). The intra-assay and inter-assay coefficients of variation for vitamin D were 8.1% and 10.2%, respectively.

Measurement of clinical parameters: Height and weight were measured at the study clinic, and BMI was calculated as weight (in kg) divided by the square of height (in m). Morning, fasting blood samples were collected after a 15-minute resting. Three blood pressure measurements were taken with the participant seated for at least 5 minutes using an Omron 705CP automated digital oscillometric sphygmomanometer (Omron, Tokyo, Japan). A large cuff was used if the mid-upper arm circumference was =32 cm. Measures deemed invalid by the nurse were excluded and an average of the remaining values was used in analyses. Total cholesterol and triglyceride levels were determined by commercial assays (Roche Diagnostics, Mannheim, Germany).

Genotyping: Genome-wide genotyping was performed using the Illumina Infinium HumanHap550 genotyping chip (ver1 and ver3 chips were used).[15] This product assays 550,000 unique SNPs derived primarily from staged I and II of the International Haplotype Map Project (www.HapMap.org). Experiments were performed as per the manufactures instructions using 750 ng of genomic DNA extracted from whole blood. After processing chips were scanned on Illumina BeadStation scanners. All data were analyzed in BeadStudio (version 3; Illumina), genotype calls were made using the standard cluster files provisded by Illumina. Samples were initlay assessed for genotype success rare (98%) and concordance of reported and genotype gender. Nine samples were removed from further analysis due to gender mismatch. Eighty seven samples failed the cut off genotype success rate of 98%; forty eight of these samples were re-purified and successfully genotyped, thus in total 48 samples were removed from further analysis.

17. LifeLines Cohort Study

Data collection: LifeLines is a multi-disciplinary prospective population-based cohort study examining in a unique three-generation design the health and health-related behaviours of 165,000 persons living in the North East region of The Netherlands. It employs a broad range of investigative procedures in assessing the biomedical, socio-demographic, behavioural, physical and psychological factors which contribute to the health and disease of the general population, with a special focus on multimorbidity and complex genetics. Both are community based cohort studies from the northern part of the Netherlands and have been approved by the review board of the University Medical Center Groningen. This study adheres to the principles expressed in the Declaration of Helsinki. All subjects provided written informed consent.

Measurement of clinical parameters: Height, weight, and WC were measured during the nurse visit without shoes and in light clothing. Body mass index was calculated as weight (kg)/height $(m)^2$. The measurement of serum cholesterol and triglycerides was performed using homogenous enzymatic colorimetric methods according to the manufactures instructions (Roche Diagnostics). Blood pressure has been measured using DINAMAP.

Genotyping: Genotyping for Lifelines was performed on the Illumina CytoSNP12 v2 chip. Samples were excluded based on call rates below 0.95, gender mismatch, duplicate discordance and genetic similarity. Population stratification was assessed by principal component analysis (PCA) over the sample correlation matrix, based on 16,842 independent (LD-pruned) SNPs. Samples were excluded when for they diverged from the mean with at least 3 standard deviations (Z-score > 3) for the first 5 principal components. SNPs were excluded with a minor allele frequency of <0.01, call rate <0.95, or deviation from Hardy Weinberg equilibrium ($P < 1 \times 10^{-5}$). Genome wide genotype imputation was performed using Beagle v. 3.1.0, using the NCBI build 36 of Phase II HapMap CEU data (release 22) as reference panel.

18. Lifestyle- Imune System- Allergy plus environment and genetics study (LISAplus) and German Infant Study on the influence of Nutrition Intervention plus environment and genetics (GINIplus)

Data collection: The influence of Life-style factors on the development of the Immune System and Allergies in East and West Germany PLUS the influence of traffic emissions and genetics (LISAplus) Study and the German Infant study on the influence of Nutrition Intervention PLUS environmental and genetic influences on allergy development (GINIplus) are population based birth cohort studies conducted in Germany.[16] A total of 9088 healthy, mature (gestational age over 37 weeks) neonates with a birth weight over 2500g were recruited between 1995 and 1999. Children in the studies have been followed regularly. Height and Weight was measured during a clinical examination at age 10 years. Genetic information and valid blood pressure measurements were available for 901 children, of whom 848 had also information on serum 25 (OH)D concentrations.

Measurement of serum 25(OH)D: Serum levels of vitamin D (25OHD) were measured on the fully automated Modular system E170, Roche Diagnostics, Mannheim, Germany (intra-assay CV 2.2-6.8%, inter-assay CV 4.9-11.9%).

Measurement of clinical parameters: Weight and standing height were measured without shoes and in light clothing by a study physician. Body mass index was calculated as weight (kg)/height(m)2. Blood pressure was measured twice in a sitting position from the right arm after 5 min of rest during the clinical examination at the study centres. Invalid measures were excluded and the second measurement was used. The measurement of serum cholesterol and triglycerides was performed using homogenous enzymatic colorimetric methods according to the manufactures instructions (Roche Diagnostics GmbH Mannheim). All parameters and controls were analysed on a Modular Analytics System from Roche Diagnostics GmbH Mannheim. External controls were used in accordance with the guidelines of the German Society of Clinical Chemistry and Laboratory Medicine.

Genotyping: Individual DNA was analyzed using the Affymetrix Human SNP Array 5.0 and 6.0. Genotypes were called using BRLMM-P algorithm (5.0), respectively BIRDSEED V2 algorithm (6.0), imputed after quality control (MAF>1%, HWE>1e-05, heterozygosity 4SD, call rate per SNP and person >95%) in IMPUTE2.

19. Ludwigshafen Risk and Cardiovascular Health (LURIC) Study

Data collection: The LURIC Study is a prospective cohort study among 3,316 study participants who were routinely referred to a tertiary care medical centre in south-west Germany between 1997 and 2000.[17] Inclusion criteria were the availability of a coronary angiogram, German ancestry and clinical stability with the exception of acute coronary syndromes (ACS). Exclusion criteria were any acute illness other than ACS, any chronic disease where non-cardiac disease predominated and a history of malignancy within the past five years. Patients were continuously followed up with respect to fatal events. 25(OH)D was measured in 3,299 patients of the LURIC study.

Measurement of serum 25(OH)D: 25(OH)D was measured in serum by means of a radioimmunoassy (RIA) (DiaSorin, Antony, France, and Stillwater, MN) with intra- and inter-assay coefficients of variation of 8.6 and 9.2, respectively.

Measurement of clinical parameters: Body weight was measured without shoes and in light clothing by a trained nurse. Body height was recorded to the nearest centimetre with the subject barefoot and in the upright

position. Body mass index was calculated as weight (kg)/height (m)². Body mass index was available in all 3,316 study participants. Blood pressure was measured with an automated oscillometric device (Omron MX4, Omron Healthcare GmbH, Hamburg, Germany) while supine for at least 10 min. At least three consecutive measurements of systolic and diastolic blood pressures were taken 30 seconds apart and mean values of these measurements are reported. Measurements were considered invalid and repeated if they varied > 10 mmHg systolic, > 5 mmHg diastolic, or heart rate > 5 beats per minute from each other (except for atrial fibrillation). Total cholesterol and triglycerides were measured enzymatically using WAKO reagents on a WAKO 30R analyzer (Neuss, Germany) (Wako). LDL-cholesterol and HDL-cholesterol were measured after separating lipoproteins with a combined ultracentrifugation–precipitation method (β-quantification).

Genotyping: DNA samples of 1,985 patients were genotypes with the Affymetrix Genome-Wide Human SNP Array 6.0 (Affymetrix 6.0) and 500K. Samples with low call rate (<90 %), cryptic relatedness or gender discrepancy were excluded from analyses. SNP exclusions were done for MAF<1%, HWE $<10e^{-6}$ and low call rate (<95%).

20. Males from Genetics of extremely Overweight Young Adults (GOYA)

Data collection: The study individuals consist of randomly selected control group of one in every hundred men (n= 3,601) and all extremely overweight men (n= 1,930) which were identified from the records of 362,200 Caucasian men examined at the mean age of 20 years at the draft boards in Copenhagen and its surroundings during the years 1943–77. Standing height and weight were measured at the draft board examinations. All extremely overweight men and a random sample of half the men who, were still living in the region, were invited to a follow-up survey (anthropometric measurements) in 1992–94 at the mean age of 46 years, at which time the blood samples were also taken (753 extremely overweight and 879 control men attended). The criteria for invitation to the follow-up surveys and participation have been described elsewhere.[18,19] A genome wide association study (Genetics of extremely Overweight Young Adults) was carried out recently on the blood samples available from the study participants.[20] The study was approved by the regional scientific ethics committee and by the Danish Data Protection Board.

Measurement of clinical parameters: Standing height (without shoes) and weight (only in underwears) was measured during the draftboard examination by a trained nurse. The weight was measured on an electronic scale (Lindelltronic 8000, Lindell AB, Kristianstad, Sweden). Body mass index was calculated as weight (kg)/height(m)^2. Blood pressure and pulse were measured automatically with a digital blood pressure meter with an appropriate cuff size (model UA 743, A & D, Tokyo, Japan). The final measures were a mean of three consecutive measurements, performed with one minute intervals. A large cuff was used if the mid-upper arm circumference was ≥32 cm. Measures deemed invalid by the nurse were excluded and an average of the remaining values was used in analyses. Non-fasting venous blood samples were analysed for total cholesterol and triglyceride levels by an autoanalyser (COBAS MIRA plus: Roche Diagnostic Systems GmbH, Mannheim, Germany) using enzymatic methods.

Genotyping: Of these, 673 extremely overweight and 792 control men were genotyped (and passed quality control (QC), 61 failed QC). With a sampling fraction of 0.5% (50% of 1%), these controls represent about 158,000 men among whom the case group was the most obese. Genome-wide genotyping on the Illumina 610 k quad chip was carried out at the Centre National de Genotypage (CNG), Evry, France.[20] We excluded SNPs with minor allele frequency, <1%, >5% missing genotypes or which failed an exact test of Hardy-Weinberg equilibrium (HWE) in the controls. We also excluded any individual who did not cluster with the CEU individuals (Utah residents with ancestry from northern and western Europe) in a multidimensional scaling analysis seeded with individuals from the International HapMap release 22. We carried out imputation to HapMap release 22 (CEU individuals) using Mach 1.0, Markov Chain Haplotyping.[21] Other details regarding QC of data can be found in the previous report.[20]

21. MRC National Survey of Health & Development (MRC NSHD)

Data collection: The Medical Research Council National Survey of Health and Development (MRC NSHD),[22] is an ongoing prospective birth cohort study consisting of a stratified random sample of all births in England, Scotland and Wales in one week in March 1946 (http://www.nshd.mrc.ac.uk/). The original cohort comprised

2,547 women and 2,815 men who have been followed up over 20 times since their birth. The data collected to date include cognitive and physical function, lifestyle and anthropomorphic measures and blood analytes. At age 53 years (1999), 3035 cohort members (1472 men, 1563 women) provided information. The majority (n=2989) were interviewed and examined in their own homes by trained research nurses with others completing a postal questionnaire (n=46). Of these 2734 consented to genetic studies.

Measurement of clinical parameters: During the home visits at 53 years, height, weight and blood pressure were measured and non-fasting venous blood samples were taken. Weight and standing height were measured without shoes and in light clothing according to a standard protocol; BMI was calculated as weight (kg)/height $(m)^2$. Blood pressure was measured twice, with the survey member seated and after 5 minutes of rest, using an Omron HEM-705 automated digital oscillometric sphygmomanometer (Omron); the 2nd blood pressure reading was used for this analysis. Survey members were also asked whether they had taken any prescribed medicines or tablets for high blood pressure in the last year. Total cholesterol was measured by enzymatic CHOD-PAP. Precipitation for measurement of high-density lipoprotein (HDL) cholesterol was carried out using phosphotungstic Mg2+, triglycerides were measured using a glycerol/kinase POD-linked reaction of glycerol liberated enzymatically from triglycerides. All of these measurements were made with a Bayer DAX-72. Low-density lipoprotein (LDL) cholesterol level was calculated using the Friedewald's formula: LDL cholesterol (mmol/l)=total cholesterol−HDL cholesterol–0.45×triglycerides. DNA samples were also collected at age 53 years. The data collection received MREC approval, and informed consent was given by respondents to each set of questions and measures. 2564 individuals were included in these analyses.

Genotyping: Genomic DNA was extracted using a modified salting out method (Flowgen, Sittingbourne, UK). All genotyping was outsourced to KBiosciences who employ a novel form of competitive allele specific PCR (KASPar) system for genotyping. The SNP exclusions were done for MAF < 1%, statistical information from the genotyping call < 90%, HWE p-value < 0.01 and SNP missingness > 0.01.

22. Netherlands Study of Depression and Anxiety (NESDA)

Data collection: NESDA is a multi-centre study[23] designed to examine the long-term course and consequences of depressive and anxiety disorders (http://www.nesda.nl). NESDA included both individuals with depressive and/or anxiety disorders and controls without psychiatric conditions. Inclusion criteria were age 18-65 years and self-reported western European ancestry, exclusion criteria were not being fluent in Dutch and having a primary diagnosis of another psychiatric condition (psychotic disorder, obsessive compulsive disorder, bipolar disorder, or severe substance use disorder).

Measurement of clinical parameters: Supine BP was measured twice on the right arm with an OMRON device (Omron, Tokyo, Japan) and average values of SBP and DBP were calculated. Hypertension was defined according to World Health Organization guidelines as systolic blood pressure ≥140 mmHg, or diastolic blood pressure ≥90 mmHg, or current antihypertensive medication. Individuals who used antidepressants (not serotonin-specific reuptake inhibitors) were excluded from the analyses. Data on vitamin D, cholesterol and triglycerides were not available.

Genotyping: Individual genotyping was conducted by Perlegen Sciences (Mountain View, CA, USA) using a set of four proprietary, high-density oligonucleotide arrays as part of the Genetic Association Information Network (GAIN).[24] Samples failed QC due to uncertain linkage between genotype and phenotype records, evidence of contamination, excessive missing genotype data, high genome-wide homozygosity (B75%), first- or second-degree relationships and non-Caucasian ancestry. SNPs were required not to have any of the following features: gross mapping problem, >1 genotype disagreements in 40 duplicated samples, >1 Mendelian inheritance errors in complete trio samples, minor allele frequency < 0.01 or > 0.05 missing genotypes. A Hardy–Weinberg filter was not used. A total of 435,291 SNPs met these criteria and were included in the final analysis data set.

23. Northern Finland Birth Cohort 1966 (NFBC1966) study

Data collection: NFBC1966 was originally designed to study factors affecting pre-term birth, low birth weight and subsequent morbidity and mortality.[25] Mothers living in the two northern-most provinces of Finland were invited to participate if they had expected delivery dates during 1966. A total of 12,058 live-births were in the study. At age 31 all individuals still living in the Helsinki area or Northern Finland were asked to participate in a

detailed biological and medical examination (n=6,007) as well as a questionnaire. Genotype and measured BMI data were available on 4,453 individuals in this study with multiple births being excluded.

Measurement of serum 25(OH)D: Total 25-hydroxyvitamin D was measured with high-performance liquid chromatography-tandem mass spectrometry.

Measurement of clinical parameters: Anthropometric measures, including height, weight and waist circumference were measured without shoes and in underwear by a trained nurse. Body mass index was calculated as weight (kg)/height $(m)^2$. Systolic and diastolic blood pressures were measured twice with a sphygmomanometer with a cuff size of 14 cm x 40 cm in sitting position after 15 minutes of rest. The average of the two measurements was used. Blood samples were drawn after overnight fasting. Samples were analysed within 24 hours of sampling in the Oulu University Hospital laboratory using on-going internal/external quality control. Serum total cholesterol, high-density lipoprotein (HDL)-cholesterol, low-density lipoprotein (LDL)-cholesterol and triglyceride concentrations were analysed by Hitachi 911 automatic analyzer and commercial reagents (Boehringer Mannheim, Germany).

Genotyping: For NFBC, genomic DNA was extracted from whole blood using standard methods. All DNA samples for the Illumina Infinium 370cnvDuo array were prepared for genotyping by the Broad Institute Biological Sample Repository (BSP). All individuals in the study were genotyped with call rates >95%. SNPs were excluded from analysis if the call rate in the final sample was < 95%, if the *P* value from a test of HWE was <0.0001, or if the MAF was <1%.

24. Northern Finland Birth Cohort 1986 (NFBC 1986) Study

Data collection: NFBC1986 is a longitudinal one-year birth cohort study from an unselected population. The cohort included all the mothers (N = 9,362) with children whose expected date of birth felt between July 1st 1985 - June 30th 1986 in the two northernmost provinces on Finland (Oulu and Lapland). A small percentage of the births occurred towards the end of June 1985 and begin of July 1986. The number of deliveries in the cohort was 9 362, which was 99% of all the deliveries taking place in the target period of the cohort. Altogether 9 479 children were born into the cohort, 9 432 of them live-born. The original data have been supplemented by data collected with postal questionnaires at the ages of 7, 8 and 15 / 16 years and various hospital records and statistical register data.

Measurement of clinical parameters: Weight and standing height were measured without shoes and in underwear by a trained nurse. Body mass index was calculated as weight (kg)/height $(m)^2$. Systolic and diastolic blood pressures were measured in sitting position, after 15 minutes of rest with the cuff on the right upper arm. An oscillometric pressure meter (Omron 705CP) or, if this failed, mercury sphygmomanometer were used. Three readings were taken 2 minutes apart, and the average of the three measurements was used..Blood samples were drawn after overnight fasting. Samples were analysed within 24 hours of sampling in the Oulu University Hospital laboratory using on-going internal/external quality control. Serum total cholesterol, high-density lipoprotein (HDL)-cholesterol, low-density lipoprotein (LDL)-cholesterol and triglyceride concentrations were analysed by Cobas Integra 700 automatic analyser (Roche Diagnostics, Basel, Switzerland).

Genotyping:

The NFBC1986 data were genotyped using Illumina iSelect Metabochip genotyping array. In quality control, samples were excluded due to call rate < 95%, low mean heterozygosity, duplicance (concordance with other DNA>0.99), IBS pairwise sharing >0.25, consent withdrawal, gender mismatch or being an MDS outlier. SNPs with call rate <95% or with MAF<5% and call rate <99% were excluded. After quality control, 4937 individuals and 133 991 SNPs were available for analysis.

25. Orkney Complex Disease Study (ORCADES)

Data collection: ORCADES is an on-going family-based, cross-sectional study in the isolated Scottish archipelago of Orkney. Genetic diversity in this population is decreased compared to Mainland Scotland, consistent with the high levels of endogamy historically. Data for participants aged 18-100 years, from a

subgroup of ten islands, were used for this analysis. Fasting blood samples were collected and over 300 health-related phenotypes and environmental exposures were measured in each individual. All participants gave informed consent and the study was approved by Research Ethics Committees in Orkney and Aberdeen.

Measurement of clinical parameters: Weight and standing height were measured without shoes and in light clothing by a trained research nurse using Seca scales and stadiometer. Body mass index was calculated as kg/m2. Two blood pressure measurements were taken with the participant seated for at least 5 minutes using an Omron automated digital sphygmomanometer (Omron, Tokyo, Japan). The average was used for analysis. A large cuff was used if the mid-upper arm circumference was ≥32 cm. Fasting venous blood samples were analysed for total cholesterol and triglyceride levels by an auto-analyser using enzymatic methods.

Genotyping: We genotyped 318,237 SNPs for each individual using the Illumina HumanHap300 beadchip. Alleles were called in BeadStudio using Illumina cluster files. Subjects were excluded if they fulfilled any of the following criteria: genotypic call rate <97%, mismatch between reported and genotypic sex, unexpectedly low genomic sharing with first degree relatives, excess autosomal heterozygosity, or outliers identified by IBS clustering analysis. We excluded SNPs on the basis of minor allele frequency (<0.01), HWE (P<10-5), call rate (<97%). Pregnant women were excluded from the study. MACH v1.0.15 was used to impute over 2 million SNPs from HapMap build 36.

26. Osteoporotic Fractures in Men Study – Sweden (MrOS Sweden)

Data collection: The MrOS study is a multicenter, prospective study including 3,014 elderly men in Sweden, Hong Kong (≈2,000), and the United States (≈6,000). The MrOS Sweden cohort consist of three sub-cohorts from three different Swedish cities (n=1,005 in Malmö, n=1,010 in Göteborg, and n=999 in Uppsala).[26] Study subjects (men aged 69–80 years) were randomly identified using national population registers, contacted and asked to participate. To be eligible for the study, the subjects had to be able to walk without assistance, provide self-reported data, and sign an informed consent; there were no other exclusion criteria. (1) The study was approved by the ethics committees at the Universities of Gothenburg, Lund, and Uppsala. Informed consent was obtained from all study participants.

Measurement of clinical parameters: Standard equipment was used to measure height and weight. Body mass index (BMI) was calculated as weight (kg)/height $(m)^2$. Serum lipid analysis was performed on a Konelab 20 autoanalyzer (Thermo Electron Corporation, Vantaa, Finland). Total cholesterol and triglyceride levels were determined in fasting serum by fully enzymatic techniques. With a standard mercury sphygmomanometer and a Doppler probe, we conducted duplicate measures of supine blood pressure in the right arm after subjects rested in a quiet room for at least 10 min. The study coordinator placed appropriately sized cuffs over the right upper arm, rapidly inflated them to 30 mm Hg above the audible systolic pressure, and then slowly deflated them over the artery. A hand-held Doppler (Huntleigh Mini Dopplex Model D900, Huntleigh Healthcare AB, Limhamn, Sweden) recorded the pressure in the artery as the first audible systolic pressure. After performing 2 measurements, we used the mean value of systolic pressure taken in the right brachial artery.

Genotyping: Genotyping of all SNPs was carried out on the entire MrOS Sweden cohort samples for which DNA was available (n = 2922, except rs4588 n=2445) by Kbioscience, who employ a novel form of competitive allele specific PCR (KASPar) and TaqmanTM system for genotyping. The call rates for the four SNPs were, rs10741657: 0.87, rs12785878: 0.97, rs6013897: 0.91 and rs4588: 0.81. The concordance rate was >97% and the HWE p values were > 10e-4. The SNP rs12794714 was imputed for 962 subjects using Illumina HumanOmni1_Quad_v1-0 B, with exclusion criteria: HWE-p < 10e-4, SNP call rate <0.98.

27. Prevention of Renal and Vascular Endstage Disease (PREVEND)

Data collection: The PREVEND study examines the risk factors for and the prevalence and consequences of microalbuminuria in otherwise healthy adults in the city of Groningen.[27] A total of 85,421 people were invited to participate in the study and 40,856 responded. Of these, 8,592 were invited for further screening. From 1997 on the death certificates of the PREVEND cohort of 8,592 subjects are available. Of these subjects the clinical non-fatal events are also registered using a database of hospital discharge diagnoses. In addition, these subjects are seen every 3-4 year on the out-patient PREVEND facility.

Measurement of clinical parameters: Body mass index (BMI) was calculated as the ratio between weight (kg) and the square of height (m) (weight/height2). Systolic and diastolic BP was calculated as the mean of the last two measurements of the two visits. Serum cholesterol and triglycerides were determined by Kodak Ektachem dry chemistry (Eastman Kodak, Rochester, NY, USA), an automated enzymatic method.

Genotyping: DNA from 3,649 subjects was available for GWA analysis. GWA were carried out using commercial arrays, with imputation of missing genotypes to HapMap2. SNPs with low minor allele frequencies (<1%), call rates (<95%) or imputation quality (.info (IMPUTE) metrics <0.80) were excluded.

28. Prospective Investigation of the Vasculature in Uppsala Seniors (PIVUS):
Data collection: The PIVUS (http://www.medsci.uu.se/pivus/) has been described in detail previously.[28] All 70-year-old men and women living in Uppsala, Sweden, were eligible for the study, and 2025 randomly selected individuals were invited within 2 months of their 70th birthday from April 2001 to June 2004. Of these, 1016 (50%) participated in the study. At the examination the participants underwent a blood pressure measurement and anthropometry, blood sampling after an overnight fast, routine medical history. In total, the present study included 1,013 individuals.

Measurement of serum 25(OH)D: 25-hydroxyvitamin D2 and D3 in serum were measured as a standard procedure at the department of Clinical Chemistry at Uppsala University Hospital. The LIAISON® 25-hydroxyvitamin D Assay (DiaSorin) uses chemiluminescent immunoassay technology. CV for inter-assay analyses is 18.4% at a 25-hydroxyvitamin D level of 39.5 nmol/L and 11.7% at 121.2 nmol/L. The intra-assay CV is 7.1% at 44.7 nmol/L and 3.6% at 120.0 nmol/L.

Measurement of clinical parameters: Weight and standing height (n=1194) were measured without shoes and in light clothing by a trained nurse. Body mass index was calculated as weight (kg)/height $(m)^2$. Blood pressure was measured in a supine position manually by a calibrated mercury sphygmomanometer (to the nearest mmHg) after at least 30 min of rest and the average of three recordings was used. Triglycerides and total cholesterol was measured with routine methods. Cholesterol and triglycerides concentrations were analyzed in serum and HDL was separated by precipitation with magnesium chloride/phototungstate by enzymatic techniques using IL Test Cholesterol Trinder's Method and IL Enzymatic-colorimetric Method for use in a Monarch apparatus (Instrumentation Laboratories, Lexington, USA).

Genotyping: The SNPs were genotyped as part of a larger panels of SNPs at the SNP technology platform at Uppsala University (http://www.genotyping.se/) on an Illumina BeadStation 500GX using Infinium iSelect and Golden Gate assays from Illumina Inc.[29] Genotyping calls were performed with Illumina BeadStudio or GenCall software. Samples with low call rate (<90 %), excess heterozygosity or cryptic relatedness were excluded from analyses. SNPs with a call rate less than 90%, or that failed HWE (exact P-value<$1x10^{-6}$) were excluded from the study.

29. Second Northwick Park Heart Study (NPHSII)

Data collection: NPHSII is a prospective study of unrelated healthy middle-aged Caucasian male subjects (age 49-64 years) recruited between April 1989 and April 1994 from nine United Kingdom general practices.[30] Exclusion criteria were a history of MI, cerebrovascular disease, life-threatening malignancy or regular medication with aspirin or anticoagulants. Each man answered a questionnaire detailing lifestyle and medical history. At entry, systolic and diastolic (Korotkoff V) blood pressures (SBP and DBP respectively) were recorded twice with a random zero mercury sphygmomanometer after the subject had been seated for 5 minutes.

Measurement of clinical parameters: At entry, systolic and diastolic (Korotkoff V) blood pressures (SBP and DBP respectively) were recorded twice with a random zero mercury sphygmomanometer after the subject had been seated for 5 minutes. The average of the two measurements was used for analysis. Serum cholesterol and triglyceride levels were determined by automated procedures (Sigma and Wako chemicals, respectively). The men did not fast for the study but had been instructed to avoid heavy meals. They were also requested to refrain from smoking and vigorous exercise from midnight before the examination. Height was recorded to the nearest 0.1cm and weight to the nearest 0.1kg without shoes or outdoor clothing. Body mass index was calculated as weight/height^2.

Genotyping: DNA was extracted from whole blood using the salting out method. Genotyping was performed using TaqMan technology (Applied Biosciences (ABI), Warrington UK). The call rate for all the 4 SNPs was >98 % and all SNPs were in Hardy-Weinberg equilibrium.

30. TRacking Adolescents' Individual Lives Survey (TRAILS)

Data collection: TRAILS is a prospective cohort study of Dutch adolescents with bi- or triennial measurements from age 11 to at least age 25 and consists of a general population and a clinical cohort.[31] In the population cohort, four assessment waves have been completed to date, which ran from March 2001 to July 2002 (T1), September 2003 to December 2004 (T2), September 2005 to August 2007 (T3), and October 2008 to September 2010 (T4). Data for the present study were collected during the third assessment wave. At T1, 2230 (pre)adolescents were enrolled in the study (response rate 76.0%, mean age 11.09, SD 0.55, 50.8% girls, of whom 81.4% (N = 1816, mean age 16.27, SD 0.73, 52.3% girls) participated at T3.

Measurement of clinical parameters: Systolic and diastolic blood pressure were measured in duplicate with a Dinamap Critikon 1846SX (Critikon Inc, Tampa, FL), from which we calculated means. Hypertension was defined according to World Health Organization guidelines as systolic blood pressure ≥140 mmHg, or diastolic blood pressure ≥90 mmHg. None of the adolescents used antihypertensive medication. The number of hypertensive cases was too small to be analyzed reliably. We obtained a blood sample after >8 h of fasting for the measurement of triglycerides, total cholesterol, and HDL cholesterol (Roche Diagnostics). Non-fasting blood samples were excluded from the analyses.

Genotyping: Genome-wide genotyping for 1491 individuals was done with the Illumina Cyto SNP12 v2 array and genotypes were called using Illumina's GenomeStudio. Samples were excluded when: call rate <95%, heterozygosity >4SD, duplicates, sex mismatch, or non-caucasian. SNPs were removed when: call rate < 95%, MAF<1%, HWE p-value<1e-04, chr X SNPs >1% heterozygous in men. The QC-ed data of 1352 individuals were imputed using IMPUTE2 and association analysis was performed with SNPTEST v2.2.0.

31. Tromsø study

Data collection: The Tromsø Study, conducted by the University of Tromsø in cooperation with the National Health Screening Service, is a longitudinal population-based multipurpose study focusing on lifestyle related diseases.[32] The fourth survey was performed in 1994 and 27 158 subjects attended. A total of 9528 subjects were selected for participation in the present study and DNA successfully prepared and analyzed for at least one SNP in sera from 1994 in 9471 subjects. Among these, 4175 were included as control cohort to the rest of the subjects that were included because of diagnosed myocardial infarction, cancer, type 2 diabetes or death.

Measurement of serum 25(OH)D: Serum levels of 25(OH)D_3 were measured by an electrochemiluminescence immunoassay (ECLIA), using an automated clinical chemistry analyser (Modular E170, Roche Diagnostics®, Mannheim, Germany). The total analytical coefficient of variation (CV) for the 25(OH)D_3 assay was 7.3 %.

Measurement of clinical parameters: Height and weight were measured while the subjects wore light clothing and no shoes and Body mass index (BMI) calculated as kg/m2. Blood pressure was measured with an automatic device (Dinamap Vital Signs Monitor 1846; Critikon Inc, Tampa, FL). The subjects were seated for 2 min. Three recordings were made at 2-min intervals, and the lowest of the two last value was used. Serum total cholesterol and triglycerides were analyzed by enzymatic colorimetric methods with commercial kits (CHOD-PAP for cholesterol and GPO-PAP for triglycerides; Boehringer-Mannheim, Mannheim, FRG). The reference range for cholesterol in our laboratory is 3.2 to 7.4 mmol/L for those aged 25 to 29 years, 3.7 to 8.3 mmol/L for those aged 30 to 39 years, and 4.1 to 8.7 mmol/L for those aged > 40 years. The reference range for triglycerides is 0.20 to 1.80 mmol/L.

Genotyping: All genotyping was performed by KBioscience (http://www.kbioscience.co.uk) using KASP (KBioScience Allele-Specific Polymorphism) SNP genotyping system. KASP is a competitive allele-specific PCR incorporating a FRET (Fluorescence Resonance Energy Transfer) quencher cassette. In all genotyping plates no-template controls (NTCs) are included to demonstrate that any amplification in the sample wells is due solely to the presence of the sample DNA. Two separate manual quality control checks are performed, and the

data is also checked by specific software to determine that there are no incorrect call assignments, no samples too close or too far from the origin, no NTCs amplified, or any incorrect calls. The call rate for all the 4 SNPs was >98 % and the Hardy-Weinberg equilibrium P value was > 0.01.

32. Twins UK

Data collection: The Twins registry in St. Thomas' Hospital, King's College London recruited a total sample of 11,000 identical and non-identical, mostly female Caucasian, twins from across the UK through national media campaigns.[33] Their age ranges between 16 and 85 years. Over 7,000 twins have attended detailed clinical examinations with a wide range of phenotypes over last 18 years. All participants were recruited without presence or interest in any particular disease or trait. We used 1,930 individuals for the present study.

Measurement of serum 25(OH)D: Total 25-hydroxy vitamin D levels were measured by radioimmunoassay using Diasorin RIA kit (Diasorin, Minnesota, USA). This assay has a detection limit of 4 nmol/L and CV of 9.1% at 22 nmol/L.

Measurement of clinical parameters: Weight and standing height were measured without shoes and in light clothing by a trained nurse. Body mass index was calculated as weight (kg)/height (m)^2. Three blood pressure measurements were taken with the participant seated for at least 5 minutes. Hypertension was defined according to World Health Organization guidelines as systolic blood pressure ≥140 mmHg, or diastolic blood pressure ≥90 mmHg, or current antihypertensive medication. Non-fasting venous blood samples were analysed for total cholesterol and triglyceride levels by an auto-analyser (Olympus AU640, Japan) using enzymatic methods.

Genotyping: Genotyping in the TwinsUK study was performed using Illumina 610K SNP. Analyses were restricted to SNPs with call rate of genotype ≥ 98% with average heterozygosity within 3 SD of mean (< 0.3042438 and > 0.3267991). MAF was set as >1% and HWE P > 10-4. IMPUTE software was used for imputation.

33. Uppsala Longitudinal Study of Adult Men (ULSAM)

Data collection: The ULSAM was initiated in 1970 when all men born during the years 1920-24 and living in Uppsala, Sweden were invited at age 50 to participate in a health survey focusing on identifying cardiovascular risk factors (http://www.pubcare.uu.se/ULSAM).[34] The present analyses are based on measurements made at the third examination cycle of the ULSAM cohort, when participants were approximately 71 years old (1991-95, n=1221). Of these, 1,194 participants had measurements of plasma 25(OH)D and were included in this study.

Measurement of serum 25(OH)D: Total plasma 25(OH)D, including 25-hydroxyvitamin D_3 and D_2, was determined with high-pressure liquid chromatography (HPLC) atmospheric pressure chemical ionization (APCI) mass spectrometry (MS) at Vitas, Oslo, Norway (www.vitas.no). HPLC was performed with a Hewlett Packard 1100 liquid chromatography (Agilent Technologies, Palo Alto CA, USA) interfaced by APCI to a Hewlett Packard mass spectrometer operated in single-ion monitoring mode (SIM). Recovery is 95%; the method is linear from 5-400 nmol/L and the limit of detection is 1-4 nmol/L. The Coefficients of Variation (CV) for inter-assay analyses are 7.6% at 25-hydroxyvitamin D concentrations of 47.8 nmol/L and 6.9% at 83.0 nmol/L. The intra-assay CV was 5.1% at 47.8 nmol/L and 6.1% at 83.0 nmol/L. The assay is accredited by the Vitamin D External Quality Assessment Scheme (DEQAS).

Measurement of clinical parameters: Weight and standing height (n= 1,194) were measured without shoes and in light clothing by a trained nurse. Body mass index was calculated as weight (kg)/height (m)^2. After the subject had rested for 10 minutes, blood pressure was measured with the subject in supine position. Systolic and diastolic blood pressures, defined as Korotkoff phases I and V, respectively, were recorded twice and to the nearest even mmHg. The mean of the two measurements were used. We measured fasting concentrations of serum triglycerides and total cholesterol by using IL Test Cholesterol Trinders' Method and IL Test Enzymatic-Colorimetric Method in a Monarch apparatus (Instrumentation Laboratories, Lexington, USA).

Genotyping: The SNPs were genotyped as part of a larger panels of SNPs at the SNP technology platform at Uppsala University (http://www.genotyping.se/) on an Illumina BeadStation 500GX using Infinium iSelect and Golden Gate assays from Illumina Inc.. Genotyping calls were performed with Illumina BeadStudio or GenCall software. Samples with low call rate (<90 %), excess heterozygosity or cryptic relatedness were excluded from

analyses. SNPs with a call rate less than 90%, or that failed (exact P-value<$1x10^{-6}$) were excluded from the study.

34. Whitehall II (WII) Study

Data collection: The Whitehall II study recruited 10,308 participants (70% men) between 1985 and 1989 from 20 London-based Civil Service departments (Marmot et al., 1991; Marmot & Brunner, 2005). At phases 3 (1991-3), 5 (1997-1999) and 7 (2002-2004) all participants known to be alive and in the country were invited to a clinic for anthropometric and biochemical screening. Of the baseline participants, 6156 participated in phase 7 screening, at which DNA was collected.

Measurement of clinical parameters: Weight was measured in underwear to the nearest 0.1 kg on Soehnle electronic scales. We measured height in bare feet to the nearest 1 mm by using a stadiometer with the participant standing erect with head in the Frankfort plane. We calculated body mass index as weight (kilograms)/height (metres) squared. We measured waist circumference, taken as the smallest circumference at or below the costal margin, with participants unclothed in the standing position by using a fibreglass tape measure at 600 g tension. We measured systolic blood pressure and diastolic blood pressure twice in the sitting position after five minutes' rest with the Hawksley random zero sphygmomanometer. We took the average of the two readings to be the measured blood pressure. We took venous blood in the fasting state or at least five hours after a light, fat free breakfast, before a two hour 75 g oral glucose tolerance test was done. Serum for lipid analyses was refrigerated at −4°C and assayed within 72 hours. We used a Cobas Fara centrifugal analyser (Roche Diagnostics System, Nutley, NJ) to measure cholesterol and triglyceride concentrations.

Genotyping: We extracted DNA from blood samples by using magnetic bead technology (Medical Solutions, Nottingham). Genotyping was performed using the Illumina 50K IBC CVD chip or KASPar methodology. The call rates and the concordance rates of the four SNPs were >98%. The HWE p values were P>0.07.

35. Young Finns Study:

Data collection: The first cross-sectional survey was conducted in 1980 all around Finland.[35] Total sample size was 4,320 boys and girls in 6 age cohorts (aged 3, 6, 9, 12, 15 and 18). These individuals were randomly chosen from the national register. A total of 3,596 individuals (83.2% of those invited) participated the study in 1980. After that, several follow-up studies of this cohort have been conducted. The latest follow-up was performed in 2007, when the study subjects had reached the age of 30 to 45 years. In the latest follow-up in 2007 a total of 2,204 subjects were examined.[36] For the present study, we included 2,442 individuals with genotype, BMI and vitamin D measurements.

Measurement of serum 25(OH)D: Serum 25(OH)D concentrations were determined using radioimmunoassay (DiaSorin, Stillwater, Minnesota). The detection limit was 3.8 nmol/l, sensitivity = 1 nmol/l, measurement range = 12.5 - 250 nmol/l, inter-assay CV 8.0% (n = 137) and intra-assay CV 1.8 – 11.0% (n = 5) at the mean level of 43.8 nmol/l).

Measurement of BMI: Weight and standing height (n=2170) were measured without shoes and in light clothing by a trained nurse. Body mass index was calculated as weight (kg)/height $(m)^2$. Blood pressure was measured with a standard mercury sphygmomanometer in 1980 and 1983. In 1980, among 3-year-olds an ultrasound device was used. In 1986, 2001, and 2007, a random zero sphygmomanometer was used. The average of three measurements was always used in statistical analyses. Venous blood samples were drawn after an overnight fast. Standard methods were used for serum total cholesterol, triglycerides, and HDL-cholesterol. LDL-cholesterol concentration was calculated using Friedewald equation.

Genotyping: The genome-wide single nucleotide polymorphisms (SNP) genotyping of YF-study was done by a custom Illumina BeadChip containing 670,000 SNPs and copy-number variant (CNV) probes from 2,442 YF participants (1,123 males, 1,319 females). The custom 670K chip shares 562,643 SNPs in common with the Illumina Human610 BeadChip. Genotypes were called using Illumina's clustering algorithm (Illuminus). A total of 2,556 samples were genotyped. After initial clustering, we removed 2 individuals for poor call rates (CR<0.90), and 54 samples failed subsequent quality control (QC) (i.e., duplicated samples, heterozygosity, low call rate, or custom SNP fingerprint genotype discrepancy). The following filters were applied to the remaining

data: MAF 0.01, GENO 0.05, MIND 0.05, and HWE 1 x 10-6. Three of 2,500 individuals were removed for low genotyping (MIND > 0.05), 11,766 markers were excluded based on HWE test (P ≤ 1 x 10-6), 7,746 SNPs failed missingness test (GENO > 0.05), 34,596 SNPs failed frequency test (MAF < 0.01) and one individual failed gender check. None were removed by subsequent heterozygosity check. In that point, there were 546,770 SNPs and 2,496 individuals which were utilized to generate an identity-by-descent (IBD) matrix file in PLINK. There were 51 pairs of individuals with pi-hat greater than 0.2 thus these individuals removed due to possible relatedness. One of the pair was removed using greater missingness as criteria. After final frequency and genotyping running, there was 546,677 SNPs available from a sample of 2442 Young Finns individuals. Genotype imputation was performed for the YF SNP data using MACH with the HapMap (phase II, release 22 CEU, NCBI build 36, dbSNP 126) haplotypes as reference.

II. Additional information

1. ***Genotyping***:

The studies that did not have genotyped data analysed imputed or proxy SNPs (r^2=1) as available (with a call threshold of 0.9 for the SNPs imputed with Impute; for those imputed with MACH, a call threshold of 0.8 was used). The genetic data for most studies was obtained from GWA platforms, but for some studies (EAS, HCS, DOPS, NFBC 1986, NPHSII, ELSA, WHII, DPP and MRC NSHD), variants were genotyped *de novo*.

2. ***Overlap between D-CarDia and consortia-based studies***:

To increase the statistical power of our study, we meta-analysed our results with the data from the International Consortium for Blood Pressure (ICBP)[37] when examining systolic (SBP) or diastolic (DBP) blood pressure as the outcome. ICBP comprises 69,395 individuals of European ancestry from 29 studies,[37] and it included the 1958BC, FHS, NFBC1966, Twins UK, ORCADES, CROATIA-VIS and InCHIANTI, which were also part of the D-CarDia collaboration. These overlapping studies were therefore excluded when meta-analyzing D-CarDia studies with the ICBP summary data (total N=146,581 after excluding overlapping studies). At the time of the study, hypertension had not been formally examined as an outcome in the ICBP consortium, and related coefficients were not available. Therefore, we used summary data from Cohorts for Heart and Aging Research in Genomic Epidemiology (CHARGE)[38] (N=29,136) and Global BPgen (N=34,433)[39] consortia when examining hypertension as the outcome. Studies that overlapped with D-CarDia collaboration were again excluded in the meta-analyses (CHARGE: FHS; Global BPgen: 1958BC, NFBC1966, InCHIANTI and Twins UK; combining D-CarDia, CHARGE and Global BPgen, total N=142,255 after excluding overlapping studies).

3. ***Analyses on adolescents***:

Two studies on adolescents (GINILISA and GOOD, N=1,776) were available for investigating associations with 25(OH)D, and four studies (GINILISA, GOOD, NFBC86 and TRAILS, N=8,591) for evaluating genetic associations with SBP and DBP (Figure 1).

Analyses on adolescents confirmed the associations between the synthesis and metabolism scores with 25(OH)D [synthesis score: beta per allele 3·83% (95%CI: 2·43%,5·22%), P=$7{\cdot}3\text{x}10^{-8}$; metabolism score: beta 6·99 (95%CI: 2·84%,11·14%), P=0·001] (Supplementary table 4). However, there was no phenotypic association between 25(OH)D and SBP [beta per 10% change 0·001 mmHg (95%CI: -0·03,0·03), P=0·94] or DBP [beta 0·01 mmHg (95%CI: -0·01,0·02), P=0·36]. Furthermore, there was no evidence for an association between synthesis or metabolism scores with SBP or DBP [synthesis score: SBP, beta 0·11 mmHg (95%CI: -0·14,0·35), P=0·39 and DBP, beta -0·02 mmHg (95%CI: -0·18,0·15), P=0·82; metabolism score: SBP, beta -0·16 mmHg (95%CI: -0·45,0·14), P=0·29 and DBP, beta 0·05 mmHg (95%CI: -0·15,0·24), P=0·65].

II. References

1. Power C, Elliott J. Cohort profile: 1958 British birth cohort (National Child Development Study). Int J Epidemiol 2006;35:34-41.
2. Hyppönen E, Turner S, Cumberland P, Power C, Gibb I. Serum 25-hydroxyvitamin D measurement in a large population survey with statistical harmonization of assay variation to an international standard. The Journal of clinical endocrinology and metabolism 2007;92:4615-22.
3. Craddock N, Hurles ME, Cardin N, et al. Genome-wide association study of CNVs in 16,000 cases of eight common diseases and 3,000 shared controls. Nature 2010;464:713-20.
4. Todd JA, Walker NM, Cooper JD, et al. Robust associations of four new chromosome regions from genome-wide analyses of type 1 diabetes. Nat Genet 2007;39:857-64.
5. Mosekilde L, Hermann AP, Beck-Nielsen H, Charles P, Nielsen SP, Sorensen OH. The Danish Osteoporosis Prevention Study (DOPS): project design and inclusion of 2000 normal perimenopausal women. Maturitas 1999;31:207-19.
6. Knowler WC, Barrett-Connor E, Fowler SE, et al. Reduction in the incidence of type 2 diabetes with lifestyle intervention or metformin. The New England journal of medicine 2002;346:393-403.
7. Fowkes FG, Housley E, Cawood EH, Macintyre CC, Ruckley CV, Prescott RJ. Edinburgh Artery Study: prevalence of asymptomatic and symptomatic peripheral arterial disease in the general population. International journal of epidemiology 1991;20:384-92.
8. Schottker B, Jansen EH, Haug U, Schomburg L, Kohrle J, Brenner H. Standardization of misleading immunoassay based 25-hydroxyvitamin D levels with liquid chromatography tandem-mass spectrometry in a large cohort study. PloS one 2012;7:e48774.
9. Schottker B, Raum E, Rothenbacher D, Muller H, Brenner H. Prognostic value of haemoglobin A1c and fasting plasma glucose for incident diabetes and implications for screening. European journal of epidemiology 2011;26:779-87.
10. Jaquish CE. The Framingham Heart Study, on its way to becoming the gold standard for Cardiovascular Genetic Epidemiology? BMC Med Genet 2007;8:63.
11. Lorentzon M, Swanson C, Andersson N, Mellstrom D, Ohlsson C. Free testosterone is a positive, whereas free estradiol is a negative, predictor of cortical bone size in young Swedish men: the GOOD study. J Bone Miner Res 2005;20:1334-41.
12. Shea MK, Houston DK, Tooze JA, et al. Correlates and prevalence of insufficient 25-hydroxyvitamin D status in black and white older adults: the health, aging and body composition study. J Am Geriatr Soc 2011;59:1165-74.
13. Barker DJ, Osmond C, Forsen TJ, Kajantie E, Eriksson JG. Trajectories of growth among children who have coronary events as adults. The New England journal of medicine 2005;353:1802-9.
14. Dennison EM, Syddall HE, Aihie Sayer A, Martin HJ, Cooper C. Lipid profile, obesity and bone mineral density: the Hertfordshire Cohort Study. Qjm 2007;100:297-303.
15. Melzer D, Perry JR, Hernandez D, et al. A genome-wide association study identifies protein quantitative trait loci (pQTLs). PLoS genetics 2008;4:e1000072.
16. Zutavern A, Brockow I, Schaaf B, et al. Timing of solid food introduction in relation to atopic dermatitis and atopic sensitization: results from a prospective birth cohort study. Pediatrics 2006;117:401-11.
17. Winkelmann BR, Marz W, Boehm BO, et al. Rationale and design of the LURIC study--a resource for functional genomics, pharmacogenomics and long-term prognosis of cardiovascular disease. Pharmacogenomics 2001;2:S1-73.
18. Kring SI, Larsen LH, Holst C, et al. Genotype-phenotype associations in obesity dependent on definition of the obesity phenotype. Obesity facts 2008;1:138-45.
19. Kring SI, Holst C, Zimmermann E, et al. FTO gene associated fatness in relation to body fat distribution and metabolic traits throughout a broad range of fatness. PloS one 2008;3:e2958.
20. Paternoster L, Evans DM, Nohr EA, et al. Genome-wide population-based association study of extremely overweight young adults--the GOYA study. PloS one 2011;6:e24303.
21. Li Y, Willer CJ, Ding J, Scheet P, Abecasis GR. MaCH: using sequence and genotype data to estimate haplotypes and unobserved genotypes. Genetic epidemiology 2010;34:816-34.
22. Wadsworth M, Kuh D, Richards M, Hardy R. Cohort Profile: The 1946 National Birth Cohort (MRC National Survey of Health and Development). International journal of epidemiology 2006;35:49-54.
23. Penninx BW, Beekman AT, Smit JH, et al. The Netherlands Study of Depression and Anxiety (NESDA): rationale, objectives and methods. International journal of methods in psychiatric research 2008;17:121-40.
24. Sullivan PF, de Geus EJ, Willemsen G, et al. Genome-wide association for major depressive disorder: a possible role for the presynaptic protein piccolo. Molecular psychiatry 2009;14:359-75.

25. Järvelin MR, Sovio U, King V, et al. Early life factors and blood pressure at age 31 years in the 1966 northern Finland birth cohort. Hypertension 2004;44:838-46.
26. Mellstrom D, Johnell O, Ljunggren O, et al. Free testosterone is an independent predictor of BMD and prevalent fractures in elderly men: MrOS Sweden. Journal of bone and mineral research : the official journal of the American Society for Bone and Mineral Research 2006;21:529-35.
27. Hillege HL, Fidler V, Diercks GF, et al. Urinary albumin excretion predicts cardiovascular and noncardiovascular mortality in general population. Circulation 2002;106:1777-82.
28. Lind L, Fors N, Hall J, Marttala K, Stenborg A. A comparison of three different methods to evaluate endothelium-dependent vasodilation in the elderly: the Prospective Investigation of the Vasculature in Uppsala Seniors (PIVUS) study. Arterioscler Thromb Vasc Biol 2005;25:2368-75.
29. Fan JB, Oliphant A, Shen R, et al. Highly parallel SNP genotyping. Cold Spring Harb Symp Quant Biol 2003;68:69-78.
30. Miller GJ, Bauer KA, Barzegar S, Cooper JA, Rosenberg RD. Increased activation of the haemostatic system in men at high risk of fatal coronary heart disease. Thrombosis and haemostasis 1996;75:767-71.
31. Huisman M, Oldehinkel AJ, de Winter A, et al. Cohort profile: the Dutch 'TRacking Adolescents' Individual Lives' Survey'; TRAILS. International journal of epidemiology 2008;37:1227-35.
32. Jorde R, Schirmer H, Wilsgaard T, et al. Polymorphisms related to the serum 25-hydroxyvitamin D level and risk of myocardial infarction, diabetes, cancer and mortality. The Tromso Study. PloS one 2012;7:e37295.
33. Spector TD, Williams FM. The UK Adult Twin Registry (TwinsUK). Twin Res Hum Genet 2006;9:899-906.
34. Michaelsson K, Baron JA, Snellman G, et al. Plasma vitamin D and mortality in older men: a community-based prospective cohort study. Am J Clin Nutr 2010;92:841-8.
35. Kivimaki M, Magnussen CG, Juonala M, et al. Conventional and Mendelian randomization analyses suggest no association between lipoprotein(a) and early atherosclerosis: the Young Finns Study. Int J Epidemiol 2011;40:470-8.
36. Raitakari OT, Juonala M, Ronnemaa T, et al. Cohort profile: the cardiovascular risk in Young Finns Study. Int J Epidemiol 2008;37:1220-6.
37. Ehret GB, Munroe PB, Rice KM, et al. Genetic variants in novel pathways influence blood pressure and cardiovascular disease risk. Nature 2011;478:103-9.
38. Levy D, Ehret GB, Rice K, et al. Genome-wide association study of blood pressure and hypertension. Nature genetics 2009;41:677-87.
39. Newton-Cheh C, Johnson T, Gateva V, et al. Genome-wide association study identifies eight loci associated with blood pressure. Nature genetics 2009;41:666-76.

IV. Supplementary tables

Supplementary table 1: Effect allele frequencies and genotyping information for the four vitamin D-related single nucleotide polymorphisms (SNPs)

Study	*DHCR7 (rs12785878, effect allele=G)*		*CYP2R1 (rs12794714, effect allele=A)*		*GC (rs2282679, effect allele=G*		*CYP24A1 (rs6013897, effect allele=A)*	
	**Genotyping**	**EAF, %**	**Genotyping**	**EAF, %**	**Genotyping**	**EAF, %**	**Genotyping**	**EAF, %**
1958BC	Genotyped	22	Genotyped	43	Genotyped	30	Genotyped	20
CROATIA-Korcula	Genotyped	34	Imputed	44	Imputed	30	Genotyped	18
CROATIA-Split	Genotyped	33	Imputed	47	Imputed	27	Genotyped	20
CROATIA-Vis	Genotyped	31	Imputed	48	Imputed	28	Genotyped	17
DOPS	Genotyped	31	Genotyped	40	Genotyped	27	Genotyped	20
DPP¥	Genotyped	25	n/a	-	Genotyped	28	Genotyped	21
EAS	Genotyped	19	Genotyped	40	Genotyped	29	Genotyped	20
ELSA	Genotyped	23	Genotyped	43	Genotyped	29	Genotyped	19
ESTHER*	Genotyped	26	Genotyped	46	n/a	-	n/a	-
FHS	Genotyped	26	Genotyped	43	Genotyped	28	Genotyped	21
GENMETS	Imputed	38	Genotyped	41	Genotyped	19	Imputed	23
GOOD	Genotyped	33	Imputed	42	Genotyped	25	Genotyped	21
HBCS	Imputed	38	Imputed	39	Imputed	20	Imputed	23
HCS	Genotyped	26	Genotyped	44	Genotyped	30	Genotyped	20
Health ABC	Imputed	26	Imputed	45	Imputed	29	Imputed	21
InChianti	Genotyped	25	Genotyped	48	Genotyped	25	Genotyped	24
LifeLines	Imputed	25	Imputed	42	Imputed	27	Imputed	13
LISAplus & GINIplus	Genotyped	28	Imputed	43	Genotyped	27	Genotyped	20
LURIC	Imputed	24	Imputed	45	Genotyped	28	Imputed	21
GOYA	Genotyped	30	Genotyped	40	Genotyped	28	Genotyped	20
MRC NSHD	Genotyped	22	Genotyped	42	Genotyped	28	Genotyped	20
MrOS Sweden	Genotyped	32	Imputed	42	Genotyped	26	Genotyped	20
NESDA	Imputed	27	Imputed	44	Imputed	28	Imputed	19
NFBC 1966	Imputed	39	Imputed	39	Imputed	20	Imputed	23
NFBC 1986	Genotyped	40	Genotyped	39	Genotyped	19	Genotyped	22
NPHSII	Genotyped	22	Genotyped	42	Genotyped	29	Genotyped	19
ORCADES	Genotyped	31	Imputed	38	Imputed	26	Genotyped	22
PIVUS	Genotyped	35	Genotyped	40	Genotyped	26	Genotyped	21
PREVEND*	Imputed	25	Imputed	44	Imputed	27	n/a	-
TRAILS	Imputed	28	Imputed	41	Genotyped	27	Imputed	14
Tromsø	Genotyped	39	Genotyped	41	Genotyped	25	Genotyped	23
Twins UK	Genotyped	23	Genotyped	42	Genotyped	30	Genotyped	20
ULSAM	Genotyped	33	Genotyped	39	Genotyped	26	Genotyped	22
Whitehall II	Genotyped	23	Genotyped	42	Genotyped	29	Genotyped	20
Young Finns	Genotyped	40	Genotyped	38	Genotyped	19	Genotyped	24
Studies with SNP imputed (% of total)	9 (25)	-	15 (43)	-	10 (29)	-	8 (23)	-

Max N with SNP imputed (% of total N)	31,815 (29)	-	38,599 (36)	-	29,270 (27)	-	28,166 (26)	-

DHCR7, 7-dehydrocholesterol reductase; *CYP2R1*, 25-hydroxylase; *GC*, Vitamin D binding protein; *CYP24A1*, 25-hydroxyvitamin D 24-hydroxylase; EAF, effect allele frequency

***** ESTHER and PREVEND studies were not included in the metabolism allele score, as the SNPs required for creating the allele score were not available.

[¥] The DPP study was not included in the synthesis allele score, as the *CYP2R1* SNP required for creating the allele score was not available.

Supplementary table 2: Effect allele frequencies of the four vitamin D- related single nucleotide polymorphisms (SNPs), mean age, mean body mass index (BMI) and 25-hydroxyvitamin D levels, stratified by geographical region (31 D-CarDia studies in adults)

Geographical Region	Countries in region	Studies in region	Studies with 25(OH)D	*DHCR7* EAF: mean (sd)	*CYP2R1* EAF: mean (sd)	*GC* EAF: mean (sd)	*CYP24A1* EAF: mean (sd)	Average study age: mean (sd)	Average study BMI: mean (sd)	Average study SBP: mean (sd)	Average study DBP: mean (sd)	% hyper-tensive in study: mean (sd)	Average study ln-25(OH)D: mean (sd)
Great Britain	GB	58BC, EAS, ELSA, HCS, NPHSII, MRC NSHD, ORKNEY, Twins UK, WHII	58BC, HCS, Twins UK	0.23 (0.03)	0.42 (0.02)	0.29 (0.01)	0.20 (0.01)	57.13 (7.67)	26.87 (0.89)	134.74 (6.51)	79.08 (3.95)	42.99 (11.26)	3.97 (0.25)
Central and South Europe	Croatia, Italy, Germany, Netherlands	ESTHER, InChianti, KORCULA, Lifelines, NESDA, LURIC, PREVEND, SPLIT, VIS	ESTHER, InChianti, LURIC	0.28 (0.04)	0.45 (0.02)	0.28 (0.01)	0.19 (0.03)	54.99 (8.49)	26.96 (0.78)	140.06 (8.5)	84.96 (6.36)	51.54 (20.79)	3.74 (0.11)
Finland	Finland	GENMETS, HBCS, NFBC66, Young Finns	GENMETS, NFBC66, Young Finns	0.39 (0.01)	0.39 (0.01)	0.20 (0.01)	0.23 (0.00)	45.18 (13.60)	25.89 (1.30)	132.49 (12.88)	82.23 (7.18)	37.43 (26.04)	3.99 (0.18)
Scandanavia	Denmark, Norway, Sweden	DOPS, GOYA, MrOS, PIVUS, TROMSO, ULSAM	DOPS, MrOS, PIVUS, TROMSO, ULSAM	0.33 (0.03)	0.40 (0.01)	0.26 (0.01)	0.21 (0.01)	61.98 (12.2)	26.85 (1.85)	147.06 (8.70)	85.34 (4.25)	59.78 (12.13)	4.08 (0.10)
North America	USA	DPP, FHS, Health ABC	DPP, FHS, Health ABC	0.26 (0.01)	0.44 (0.01)	0.28 (0.01)	0.21 (0.00)	57.74 (15.01)	29.29 (4.18)	129.4 (9.12)	76.75 (2.89)	39.51 (21.84)	3.54 (0.40)

EAF, Effect allele frequency; SBP, systolic blood pressure; DBP, diastolic blood pressure; sd, standard deviation

Supplementary table 3: Mutually adjusted association of log transformed 25(OH)D with confounding variables

Covariate	Study	Beta (SE)	P-value
**Age**(i)	DOPS	-0.023 (0.143)	0.875
	ESTHER	0.026 (0.012)	0.026
	HCS	-0.098 (0.240)	0.683
	LURIC	0.044 (0.007)	<0.0001
	MrOS	0.072 (0.144)	0.616
	PIVUS	0.082 (0.055)	0.135
	TROMSO	0.025 (0.007)	0.0002
	ULSAM	0.017 (0.015)	0.251
Meta-analysis:	I^2 *= 0 %*	*0.032 (0.004)*	*<0.0001*
**Age-squared**(ii)	DOPS	0.0003 (0.001)	0.832
	ESTHER	-0.0002 (0.0001)	0.008
	HCS	0.001 (0.002)	0.688
	LURIC	-0.0004 (0.0001)	<0.0001
	MrOS	-0.0004 (0.001)	0.646
	TROMSO	-0.0002 (0.0001)	<0.0001
Meta-analysis:	I^2 *= 18 %*	*-0.0003 (0.00004)*	*<0.0001*
**Sex**	1958BC	-0.069 (0.011)	<0.0001
	ESTHER	-0.164 (0.009)	<0.0001
	HCS	-0.173 (0.028)	<0.0001
	LURIC	-0.175 (0.020)	<0.0001
	NFBC66	-0.011 (0.008)	0.130
	PIVUS	-0.080 (0.023)	0.0005
	TROMSO	-0.065 (0.009)	<0.0001
Meta-analysis:	I^2 *= 97 %*	*-0.104 (0.027)*	*<0.0001*
**Season**(iii)	1958BC	-0.387 (0.011)	<0.0001
	DOPS	-0.410 (0.027)	<0.0001
	ESTHER	-0.221 (0.009)	<0.0001

	HCS	-0.389 (0.026)	<0.0001
	LURIC	-0.417 (0.018)	<0.0001
	MrOS	-0.152 (0.019)	<0.0001
	NFBC66	-0.079 (0.010)	<0.0001
	PIVUS	-0.153 (0.022)	<0.0001
	TROMSO	-0.099 (0.009)	<0.0001
	ULSAM	-0.118 (0.019)	<0.0001
Meta-analysis:	*I^2 = 99 %*	*-0.242 (0.042)*	<0.0001
**BMI**	1958BC	-0.014 (0.001)	<0.0001
	DOPS	-0.011 (0.003)	0.001
	ESTHER	-0.007 (0.001)	<0.0001
	HCS	-0.004 (0.003)	0.158
	LURIC	-0.008 (0.002)	0.0002
	MrOS	-0.010 (0.003)	0.0002
	NFBC66	-0.005 (0.001)	<0.0001
	PIVUS	-0.013 (0.003)	<0.0001
	TROMSO	-0.009 (0.001)	<0.0001
	ULSAM	-0.004 (0.003)	0.152
Meta-analysis:	*I^2 = 81 %*	*-0.009 (0.001)*	<0.0001
**Total Cholesterol**	1958BC	-0.016 (0.005)	0.003
	DOPS	0.010 (0.013)	0.474
	ESTHER	0.001 (0.0001)	<0.0001
	HCS	-0.007 (0.012)	0.575
	LURIC	0.001 (0.0002)	0.0001
	MrOS	0.034 (0.009)	0.0002
	NFBC66	0.018 (0.004)	0.0002
	PIVUS	-0.057 (0.011)	<0.0001
	TROMSO	0.013 (0.004)	0.001
	ULSAM	0.010 (0.009)	0.302
Meta-analysis:	*I^2 = 89 %*	*0.002 (0.001)*	*0.052*

**Triglycerides**	1958BC	-0.016 (0.004)	<0.0001
	DOPS	-0.051 (0.022)	0.023
	ESTHER	-0.0003 (0.00005)	<0.0001
	HCS	-0.060 (0.018)	0.0006
	LURIC	-0.0003 (0.0001)	0.0002
	MrOS	-0.049 (0.013)	0.0002
	NFBC66	0.010 (0.006)	0.091
	PIVUS	-0.082 (0.018)	<0.0001
	TROMSO	-0.039 (0.005)	<0.0001
	ULSAM	0.018 (0.013)	0.163
Meta-analysis:	*I^2 = 93 %*	*-0.001 (0.001)*	*0.03*

(i) Main effect in model with squared term (uncentered)

(ii) Squared term for age was not included for PIVUS or ULSAM studies, since the age range in the analysis sample for these studies was very small (range 69-71 and 59-73 years respectively)

(iii) In main analyses, models were adjusted for month; for these analyses a binary variable for season [winter/spring vs. summer/autumn] was used in order to present a single effect estimate for each study

Random effects meta-analyses were used in the presence of heterogeneity (i.e. for all models except age/age^2)

Supplementary table 4: Association of 25(OH)D increasing alleles in *CYP2R1*, *DHCR7* and Synthesis score with potential confounding factors as outcomes

		CYP2R1		***DHCR7***		**Synthesis Score**	
**Outcome**	**Study**	Beta (SE)	P-value	Beta (SE)	P-value	Beta (SE)	P-value
Age	DOPS	-0.026 (0.102)	0.799	0.086 (0.108)	0.429	0.018 (0.078)	0.817
	EAS	0.100 (0.290)	0.732	0.097 (0.343)	0.778	0.131 (0.232)	0.572
	ELSA	0.141 (0.194)	0.467	0.176 (0.225)	0.435	0.181 (0.154)	0.242
	ESTHER	0.184 (0.104)	0.077	0.209 (0.119)	0.079	0.206 (0.081)	0.011
	HBCS	-0.076 (0.103)	0.463	-0.076 (0.104)	0.465	-0.074 (0.078)	0.338
	HCS	-0.042 (0.076)	0.584	0.104 (0.116)	0.370	0.001 (0.081)	0.994
	LURIC	-0.070 (0.277)	0.801	0.542 (0.324)	0.095	0.201 (0.226)	0.374
	MrOS	-0.117 (0.154)	0.447	0.022 (0.090)	0.807	0.074 (0.118)	0.531
	NPHSII	0.089 (0.099)	0.367	0.073 (0.112)	0.511	0.074 (0.078)	0.345
	PIVUS	-0.005 (0.010)	0.619	-0.005 (0.010)	0.583	-0.005 (0.008)	0.484
	TROMSO	0.182 (0.204)	0.373	-0.190 (0.203)	0.348	-0.027 (0.153)	0.861
	ULSAM	-0.009 (0.028)	0.735	-0.008 (0.028)	0.781	-0.004 (0.021)	0.848
	WHII	0.041 (0.127)	0.748	0.070 (0.150)	0.641	0.031 (0.101)	0.756
	**Meta-analysis**	**-0.004 (0.009)**	**0.671**	**-0.002 (0.009)**	**0.804**	**-0.002 (0.007)**	**0.751**
		**I^2 = 0**	**P_{het} = 0.843**	**I^2 = 0**	**P_{het} = 0.577**	**I^2 = 0**	**P_{het} = 0.459**
ln BMI	1958BC	0.003 (0.003)	0.315	-0.001 (0.004)	0.681	0.002 (0.002)	0.478
	DOPS	0.013 (0.006)	0.021	-0.004 (0.006)	0.518	0.006 (0.004)	0.219
	EAS	-0.002 (0.007)	0.743	0.004 (0.009)	0.623	-0.000 (0.006)	0.951
	ELSA	-0.002 (0.003)	0.639	0.002 (0.004)	0.678	-0.001 (0.003)	0.839
	ESTHER	-0.005 (0.002)	0.044	-0.000 (0.003)	0.899	-0.002 (0.002)	0.319
	HBCS	-0.007 (0.006)	0.209	-0.002 (0.006)	0.696	-0.005 (0.004)	0.226
	HCS	0.004 (0.004)	0.355	-0.004 (0.007)	0.585	-0.001 (0.005)	0.813
	LURIC	0.003 (0.004)	0.415	-0.003 (0.004)	0.436	-0.001 (0.003)	0.695
	MrOS	-0.003 (0.006)	0.591	-0.003 (0.004)	0.427	-0.005 (0.005)	0.252
	NFBC66	-0.003 (0.003)	0.312	-0.003 (0.003)	0.419	-0.003 (0.003)	0.186
	NPHSII	-0.001 (0.004)	0.824	0.011 (0.004)	0.009	0.005 (0.003)	0.1
	PIVUS	-0.004 (0.008)	0.64	0.010 (0.007)	0.15	0.003 (0.006)	0.651

	TROMSO	0.004 (0.002)	0.112	-0.000 (0.002)	0.962	0.001 (0.002)	0.446
	ULSAM	-0.013 (0.005)	0.022	-0.007 (0.006)	0.194	-0.009 (0.004)	0.026
	WHII	0.006 (0.003)	0.076	0.007 (0.004)	0.06	0.007 (0.003)	0.012
	**Meta-analysis**	**0.000 (0.001)**	**0.975**	**0.000 (0.001)**	**0.855**	**-0.000 (0.001)**	**0.99**
		**I^2 = 47**	**P_{het} = 0.024**	**I^2 = 20**	**P_{het} = 0.233**	**I^2 = 38**	**P_{het} = 0.069**
Total Cholesterol	1958BC	-0.004 (0.020)	0.841	0.022 (0.023)	0.336	0.007 (0.016)	0.676
	DOPS	0.028 (0.044)	0.526	0.029 (0.047)	0.533	0.038 (0.034)	0.262
	EAS	-0.029 (0.068)	0.669	0.122 (0.081)	0.131	0.047 (0.054)	0.384
	ELSA	0.010 (0.024)	0.679	0.002 (0.028)	0.943	0.007 (0.019)	0.716
	ESTHER	0.652 (0.825)	0.43	1.138 (0.944)	0.228	0.695 (0.639)	0.277
	HBCS	-0.030 (0.037)	0.42	-0.005 (0.038)	0.896	-0.018 (0.028)	0.53
	HCS	0.034 (0.032)	0.294	0.055 (0.053)	0.296	0.064 (0.037)	0.086
	LURIC	-1.894 (1.142)	0.097	0.584 (1.326)	0.66	-1.098 (0.927)	0.236
	MrOS	0.009 (0.050)	0.852	-0.018 (0.051)	0.718	-0.014 (0.038)	0.706
	NFBC66	0.028 (0.021)	0.185	0.010 (0.021)	0.618	0.024 (0.015)	0.118
	NPHSII	0.073 (0.029)	0.013	0.039 (0.033)	0.239	0.057 (0.023)	0.015
	PIVUS	-0.042 (0.048)	0.384	-0.009 (0.047)	0.85	-0.022 (0.036)	0.541
	TROMSO	0.019 (0.020)	0.345	-0.031 (0.020)	0.111	-0.011 (0.015)	0.447
	ULSAM	-0.019 (0.043)	0.659	-0.019 (0.044)	0.67	-0.016 (0.032)	0.609
	WHII	0.027 (0.022)	0.212	0.032 (0.026)	0.206	0.031 (0.017)	0.073
	**Meta-analysis**	**0.016 (0.008)**	**0.045**	**0.007 (0.009)**	**0.383**	**0.013 (0.006)**	**0.039**
		**I^2 = 0**	**P_{het} = 0.503**	**I^2 = 0**	**P_{het} = 0.628**	**I^2 = 18**	**P_{het} = 0.258**
ln Triglycerides	1958BC	-0.005 (0.011)	0.617	-0.005 (0.013)	0.718	-0.004 (0.009)	0.658
	DOPS	0.009 (0.017)	0.59	-0.005 (0.019)	0.783	0.003 (0.013)	0.814
	EAS	-0.020 (0.023)	0.384	-0.002 (0.027)	0.949	-0.014 (0.019)	0.467
	ELSA	-0.011 (0.010)	0.284	-0.029 (0.012)	0.015	-0.019 (0.008)	0.019
	ESTHER	-0.011 (0.009)	0.219	0.007 (0.010)	0.526	-0.003 (0.007)	0.638
	HBCS	-0.025 (0.016)	0.131	-0.040 (0.016)	0.017	-0.035 (0.012)	0.004
	HCS	0.020 (0.013)	0.111	0.016 (0.021)	0.465	0.013 (0.015)	0.376
	LURIC	-0.010 (0.013)	0.435	0.009 (0.015)	0.565	-0.005 (0.010)	0.638

	MrOS	0.003 (0.021)	0.879	-0.000 (0.021)	0.991	0.001 (0.016)	0.946
	NFBC66	0.004 (0.011)	0.73	0.003 (0.010)	0.798	0.005 (0.008)	0.535
	NPHSII	0.008 (0.015)	0.586	0.017 (0.017)	0.329	0.012 (0.012)	0.327
	PIVUS	0.009 (0.021)	0.669	0.010 (0.020)	0.623	0.009 (0.016)	0.565
	TROMSO	-0.001 (0.008)	0.927	-0.013 (0.008)	0.107	-0.009 (0.006)	0.162
	ULSAM	-0.020 (0.020)	0.325	-0.023 (0.021)	0.274	-0.019 (0.015)	0.207
	WHII	-0.000 (0.011)	0.968	0.015 (0.013)	0.242	0.006 (0.009)	0.459
	**Meta-analysis**	**-0.003 (0.003)**	**0.351**	**-0.004 (0.004)**	**0.259**	**-0.004 (0.003)**	**0.088**
		**I^2 = 0**	**P_{het} = 0.753**	**I^2 = 24**	**P_{het} = 0.193**	**I^2 = 25**	**P_{het} = 0.177**
Sex	1958BC	-0.063 (0.036)	0.08	0.078 (0.041)	0.058	-0.006 (0.028)	0.844
	EAS	0.081 (0.103)	0.434	-0.023 (0.122)	0.852	0.047 (0.082)	0.565
	ELSA	0.004 (0.040)	0.912	0.003 (0.047)	0.946	-0.001 (0.032)	0.97
	ESTHER	0.018 (0.032)	0.566	-0.024 (0.036)	0.513	-0.001 (0.025)	0.958
	HBCS	0.043 (0.071)	0.549	0.146 (0.072)	0.042	0.107 (0.054)	0.046
	HCS	0.045 (0.054)	0.398	0.170 (0.089)	0.056	0.035 (0.062)	0.574
	LURIC	-0.078 (0.057)	0.168	0.004 (0.066)	0.954		
	NFBC66	-0.028 (0.043)	0.521	0.018 (0.043)	0.667	-0.008 (0.032)	0.792
	PIVUS	0.096 (0.096)	0.319	-0.035 (0.093)	0.704	0.039 (0.072)	0.587
	TROMSO	-0.026 (0.030)	0.394	-0.009 (0.030)	0.772	-0.012 (0.022)	0.599
	WHII	-0.047 (0.050)	0.346	0.017 (0.059)	0.771	-0.034 (0.039)	0.39
	**Meta-analysis**	**-0.013 (0.013)**	**0.329**	**0.018 (0.015)**	**0.233**	**-0.003 (0.011)**	**0.808**
		**I^2 = 0**	**P_{het} = 0.527**	**I^2 = 8**	**P_{het} = 0.365**	**I^2 = 0**	**P_{het} = 0.739**
Season**	1958BC	-0.028 (0.038)	0.461	-0.065 (0.042)	0.126	-0.047 (0.030)	0.113
	DOPS	-0.091 (0.072)	0.206	0.058 (0.077)	0.45	-0.035 (0.056)	0.533
	ESTHER	0.002 (0.031)	0.958	-0.018 (0.036)	0.616	-0.009 (0.024)	0.702
	HCS	0.015 (0.053)	0.775	-0.109 (0.089)	0.224	-0.046 (0.062)	0.463
	LURIC	-0.051 (0.052)	0.331	0.062 (0.061)	0.312	-0.014 (0.043)	0.734
	MrOS	-0.040 (0.097)	0.677	0.124 (0.101)	0.216	0.057 (0.074)	0.437
	NFBC66	-0.011 (0.047)	0.815	0.067 (0.046)	0.149	0.030 (0.034)	0.382
	PIVUS	0.035 (0.097)	0.715	0.144 (0.094)	0.125	0.122 (0.073)	0.095

	TROMSO	0.050 (0.043)	0.251	0.089 (0.043)	0.039	0.078 (0.033)	0.016
	ULSAM	-0.058 (0.087)	0.508	0.064 (0.089)	0.468	-0.007 (0.065)	0.91
	**Meta-analysis**	**-0.009 (0.016)**	**0.575**	**0.031 (0.024)**	**0.197**	**0.007 (0.017)**	**0.672**
		**I^2 = 0**	**P_{het} = 0.836**	**I^2 = 38**	**P_{het} = 0.105**	**I^2 = 33**	**P_{het} = 0.142**
Current smoking	1958BC	0.042 (0.042)	0.322	-0.027 (0.048)	0.57	0.020 (0.033)	0.557
	EAS	0.166 (0.126)	0.188	-0.199 (0.144)	0.168	0.012 (0.099)	0.905
	ELSA	0.066 (0.059)	0.261	-0.056 (0.067)	0.404	0.012 (0.047)	0.804
	ESTHER	-0.086 (0.042)	0.042	-0.000 (0.049)	0.998	-0.054 (0.033)	0.098
	HBCS	-0.003 (0.081)	0.97	0.009 (0.082)	0.909	0.024 (0.061)	0.692
	HCS	-0.036 (0.081)	0.656	-0.030 (0.127)	0.813	0.000 (0.089)	0.998
	LURIC	0.072 (0.062)	0.249	-0.036 (0.072)	0.614	0.029 (0.051)	0.571
	MrOS	0.183 (0.180)	0.312	-0.019 (0.184)	0.919	0.096 (0.136)	0.478
	NFBC66	-0.096 (0.046)	0.036	-0.022 (0.045)	0.626	-0.070 (0.033)	0.036
	NPHSII	0.084 (0.064)	0.187	0.069 (0.073)	0.343	0.095 (0.051)	0.061
	PIVUS	0.511 (0.166)	0.002	-0.143 (0.147)	0.332	0.204 (0.117)	0.082
	TROMSO	-0.011 (0.032)	0.719	0.006 (0.031)	0.86	-0.003 (0.024)	0.893
	ULSAM	0.054 (0.108)	0.617	-0.095 (0.109)	0.384	-0.021 (0.081)	0.796
	WHII	-0.035 (0.077)	0.649	0.248 (0.098)	0.011	0.073 (0.062)	0.237
	**Meta-analysis**	**0.018 (0.025)**	**0.473**	**-0.007 (0.017)**	**0.701**	**-0.001 (0.012)**	**0.96**
		**I^2 = 52**	**P_{het} = 0.013**	**I^2 = 0**	**P_{het} = 0.491**	**I^2 = 21**	**P_{het} = 0.220**

P_{het}: heterogeneity p-value

Estimates are from linear regression for continuous outcomes (age, BMI, total Cholesterol, triglycerides) and logistic regression for binary outcomes (sex, season [winter/spring vs. summer/autumn] and current smoking). Random effects models were used for all meta-analyses indicating high levels of heterogeneity (judged by I-squared > 30%), otherwise fixed effects models were used.

Supplementary table 5: Association of the vitamin D-related SNPs and allele scores with 25-hydroxyvitamin D concentrations (D-CarDia, N up to 49,346 adults and 1,776 adolescents).

Instrument	Studies included	Coefficient (95% CI)	MA P-value	Heterogeneity P-value	I-squared, %	MA model
DHCR7	Adults	3.29 (2.41, 4.17)	<0.0001	0.0006	60.6	Random
	Adolescents	4.06 (2.08, 6.05)	<0.0001	0.89	0	Fixed
CYP2R1	Adults	2.58 (2.12, 3.05)	<0.0001	0.21	21.2	Fixed
	Adolescents	3.38 (1.52, 5.23)	0.0004	0.26	22.1	Fixed
Synthesis score	Adults	2.83 (2.48, 3.19)	<0.0001	0.48	0	Fixed
	Adolescents	3.83 (2.43, 5.22)	<0.0001	0.31	2.2	Fixed
GC	Adults	8.54 (7.43, 9.65)	<0.0001	<0.0001	67.8	Random
	Adolescents	9.51 (2.26, 16.75)	0.01	0.0003	92.2	Random
CYP24A1	Adults	2.1 (1.51, 2.68)	<0.0001	0.86	0	Fixed
	Adolescents	3.77 (1.53, 6.01)	0.0001	0.97	0	Fixed
Metabolism score	Adults	5.38 (4.67, 6.08)	<0.0001	0.003	57.0	Random
	Adolescents	6.99 (2.84, 11.14)	0.0001	0.01	86.5	Random

Supplementary table 6: Association between *DHCR7*, *CYP2R1* and the synthesis score with blood pressure and hypertension, with and without adjustment for serum lipids. Subsample of D-CarDia cohorts with full information on *DHCR7*, *CYP2R1*, blood pressures and lipids (29 studies, n=81,905).*

		Adjustment*	Coefficient	SE	I^2 %	MA model
Systolic BP	*DHCR7*	Basic	-0.075	0.140	44.9	Random
		+ Lipids	-0.083	0.136	41.9	Random
	CYP2R1	Basic	-0.071	0.084	0	Fixed
		+ Lipids	-0.054	0.084	0	Fixed
	Synthesis score	Basic	-0.114	0.079	20.3	Fixed
		+ Lipids	-0.108	0.064	22.4	Fixed
Diastolic BP	*DHCR7*	Basic	-0.103	0.085	44.7	Random
		+ Lipids	-0.113	0.084	43.5	Random
	CYP2R1	Basic	-0.089	0.051	3.4	Fixed
		+ Lipids	-0.075	0.051	5.3	Fixed
	Synthesis score	Basic	-0.103	0.053	30.6	Random
		+ Lipids	-0.102	0.055	35.4	Random
Hypertension	*DHCR7*	Basic	-0.008	0.019	51.2	Random
		+ Lipids	-0.014	0.018	41.6	Random
	CYP2R1	Basic	-0.014	0.011	0	Fixed
		+ Lipids	-0.010	0.011	0	Fixed
	Synthesis score	Basic	-0.016	0.009	1.5	Fixed
		+ Lipids	-0.013	0.009	0	Fixed

*Two studies excluded, which did not have information on lipids.

Supplementary table 7: Associations between *DHCR7* and *CYP2R1* with 25(OH)D, SBP, DBP and Hypertension for genotyped vs. imputed SNPs in 31 adult D-CarDia cohorts (N up to 99,582).

		Genotyping	# studies	total N	Coefficient	SE	I^2 %	MA model
**25(OH)D**	*DHCR7*	Genotyped	13	38,829	0.034	0.005	59.6	Random
		Imputed	4	9,650	0.029	0.009	54.5	Random
	CYP2R1	Genotyped	12	35,211	0.025	0.003	23.0	Fixed
		Imputed	4	9,784	0.029	0.004	24.0	Fixed
**Systolic BP**	*DHCR7*	Genotyped	23	63,693	-0.154	0.177	47.7	Random
		Imputed	8	30,122	0.073	0.184	19.4	Random
	CYP2R1	Genotyped	18	59,455	-0.058	0.107	0.0	Fixed
		Imputed	12	33,120	-0.046	0.131	0.0	Fixed
**Diastolic BP**	*DHCR7*	Genotyped	23	63,689	-0.170	0.095	35.8	Random
		Imputed	8	30,117	0.062	0.156	51.6	Random
	CYP2R1	Genotyped	18	59,451	-0.088	0.065	11.8	Fixed
		Imputed	12	33,115	-0.077	0.081	0.0	Fixed
**Hypertension**	*DHCR7*	Genotyped	23	65,724	-0.017	0.022	47.9	Random
		Imputed	8	30,118	0.012	0.040	57.1	Random
	CYP2R1	Genotyped	18	59,599	-0.011	0.013	0.0	Fixed
		Imputed	12	33,150	-0.019	0.020	0.0	Fixed

Supplementary table 8: Comparison of adjustments to blood pressure values for individuals taking medication, with 15 mmHg added to SBP and 10mmHg to DBP being compared to 10mmHg to SBP [*SBP (ii) in table*] and 5 mmHg to DBP [DBP (ii) in table]. Subsample of D-CarDia cohorts with individual level data and full information on *DHCR7*, *CYP2R1*, blood pressures and 25(OH)D (10 studies, n up to 34,609).

Exposure	Outcome	Coefficient	SE	I^2 %	MA model
**25(OH)D**	SBP	-0.160	0.091	64.6	Random
	SBP (ii)	-0.149	0.092	67.0	Random
	DBP	-0.004	0.068	78.5	Random
	DBP (ii)	0.006	0.069	80.7	Random
CYP2R1	SBP	-0.187	0.130	11.7	Fixed
	SBP (ii)	-0.166	0.127	13.9	Fixed
	DBP	-0.107	0.081	20.8	Fixed
	DBP (ii)	-0.085	0.079	34.1	Fixed
DHCR7	SBP	-0.106	0.279	65.1	Random
	SBP (ii)	-0.114	0.272	65.1	Random
	DBP	-0.137	0.172	66.4	Random
	DBP (ii)	-0.148	0.160	64.2	Random
**Synthesis score**	SBP	-0.238	0.100	0	Fixed
	SBP (ii)	-0.230	0.099	3.4	Fixed
	DBP	-0.143	0.064	0	Fixed
	DBP (ii)	-0.132	0.062	6.0	Fixed

Supplementary table 9: Comparing genetically indexed association obtained by the Instrumental Variable (IV) ratio method [2-staged IV, as used in main analyses] and meta-analyses of study specific IV ratios [MA of IV ratios] in the Subsample of D-CarDia cohorts with individual level data and full information on *DHCR7*, *CYP2R1*, blood pressures and 25(OH)D (10 studies, n up to 34,609).

Outcome	Method	Coefficient*	SE	I^2 %	MA model
Systolic BP	2-staged IV	-0.085	0.039	28.9 [1st stage MA]<br>0 [2nd stage MA]	Fixed [both stages]
	MA of IV ratios	-0.076	0.035	0	Fixed
Diastolic BP	2-staged IV	-0.057	0.025	28.9 [1st stage MA]<br>0 [2nd stage MA]	Fixed [both stages]
	MA of IV ratios	-0.044	0.022	0	Fixed
Hypertension	2-staged IV	-0.005	0.005	28.9 [1st stage MA]<br>0 [2nd stage MA]	Fixed [both stages]
	MA of IV ratios	-0.005	0.005	0	Fixed

IV, Instrumental variable; MA, Meta-analysis

1st stage MA: meta-analysis of association of the instrument with the outcome

2nd stage MA: meta-analysis of association of the instrument with 25(OH)D

* The difference in BP mmHg or difference in log odds of hypertension, per % increase in 25(OH)D.

Supplementary table 10: Summary of coefficients for the instrumental variable (IV) ratio analyses in adult cohorts, using the Synthesis score and its components as instrumental variables. Includes summary data from ICBP/CHARGE/Global BPgen consortiums (N up to 146,581, overlapping studies excluded).

**Outcome**	**Synthesis score & its components**	**SNP with 25(OH)D, per allele**	**SNP with outcome, per allele**	**IV estimate for causal effect, per 10% increase in 25(OH)D**
		**Coefficient, % (95% CI)**	**Coefficient % (95% CI)**	**Coefficient (95% CI)**
Systolic blood pressure (mmHg)	*DHCR7*	3.29 (2.41, 4.17)	-0.11 (-0.39, 0.18)	-0.32 (-1.18, 0.54)
	CYP2R1	2.58 (2.12, 3.05)	-0.08 (-0.22, 0.05)	-0.32 (-0.86, 0.21)
	Synthesis score	2.83 (2.48, 3.18)	-0.10 (-0.21, -0.0001)	-0.37 (-0.73, 0.003)
Diastolic blood pressure (mmHg)	*DHCR7*	3.29 (2.41, 4.17)	-0.09 (-0.25, 0.08)	-0.26 (-0.78, 0.26)
	CYP2R1	2.58 (2.12, 3.05)	-0.09 (-0.18, -0.01)	-0.35 (-0.68, -0.03)
	Synthesis score	2.83 (2.48, 3.18)	-0.08 (-0.15, -0.02)	-0.29 (-0.52, -0.07)
Hypertension	*DHCR7*	3.29 (2.41, 4.17)	0.98 (0.95, 1.02)	0.95 (0.85, 1.07)
	CYP2R1	2.58 (2.12, 3.05)	0.98 (0.96, 1.00)	0.92 (0.85, 0.99)
	Synthesis score	2.83 (2.48, 3.18)	0.98 (0.96, 0.99)	0.92 (0.87, 0.97)

V. Supplementary figures

Supplementary figure 1: Flow chart showing the statistical analyses involved at each stage of the meta-analyses

Association of 25(OH)D with BP and hypertension

β_{XY}

Phenotype associations of 25(OH)D with SBP, DBP and hypertension were assessed in 17 D-CarDia studies with 25(OH)D measurements. Models were adjusted for age, age^2, BMI, sex, geographical site and/or principal components [as available/relevant]. SBP and DBP were corrected for use of BP medications (+15/10 mmHg respectively was added to the values of individuals taking BP medications).

Secondary analyses:

- Additional adjustment for serum triglycerides and total cholesterol (to exclude pleiotropic effects, since 25(OH)D is a cholesterol derivative)
- Tests for curvature in the relationships between 25(OH)D and SBP and DBP
- Models repeated stratified by sex
- Meta-regression by study level factors to assess possible sources of heterogeneity between studies
- Analyses repeated in two adolescent cohorts with 25(OH)D measurements (exploratory analysis due to small sample size)
- Different adjustments to SBP and DBP for use of BP medications (+10/5 instead of +15/10 mmHg respectively) were assessed in the subset of cohorts for which individual level data and measurements of 25(OH)D were available (10 D-CarDia studies)
- Mutually adjusted association of log transformed 25(OH)D with confounding factors in selected D-CarDia studies with individual level data

Association of vitamin D SNPs & scores with 25(OH)D

β_{ZX}

Four vitamin D SNPs and two unweighted allele scores ("synthesis" and "metabolism") were evaluated for association with 25(OH)D in 17 D-CarDia studies with 25(OH)D measurements.
Models were adjusted as above, plus adjustments for month of sample collection and laboratory batch [as available/relevant].

Secondary analyses:

- Meta-regression by study level factors to assess possible sources of heterogeneity between studies
- Effects in studies with genotyped SNPs compared to those with imputed SNPs
- Models repeated stratified by sex
- Analyses repeated in two adolescent cohorts with 25(OH)D measurements
- Association of the 25(OH)D increasing alleles in *DHCR7*, *CYP2R1*, synthesis score with confounders such as age, sex, season, BMI, total cholesterol and triglycerides)

Analyses were performed in each study according to a standardized analysis plan.

Study level results were combined using fixed effects meta-analysis, or random effects models in presence of heterogeneity.

Association of vitamin D SNPs & scores with BP and hypertension

β_{ZY}

Vitamin D SNPs/allele scores were evaluated for association with SBP, DBP and hypertension, with adjustments as above (see first box).
To increase statistical power, results from 31 D-CarDia studies were meta-analysed with data from the ICBP consortium when examining SBP or DBP as the outcome. Data from CHARGE and Global BPgen consortia were included in meta-analyses for hypertension. Some D-CarDia studies had taken part in these consortia, and their results were therefore not included in these meta-analyses. An approximation method was used to combine consortia look-up results from the SNPs into the allele scores.

Secondary analyses:

- Additional adjustment for serum triglycerides and total cholesterol, as available in 30 D-CarDia studies
- In the presence of heterogeneity, meta-regression by study level factors was done to assess possible sources of heterogeneity between studies
- Analyses repeated for metabolism score
- Analyses repeated in four adolescent cohorts
- Effects in studies with genotyped SNPs compared to those with imputed SNPs

Formal IV analysis

$$\beta_{IV} = \frac{\beta_{ZY}}{\beta_{ZX}}$$

Results from meta analyses in previous two boxes were combined using the Instrumental Variable (IV) ratio method to gain an estimate of the possible causal effect of 25(OH)D on SBP, DBP and hypertension.
e.g. the meta-analysed association of vitamin D synthesis allele score with SBP, DBP and hypertension was divided by the association of vitamin D synthesis allele score with 25(OH)D.

Secondary analyses:

- The calculation of IV ratio using meta-analysed coefficients was compared to a meta-analysis of study-level IV ratios in the subset of cohorts for which individual level data and measurements of 25(OH)D were available (10 D-CarDia studies)

Supplementary figure 2: Association of the 25-hydroxyvitamin D concentrations with systolic (A) and diastolic (B) blood pressures and hypertension (C) (D-CarDia, up to N=49,363). Coefficients corresponding to change in blood pressure (mmHg) or odds of hypertension per 10% increment in 25(OH)D

A **B** **C**

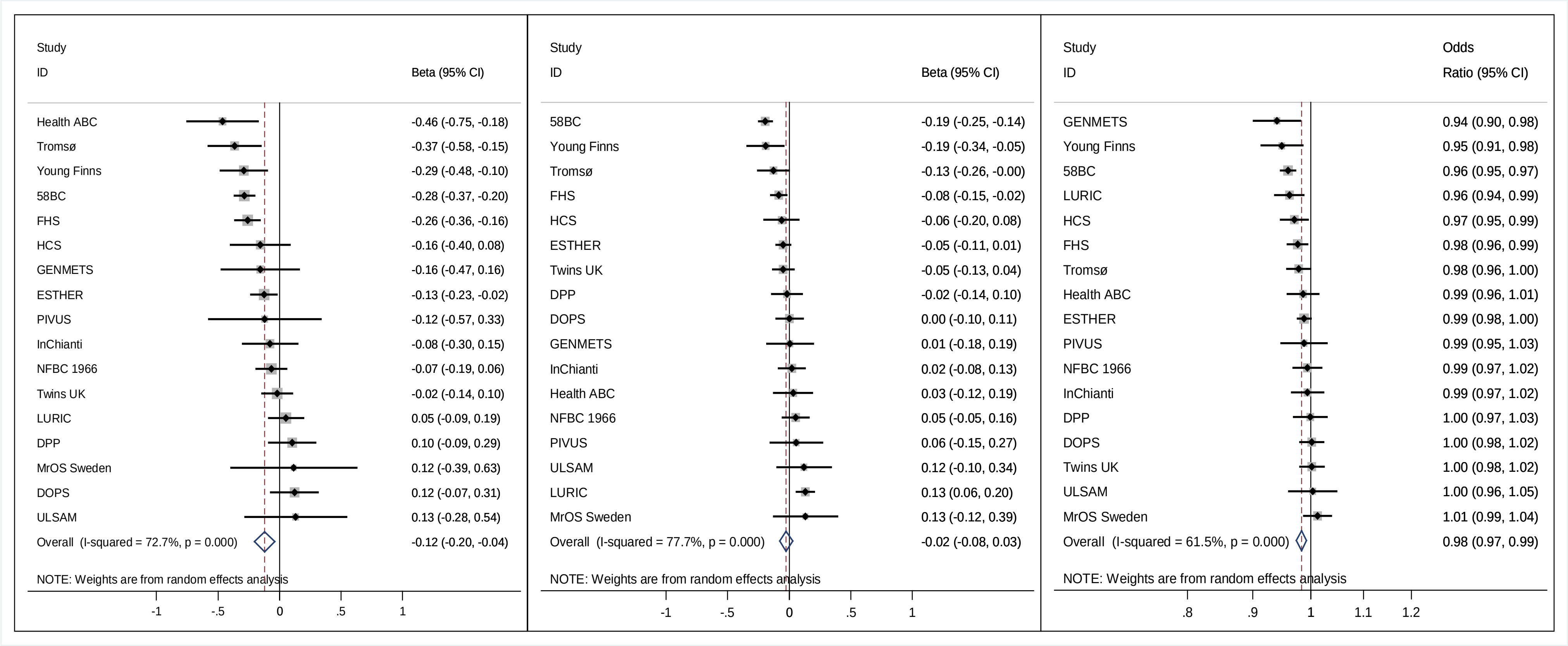

Supplementary figure 3A: Association of the four vitamin D SNPs with systolic blood pressure (meta-analysis of the results from D-CarDia and ICBP consortium, up to 146,581 adults (overlapping studies excluded) and 8,591 adolescents

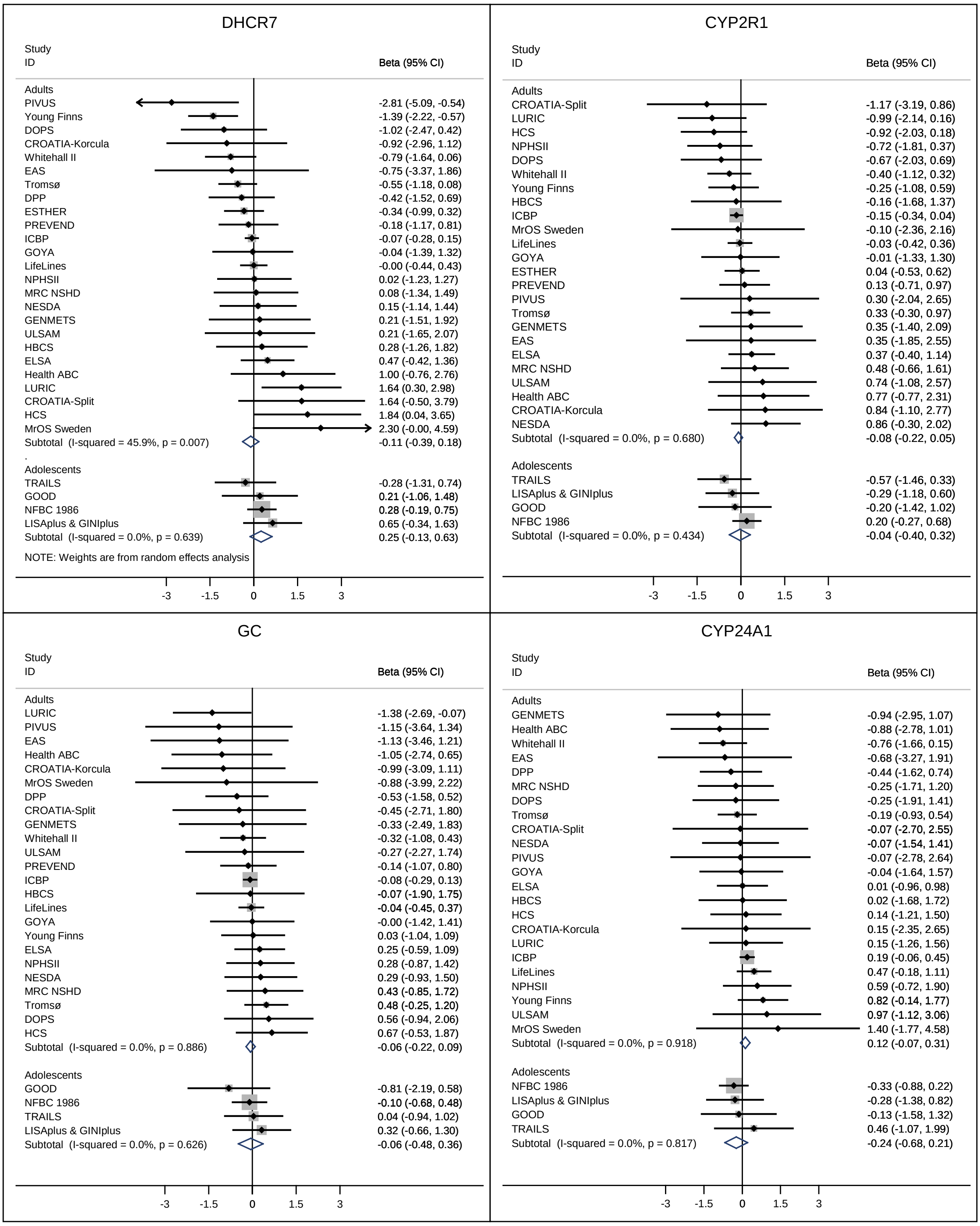

Supplementary figure 3B: Association of the four vitamin D SNPs with diastolic blood pressure (meta-analysis of the results from D-CarDia and ICBP consortium, up to 146,581 adults (overlapping studies removed) and 8,591 adolescents

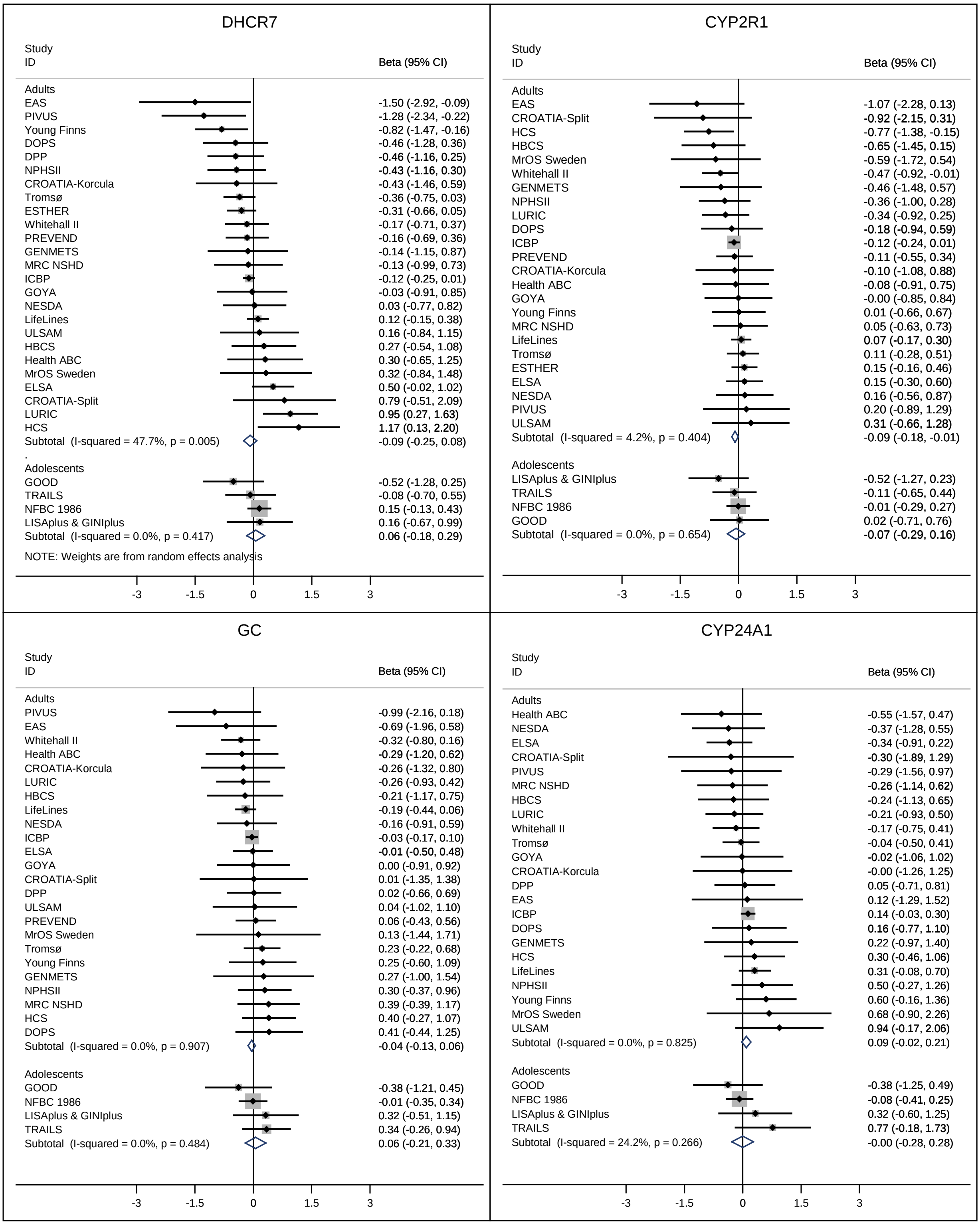

Supplementary figure 3C: Association of the four vitamin D SNPs with hypertension (meta-analysis of the results from D-CarDia, CHARGE and Global BPgen consortiums, up to N=142,255, overlapping studies excluded)

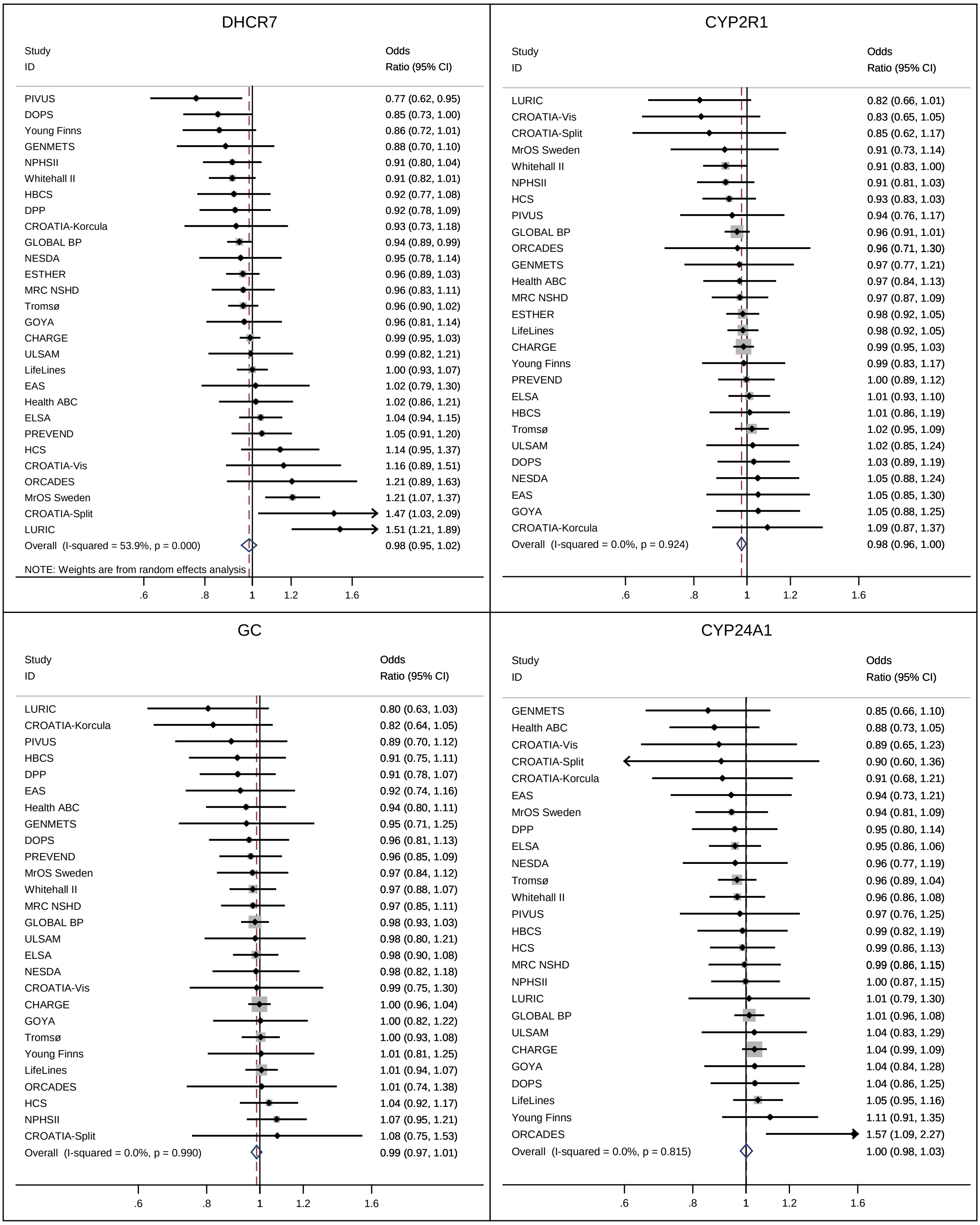

Supplementary figure 4: Association of the metabolism allele score with systolic (A) and diastolic (B) blood pressures and hypertension (C) (D-CarDia and ICBP/CHARGE/Global BPgen consortiums, up to 146,581 adults (overlapping studies removed) and 8,591 adolescents

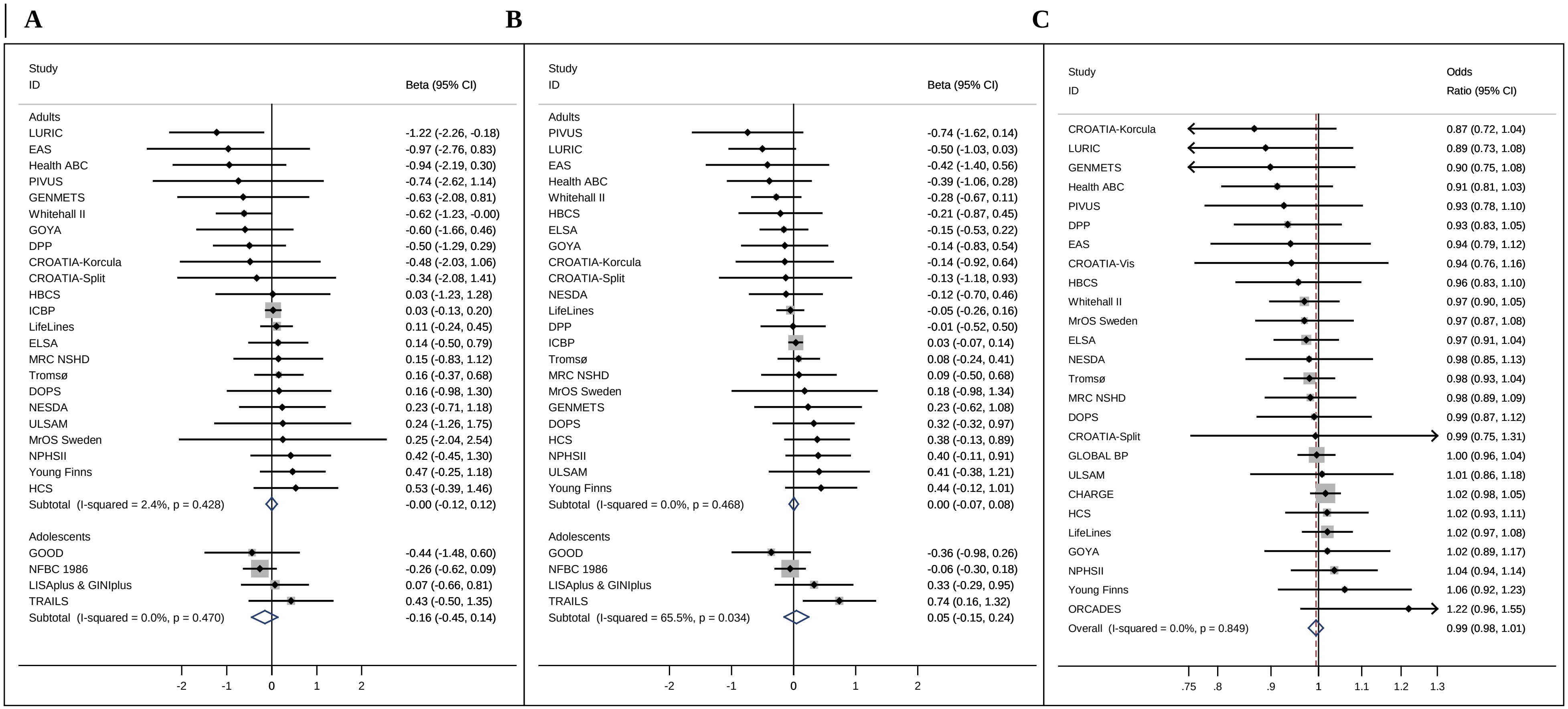

VI. Expanded author list

LifeLines Cohort Study

Behrooz Z Alizadeh, Rudolf A de Boer, H Marike Boezen, Marcel Bruinenberg, Lude Franke, Pim van der Harst, Hans L Hillege, Melanie M van der Klauw, Gerjan Navis, Johan Ormel, Dirkje S Postma, Judith GM Rosmalen, Joris P Slaets, Harold Snieder, Ronald P Stolk, Bruce HR Wolffenbuttel, Cisca Wijmenga.

ICBP consortium

Georg B. Ehret, Patricia B. Munroe, Kenneth M. Rice, Murielle Bochud, Andrew D. Johnson, Daniel I. Chasman, Albert V. Smith, Martin D. Tobin, Germaine C. Verwoert, Shih-Jen Hwang, Vasyl Pihur, Peter Vollenweider, Paul F. O'Reilly, Najaf Amin, Jennifer L Bragg-Gresham, Alexander Teumer, Nicole L. Glazer, Lenore Launer, Jing Hua Zhao, Yurii Aulchenko, Simon Heath, Siim Sõber, Afshin Parsa, Jian'an Luan, Pankaj Arora, Abbas Dehghan, Feng Zhang, Gavin Lucas, Andrew A. Hicks, Anne U. Jackson, John F Peden, Toshiko Tanaka, Sarah H. Wild, Igor Rudan, Wilmar Igl, Yuri Milaneschi, Alex N. Parker, Cristiano Fava, John C. Chambers, Ervin R. Fox, Meena Kumari, Min Jin Go, Pim van der Harst, Wen Hong Linda Kao, Marketa Sjögren, D. G. Vinay, Myriam Alexander, Yasuharu Tabara, Sue Shaw-Hawkins, Peter H. Whincup, Yongmei Liu, Gang Shi, Johanna Kuusisto, Bamidele Tayo, Mark Seielstad, Xueling Sim, Khanh-Dung Hoang Nguyen, Terho Lehtimäki, Giuseppe Matullo, Ying Wu, Tom R. Gaunt, N. Charlotte Onland-Moret, Matthew N. Cooper, Carl G.P. Platou, Elin Org, Rebecca Hardy, Santosh Dahgam, Jutta Palmen, Veronique Vitart, Peter S. Braund, Tatiana Kuznetsova, Cuno S.P.M. Uiterwaal, Adebowale Adeyemo, Walter Palmas, Harry Campbell, Barbara Ludwig, Maciej Tomaszewski, Ioanna Tzoulaki, Nicholette D. Palmer, CARDIoGRAM consortium, CKDGen Consortium, KidneyGen Consortium, EchoGen consortium, CHARGE-HF consortium, Thor Aspelund, Melissa Garcia, Yen-Pei C. Chang, Jeffrey R. O'Connell, Nanette I. Steinle, Diederick E. Grobbee, Dan E. Arking, Sharon L. Kardia, Alanna C. Morrison, Dena Hernandez, Samer Najjar, Wendy L. McArdle, David Hadley, Morris J. Brown, John M. Connell, Aroon D. Hingorani, Ian N.M. Day, Debbie A. Lawlor, John P. Beilby, Robert W. Lawrence, Robert Clarke, Rory Collins, Jemma C Hopewell, Halit Ongen, Albert W. Dreisbach, Yali Li, J H. Young, Joshua C. Bis, Mika Kähönen, Jorma Viikari, Linda S. Adair, Nanette R. Lee, Ming-Huei Chen, Matthias Olden, Cristian Pattaro, Judith A. Hoffman Bolton, Anna Köttgen, Sven Bergmann, Vincent Mooser, Nish Chaturvedi, Timothy M. Frayling,

Muhammad Islam, Tazeen H. Jafar, Jeanette Erdmann, Smita R. Kulkarni, Stefan R. Bornstein, Jürgen Grässler, Leif Groop, Benjamin F. Voight, Johannes Kettunen, Philip Howard, Andrew Taylor, Simonetta Guarrera, Fulvio Ricceri, Valur Emilsson, Andrew Plump, Inês Barroso, Kay-Tee Khaw, Alan B. Weder, Steven C. Hunt, Yan V. Sun, Richard N. Bergman, Francis S. Collins, Lori L. Bonnycastle, Laura J. Scott, Heather M. Stringham, Leena Peltonen, Markus Perola, Erkki Vartiainen, Stefan-Martin Brand, Jan A. Staessen, Thomas J. Wang, Paul R. Burton, Maria Soler Artigas, Yanbin Dong, Harold Snieder, Xiaoling Wang, Haidong Zhu, Kurt K. Lohman, Megan E. Rudock, Susan R Heckbert, Nicholas L Smith, Kerri L Wiggins, Ayo Doumatey, Daniel Shriner, Gudrun Veldre, Margus Viigimaa, Sanjay Kinra, Dorairajan Prabhakaran, Vikal Tripathy, Carl D. Langefeld, Annika Rosengren, Dag S. Thelle, Anna Maria Corsi, Andrew Singleton, Terrence Forrester, Gina Hilton, Colin A. McKenzie, Tunde Salako, Naoharu Iwai, Yoshikuni Kita, Toshio Ogihara, Takayoshi Ohkubo, Tomonori Okamura, Hirotsugu Ueshima, Satoshi Umemura, Susana Eyheramendy, Thomas Meitinger, H.-Erich Wichmann, Yoon Shin Cho, Hyung-Lae Kim, Jong-Young Lee, James Scott, Joban S. Sehmi, Weihua Zhang, Bo Hedblad, Peter Nilsson, George Davey Smith, Andrew Wong, Narisu Narisu, Alena Stančáková, Leslie J. Raffel, Jie Yao, Sekar Kathiresan, Chris O'Donnell, Stephen M. Schwartz, M. Arfan Ikram, W. T. Longstreth Jr., Thomas H. Mosley, Sudha Seshadri, Nick R.G. Shrine, Louise V. Wain, Mario A. Morken, Amy J. Swift, Jaana Laitinen, Inga Prokopenko, Paavo Zitting, Jackie A. Cooper, Steve E. Humphries, John Danesh, Asif Rasheed, Anuj Goel, Anders Hamsten, Hugh Watkins, Stephan J.L. Bakker, Wiek H. van Gilst, Charles S. Janipalli, K. Radha Mani, Chittaranjan S. Yajnik, Albert Hofman, Francesco U.S. Mattace-Raso, Ben A. Oostra, Ayse Demirkan, Aaron Isaacs, Fernando Rivadeneira, Edward G Lakatta, Marco Orru, Angelo Scuteri, Mika Ala-Korpela, Antti J Kangas, Leo-Pekka Lyytikäinen, Pasi Soininen, Taru Tukiainen, Peter Würtz, Rick Twee-Hee Ong, Marcus Dörr, Heyo K. Kroemer, Uwe Völker, Henry Völzke, Pilar Galan, Serge Hercberg, Mark Lathrop, Diana Zelenika, Panos Deloukas, Massimo Mangino, Tim D. Spector, Guangju Zhai, James F. Meschia, Michael A. Nalls, Pankaj Sharma, Janos Terzic, M. J. Kranthi Kumar, Matthew Denniff, Ewa Zukowska-Szczechowska, Lynne E. Wagenknecht, F. Gerald R. Fowkes, Fadi J. Charchar, Peter E.H. Schwarz, Caroline Hayward, Xiuqing Guo, Charles Rotimi, Michiel L. Bots, Eva Brand, Nilesh J. Samani, Ozren Polasek, Philippa J. Talmud, Fredrik Nyberg, Diana Kuh, Maris Laan, Kristian Hveem, Lyle J. Palmer, Yvonne T. van der Schouw, Juan P. Casas, Karen L. Mohlke, Paolo Vineis, Olli Raitakari, Santhi K. Ganesh, Tien Y. Wong, E Shyong Tai, Richard S. Cooper, Markku Laakso, Dabeeru C. Rao, Tamara B. Harris, Richard W. Morris, Anna F. Dominiczak, Mika Kivimaki, Michael G. Marmot, Tetsuro Miki, Danish Saleheen, Giriraj R. Chandak, Josef Coresh, Gerjan Navis, Veikko Salomaa, Bok-Ghee Han,

Xiaofeng Zhu, Jaspal S. Kooner, Olle Melander, Paul M Ridker, Stefania Bandinelli, Ulf B. Gyllensten, Alan F. Wright, James F. Wilson, Luigi Ferrucci, Martin Farrall, Jaakko Tuomilehto, Peter P. Pramstaller, Roberto Elosua, Nicole Soranzo, Eric J.G. Sijbrands, David Altshuler, Ruth J.F. Loos, Alan R. Shuldiner, Christian Gieger, Pierre Meneton, Andre G. Uitterlinden, Nicholas J. Wareham, Vilmundur Gudnason, Jerome I. Rotter, Rainer Rettig, Manuela Uda, David P. Strachan, Jacqueline C.M. Witteman, Anna-Liisa Hartikainen, Jacques S. Beckmann, Eric Boerwinkle, Ramachandran S. Vasan, Michael Boehnke, Martin G. Larson, Marjo-Riitta Järvelin, Bruce M. Psaty, Gonçalo R Abecasis, Aravinda Chakravarti, Paul Elliott, Cornelia M. van Duijn, Christopher Newton-Cheh, Daniel Levy, Mark J. Caulfield, Toby Johnson.

CHARGE consortium

Daniel Levy, Georg B Ehret, Kenneth Rice, Germaine C Verwoert, Lenore J Launer, Abbas Dehghan, Nicole L Glazer, Alanna C Morrison, Andrew D Johnson, Thor Aspelund, Yurii Aulchenko, Thomas Lumley, Anna Köttgen, Ramachandran S Vasan, Fernando Rivadeneira, Gudny Eiriksdottir, Xiuqing Guo, Dan E Arking, Gary F Mitchell, Francesco U S Mattace-Raso, Albert V Smith, Kent Taylor, Robert B Scharpf, Shih-Jen Hwang, Eric J G Sijbrands, Joshua Bis, Tamara B Harris, Santhi K Ganesh, Christopher J O'Donnell, Albert Hofman, Jerome I Rotter, Josef Coresh, Emelia J Benjamin, André G Uitterlinden, Gerardo Heiss, Caroline S Fox, Jacqueline C M Witteman, Eric Boerwinkle, Thomas J Wang, Vilmundur Gudnason, Martin G Larson, Aravinda Chakravarti, Bruce M Psaty, Cornelia M van Duijn.

Global BPgen consortium

Christopher Newton-Cheh, Toby Johnson, Vesela Gateva, Martin D Tobin, Murielle Bochud, Lachlan Coin, Samer S Najjar, Jing Hua Zhao, Simon C Heath, Susana Eyheramendy, Konstantinos Papadakis, Benjamin F Voight, Laura J Scott, Feng Zhang, Martin Farrall, Toshiko Tanaka, Chris Wallace, John C Chambers, Kay-Tee Khaw, Peter Nilsson, Pim van der Harst, Silvia Polidoro, Diederick E Grobbee, N Charlotte Onland-Moret, Michiel L Bots, Louise V Wain, Katherine S Elliott, Alexander Teumer, Jian'an Luan, Gavin Lucas, Johanna Kuusisto, Paul R Burton, David Hadley, Wendy L McArdle, Wellcome Trust Case Control Consortium, Morris Brown, Anna Dominiczak, Stephen J Newhouse, Nilesh J Samani, John Webster, Eleftheria Zeggini, Jacques S Beckmann, Sven Bergmann, Noha Lim, Kijoung Song, Peter Vollenweider, Gerard Waeber, Dawn M Waterworth, Xin Yuan, Leif Groop, Marju Orho-Melander, Alessandra Allione, Alessandra Di Gregorio, Simonetta Guarrera, Salvatore Panico,

Fulvio Ricceri, Valeria Romanazzi, Carlotta Sacerdote, Paolo Vineis, Inês Barroso, Manjinder S Sandhu, Robert N Luben, Gabriel J Crawford, Pekka Jousilahti, Markus Perola, Michael Boehnke, Lori L Bonnycastle, Francis S Collins, Anne U Jackson, Karen L Mohlke, Heather M Stringham, Timo T Valle, Cristen J Willer, Richard N Bergman, Mario A Morken, Angela Döring, Christian Gieger, Thomas Illig, Thomas Meitinger, Elin Org, Arne Pfeufer, H Erich Wichmann, Sekar Kathiresan, Jaume Marrugat, Christopher J O'Donnell, Stephen M Schwartz, David S Siscovick, Isaac Subirana, Nelson B Freimer, Anna-Liisa Hartikainen, Mark I McCarthy, Paul F O'Reilly, Leena Peltonen, Anneli Pouta, Paul E de Jong, Harold Snieder, Wiek H van Gilst, Robert Clarke, Anuj Goel, Anders Hamsten, John F Peden, Udo Seedorf, Ann-Christine Syvänen, Giovanni Tognoni, Edward G Lakatta, Serena Sanna, Paul Scheet, David Schlessinger, Angelo Scuteri, Marcus Dörr, Florian Ernst, Stephan B Felix, Georg Homuth, Roberto Lorbeer, Thorsten Reffelmann, Rainer Rettig, Uwe Völker, Pilar Galan, Ivo G Gut, Serge Hercberg, G Mark Lathrop, Diana Zelenika, Panos Deloukas, Nicole Soranzo, Frances M Williams, Guangju Zhai, Veikko Salomaa, Markku Laakso, Roberto Elosua, Nita G Forouhi, Henry Völzke, Cuno S Uiterwaal, Yvonne T van der Schouw, Mattijs E Numans, Giuseppe Matullo, Gerjan Navis, Göran Berglund, Sheila A Bingham, Jaspal S Kooner, John M Connell, Stefania Bandinelli, Luigi Ferrucci, Hugh Watkins, Tim D Spector, Jaakko Tuomilehto, David Altshuler, David P Strachan, Maris Laan, Pierre Meneton, Nicholas J Wareham, Manuela Uda, Marjo-Riitta Järvelin, Vincent Mooser, Olle Melander, Ruth JF Loos, Paul Elliott, Gonçalo R Abecasis, Mark Caulfield & Patricia B Munroe.

VII. Study acknowledgments

1. 1958 Birth cohort (1958BC): The 25-hydroxyvitamin D assays were funded by the BUPA foundation. DNA collection was funded by MRC grant G0000934 and cell-line creation by Wellcome Trust grant 068545/Z/02. This research used resources provided by the Type 1 Diabetes Genetics Consortium, a collaborative clinical study sponsored by the National Institute of Diabetes and Digestive and Kidney Diseases (NIDDK), National Institute of Allergy and Infectious Diseases, National Human Genome Research Institute, National Institute of Child Health and Human Development, and Juvenile Diabetes Research Foundation International (JDRF) and supported by U01 DK062418. This study makes use of data generated by the Wellcome Trust Case-Control Consortium. A full list of investigators who contributed to generation of the data is available from the Wellcome Trust Case-Control Consortium website. Funding for the project was provided by the Wellcome Trust under the award 076113. Great Ormond Street Hospital/University College London, Institute of Child Health and Oxford Biomedical Research Centre, University of Oxford receive a proportion of funding from the Department of Health's National Institute for Health Research (NIHR) ('Biomedical Research Centres' funding). This paper presents independent research and the views expressed are those of the author(s) and not necessarily those of the NHS, the NIHR, or the Department of Health. Mark I. McCarthy acknowledges support from the EU Framework 7 ENGAGE (HEALTH-F4-2007-201413) and The Wellcome Trust (098381 and 090367).

2. CROATIA-Korcula: The CROATIA-Korcula study was funded by grants from the Medical Research Council (UK), European Commission Framework 6 project EUROSPAN (Contract No. LSHG-CT-2006-018947) and Republic of Croatia Ministry of Science, Education and Sports research grants to I.R. (108-1080315-0302). We would like to acknowledge the invaluable contributions of the recruitment team in Korcula, the administrative teams in Croatia and Edinburgh and the people of Korcula. The SNP genotyping for the CROATIA-Korcula cohort was performed in Helmholtz Zentrum München, Neuherberg, Germany.

3. CROATIA-Split: The CROATIA-Split study is funded by grants from the Medical Research Council (UK), European Commission Framework 6 project EUROSPAN (Contract No. LSHG-CT-2006-018947) and Republic of Croatia Ministry of Science, Education and Sports research grants to I.R.

(108-1080315-0302). We would like to acknowledge the staff of several institutions in Croatia that supported the field work, including but not limited to The University of Split and Zagreb Medical Schools and the Croatian Institute for Public Health. The SNP genotyping for the CROATIA-Split cohort was performed by AROS Applied Biotechnology, Aarhus, Denmark.

4. CROATIA-Vis: The CROATIA-Vis study was funded by grants from the Medical Research Council (UK) and Republic of Croatia Ministry of Science, Education and Sports research grants to I.R. (108-1080315-0302). We would like to acknowledge the staff of several institutions in Croatia that supported the field work, including but not limited to The University of Split and Zagreb Medical Schools, the Institute for Anthropological Research in Zagreb and Croatian Institute for Public Health. The SNP genotyping for the CROATIA-Vis cohort was performed in the core genotyping laboratory of the Wellcome Trust Clinical Research Facility at the Western General Hospital, Edinburgh, Scotland.

5. Danish Osteoporosis Prevention Study (DOPS): Genotyping was funded by the British Heart Foundation (grant PG/09/023). The study was supported by grants from the Karen Elise Jensen’s Foundation, Aarhus University, and the Danish Medical Research Council. A list of centres and investigators are available at www.ClinicalTrials.gov (NCT00252408).

6. Diabetes Prevention Program (DPP): The NIDDK of the National Institutes of Health provided funding to the clinical centers and the Coordinating Center for the design and conduct of the study; collection, management, analysis, and interpretation of the data. The Southwestern American Indian Centers were supported directly by the NIDDK and the Indian Health Service. The General Clinical Research Center Program, National Center for Research Resources supported data collection at many of the clinical centers. Funding for data collection and participant support was also provided by the Office of Research on Minority Health, the National Institute of Child Health and Human Development, the National Institute on Aging, the Centers for Disease Control and Prevention, Office of Research on Women's Health, the Department of Veterans Affairs, and the American Diabetes Association. The opinions expressed are those of the investigators and do not necessarily reflect the views of the Indian Health

Service or other funding agencies. A complete list of centers, investigators, and staff can be found in the previously published study (The Diabetes Prevention Program: baseline characteristics of the randomized cohort. The Diabetes Prevention Program Research Group. Diabetes Care. 2000; 23(11): 1619-29).

The investigators gratefully acknowledge the commitment and dedication of all participants in the DPP, without whom this work would not have been possible. This work was funded by R01 DK072041 to JCF and KAJ. JCF has received consulting honoraria from Lilly.

7. Edinburgh Artery Study (EAS): We thank all participants and staff involved in the EAS. The study was funded by the British Heart Foundation.

8. English Longitudinal Study of Ageing (ELSA): Genotyping was funded by the British Heart Foundation (grant PG/09/023). Samples from the English Longitudinal Study of Ageing (ELSA) DNA Repository (EDNAR), received support under a grant (AG1764406S1) awarded by the National Institute on Ageing (NIA). ELSA was developed by a team of researchers based at the National Centre for Social Research, University College London and the Institute of Fiscal Studies. The data were collected by the National Centre for Social Research.

9. ESTHER: Vitamin D measurements were performed in the context of the German Cancer Aid project number 108250 and 108426 and the CHANCES project funded in the FP7 framework programme of DG-RESEARCH in the European Commission (Grant no. 242244). The ESTHER study was funded by the Baden-Württemberg state Ministry of Science, Research and Arts (Stuttgart, Germany), the Federal Ministry of Education and Research (Berlin, Germany) and the Federal Ministry of Family Affairs, Senior Citizens, Women and Youth (Berlin, Germany).

10. Framingham Heart Study (FHS): The Framingham Heart Study of the National Heart, Lung and Blood Institute of the National Institutes of Health and Boston University School of Medicine is supported by the NIH/NHLBI contract N01-HC-25195. The analyses reflect intellectual input and resource development from the Framingham Heart Study investigators participating in the SNP Health Association Resource (SHARe) project. This work was partially supported by a contract with Affymetrix, Inc for genotyping services (Contract No. N02-HL-6-4278). A portion of this research utilized the Linux Cluster for Genetic Analysis (LinGA-II) funded by the

Robert Dawson Evans Endowment of the Department of Medicine at Boston University School of Medicine and Boston Medical Center. Funding was also received from the American Heart Association.

11. Gothenburg Osteoporosis and Obesity Determinants (GOOD) Study: This study was supported by the Swedish Research Council, the Lundberg Foundation, and ALF/LUA grants from the Sahlgrenska, University Hospital.

12. Health2000 GenMets (GENMETS) Study: V.S. was supported by grants #139635 and #129494 from the Academy of Finland and the Finnish Foundation for Cardiovascular Research.

13. Health, Aging and Body Composition (Health ABC) Study: We also thank Yongmei Liu (Co-PI of Health ABC GWA study grant), who obtained GWA data, reviewed the analysis proposal and the manuscript draft.

The Health ABC study was supported by the Intramural Research Program of the National Institutes of Health, National Institute on Aging and National Institute on Aging contracts N01-AG-6-2101, N01-AG-6-2103, and N01-AG-6-2106, NIA grant R01-AG028050, and NINR grant R01-NR012459. Assessment of 25-hydroxyvitamin D concentrations was funded by a National Institute on Aging grant, R01-AG029364. The genome-wide association study was funded by a National Institute on Aging grant, R01-AG032098, and genotyping services were provided by the Center for Inherited Disease Research (CIDR). CIDR is fully funded through a federal contract from the National Institutes of Health to The Johns Hopkins University (contract number HHSN268200782096C).

14. Helsinki Birth Cohort Study (HBCS): Genotyping was performed at Sanger Institute which is acknowledged. Helsinki Birth Cohort Study has been supported by grants from the Academy of Finland (Grant No. 120386 and 125876 to JGE), the Finnish Diabetes Research Society, Folkhälsan Research Foundation, Finska Läkaresällskapet,Liv och Hälsa, Samfundet Folkhälsan, Finska Läkaresällskapet and the Signe and Ane Gyllenberg foundation.

15. Hertfordshire cohort study (HCS): Genotyping was in part funded by the British Heart Foundation (grant PG/09/023). The HCS is supported by the Medical Research Council of Great Britain; Arthritis Research, UK; the British Heart Foundation; Porticus Foundation; the International Osteoporosis Foundation and the NIHR Biomedical Research Unit in Nutrition, University of Southampton, UK; and the NIHR Musculoskeletal Biomedical Research Unit, University of Oxford, UK.

16. InCHIANTI study: The research leading to the results from InCHIANTI study received funding from European Community's Seventh Framework Programme (FP7/2007-2013) as a Marie Curie Initial Training Networks project (FP7-PEOPLE-ITN-2008) under grant agreement number 238821 (Biology of Liver and Pancreatic Development and Disease).

17. LifeLines Cohort Study: The LifeLines Cohort Study, and generation and management of GWA genotype data for the LifeLines Cohort Study is supported by the Netherlands Organization of Scientific Research NWO (grant 175.010.2007.006), the Economic Structure Enhancing Fund (FES) of the Dutch government, the Ministry of Economic Affairs, the Ministry of Education, Culture and Science, the Ministry for Health, Welfare and Sports, the Northern Netherlands Collaboration of Provinces (SNN), the Province of Groningen, University Medical Center Groningen, the University of Groningen, Dutch Kidney Foundation and Dutch Diabetes Research Foundation. RADB has consulted for and received research grants from Abbott. We acknowledge the contribution of all the members of the Lifelines Cohort study (members listed in the appendix).

LifeLines Scientific Protocol Preparation: Rudolf de Boer, Hans Hillege, Melanie van der Klauw, Gerjan Navis, Hans Ormel, Dirkje Postma, Judith Rosmalen, Joris Slaets, Ronald P. Stolk, Bruce Wolffenbuttel; LifeLines GWA Working Group: Behrooz Alizadeh, Marike Boezen, Marcel Bruinenberg, Noortje Festen, Lude Franke, Pim van der Harst, Gerjan Navis, Dirkje Postma, Harold Snieder, Cisca Wijmenga, Bruce Wolffenbuttel.

18. Lifestyle- Imune System- Allergy plus environment and genetics study (LISAplus) and German Infant Study on the influence of Nutrition Intervention plus environment and genetics (GINIplus): This study was supported by Ludwig-Maximilians-University's innovative research priority project MC-Health (subproject I). We thank all families for participation in the studies; the obstetric units for allowing recruitment, the GINI and LISA study teams for excellent work.

19. Ludwigshafen Risk and Cardiovascular Health (LURIC) Study: We extend our appreciation to the participants of the LURIC study; without their collaboration, participating in the collaboration would not have been possible. We thank the LURIC study team who were either temporarily or permanently involved in patient recruitment as well as sample and data handling, in addition to the laboratory staff at the Ludwigshafen General Hospital and the Universities of Freiburg and Ulm, Germany.

LURIC has received funding from the 6th Framework Program (integrated project Bloodomics, grant LSHM-CT-2004-503485) and from the 7th Framework Program (Atheroremo, grant agreement number 201668 and RiskyCAD, grant agreement number 305739) of the European Union as well as from the INTERREG IV Oberrhein Program (Project A28, Genetic mechanisms of cardiovascular diseases) with support from the European Regional Development Fund (ERDF) and the Wissenschaftsoffensive TMO.

20. Males from Genetics of extremely Overweight Young Adults (GOYA): We acknowledge Prof. George Davey Smith from the MRC CAiTE, University of Bristol, Bristol, UK who has contributed in conceiving and designing the experiments for this study. We also thank Wellcome Trust (http://www.wellcome.ac.uk/) for providing the finances to carry out the study.

This study was conducted as part of the activities of the Danish Obesity Research Centre (DanORC, www.danorc.dk) and the MRC centre for Causal Analyses in Translational Epidemiology (MRC CAiTE). We are thankful to all the volunteers, technicians, research scientists who contributed to the GOYA study.

21 MRC National Survey of Health & Development (MRC NSHD Birth Cohort): This work was funded by the Medical Research Council (MC_UU_12019/1). Genotyping was funded by the British Heart Foundation (grant PG/09/023). We are very grateful to the members of this birth cohort for their continuing interest and participation in the study. We would like to acknowledge the Swallow group, UCL, who performed the DNA extractions (Rousseau, et al 2006).

22. Netherlands Study of Depression and Anxiety (NESDA): The infrastructure for the NESDA study is funded through the Geestkracht programme of the Dutch Scientific Organization (ZON-MW, grant number 10-000-1002) and matching funds from participating universities and mental health care organizations. Genotyping in NESDA was funded by the Genetic Association Information Network (GAIN) of the Foundation for the US National Institutes of Health. Statistical analyses were carried out

on the Genetic Cluster Computer (http://www.geneticcluster.org), which is financially supported by the Netherlands Scientific Organization (NWO 480-05-003) along with a supplement from the Dutch Brain Foundation.

23. Northern Finland Birth Cohort 1986 (NFBC 1986) and Northern Finland Birth Cohort 1966 (NFBC1966): For NFBC1966, the 25-hydroxyvitamin D assays were funded by the (MRC; grant G0601653). Genotyping in NFBC1986 was funded by the British Heart Foundation (grant PG/09/023). NFBC programme received financial support from the Academy of Finland (project grants 104781, 120315, 129269, 129418, 1114194, 24300796, Center of Excellence in Complex Disease Genetics and SALVE), University Hospital Oulu, Biocenter, University of Oulu, Finland (75617), NHLBI grant 5R01HL087679-02 through the STAMPEED program (1RL1MH083268-01), NIH/NIMH (5R01MH63706:02), ENGAGE project and grant agreement HEALTH-F4-2007-201413, EU FP7 small-scale focused research collaborative project EurHEALTHAgeing 277849, and the Medical Research Council, UK (G0500539, G0600705, G1002319, PrevMetSyn/SALVE). The DNA extractions, sample quality controls, biobank up-keeping and aliquotting was performed in the National Public Health Institute, Biomedicum Helsinki, Finland and supported financially by the Academy of Finland and Biocentrum Helsinki. We thank the late Professor Paula Rantakallio (launch of NFBC1966), and Ms Outi Tornwall and Ms Minttu Jussila (DNA biobanking). The authors would like to acknowledge the contribution of the late Academian of Science Leena Peltonen.**:** The research of V.L. is funded in part through the European Community's Seventh Framework Programme (FP7/2007-2013), ENGAGE project, grant agreement HEALTH-F4-2007- 201413. The research of I.P. is funded in part through the European Community's Seventh Framework Programme (FP7/2007-2013), ENGAGE project, grant agreement HEALTH-F4-2007-201413.

24. Orkney Complex Disease Study (ORCADES): ORCADES was supported by the Chief Scientist Office of the Scottish Government, the Royal Society, the MRC Human Genetics Unit, Arthritis Research UK and the European Union framework program 6 EUROSPAN project (contract no. LSHG-CT-2006-018947). DNA extractions were performed at the Wellcome Trust Clinical Research Facility in Edinburgh. We would like to acknowledge the research nurses in Orkney, the administrative team in Edinburgh and the people of Orkney.

25. Osteoporotic Fractures in Men Study – Sweden (MrOS Sweden): This work was supported by the Swedish Research Council, the Swedish Foundation for Strategic Research, the ALF/LUA Research Grant in Gothenburg, the Lundberg Foundation, the Torsten and Ragnar Söderberg's Foundation, the Novo Nordisk Foundation, Magnus Bergvall Foundation, Åke Wiberg Foundation, Tore Nilson Foundation, and the Swedish Society for Medical Research.

26. Prevention of Renal and Vascular Endstage Disease (PREVEND): PREVEND genetics is supported by the Dutch Kidney Foundation (Grant E033), the EU project grant GENECURE (FP-6 LSHM CT 2006 037697), the National Institutes of Health (grant LM010098), The Netherlands organisation for health research and development (NWO-Groot grant 175.010.2007.006, NWO VENI grant 916.761.70, ZonMw grant 90.700.441), and the Dutch Inter University Cardiology Institute Netherlands (ICIN).

27. Prospective Investigation of the Vasculature in Uppsala Seniors (PIVUS) and Uppsala Longitudinal Study of Adult Men (ULSAM): PIVUS and ULSAM studies were supported by the Swedish Research Council and Knut and Alice Wallenberg Foundation. Genotyping was performed by the SNP Technology Platform at Uppsala University, Sweden (http://www.genotyping.se).

28. Second Northwick Park Heart Study (NPHSII): Genotyping was funded by the British Heart Foundation (grant PG/09/023). NPHSII was supported by the British Medical Research Council, the US National Institute of Health (grant NHLBI 33014) and Du Pont Pharma, Wilmington, USA. JC JA and SEH are supported by the British Heart Foundation (PG08/008).

29. TRacking Adolescents' Individual Lives Survey (TRAILS): This research is part of the TRacking Adolescents' Individual Lives Survey (TRAILS). Participating centers of TRAILS include various departments of the University Medical Center and University of Groningen, the Erasmus University Medical Center Rotterdam, the University of Utrecht, the Radboud Medical Center Nijmegen, and the Parnassia Bavo group, all in the Netherlands. TRAILS has been financially supported by various grants from the Netherlands Organization for Scientific Research NWO (Medical Research Council program grant GB-MW 940-38-011; ZonMW Brainpower grant 100-001-004; ZonMw Risk Behavior and Dependence grants 60-60600-97-118; ZonMw Culture and Health grant 261-98-710; Social Sciences Council medium-sized investment grants GB-MaGW 480-01-006 and GB-MaGW 480-07-001; Social Sciences Council project grants GB-MaGW 452-04-314 and GB-MaGW 452-06-004; NWO large-sized

investment grant 175.010.2003.005; NWO Longitudinal Survey and Panel Funding 481-08-013), the Dutch Ministry of Justice (WODC), the European Science Foundation (EuroSTRESS project FP-006), Biobanking and Biomolecular Resources Research Infrastructure BBMRI-NL (CP 32), and the participating universities. We are grateful to all adolescents, their parents and teachers who participated in this research and to everyone who worked on this project and made it possible. Statistical analyses were carried out on the Genetic Cluster Computer (http://www.geneticcluster.org), which is financially supported by the Netherlands Scientific Organization (NWO 480-05-003) along with a supplement from the Dutch Brain Foundation.

30. Tromsø study: The study was supported by grants from The North Norway Regional Health Authority, the Norwegian Diabetes Association, and the University of Tromsø.

31. Twins UK: The study was funded by the Wellcome Trust; European Community's Seventh Framework Programme (FP7/2007-2013). The study also receives support from the National Institute for Health Research (NIHR)- funded BioResource, Clinical Research Facility and Biomedical Research Centre based at Guy's & St Thomas' NHS Foundation Trust in partnership with King's College London. Tim Spector is an NIHR senior Investigator and is holder of an ERC Advanced Principal Investigator award. SNP Genotyping was performed by The Wellcome Trust Sanger Institute and National Eye Institute via NIH/CIDR.

32. Whitehall II (WHII) Study: Genotyping was funded by the British Heart Foundation (grant PG/09/023). The Whitehall-II study was supported by the Medical Research Council (K013351), the BHF, and the National Institutes of Health (R01HL36310).

33. Young Finns Study: S.R. was supported by the Academy of Finland Center of Excellence in Complex Disease Genetics (#213506 and #129680), Academy of Finland (#251217), the Finnish foundation for Cardiovascular Research and the Sigrid Juselius Foundation. The Young Finns Study has been financially supported by the Academy of Finland: grants 134309 (Eye), 126925, 121584, 124282, 129378 (Salve), 117787 (Gendi), and 41071 (Skidi), the Social Insurance Institution of Finland, Kuopio, Tampere and Turku University Hospital Medical Funds (grant 9M048 for 9N035 for TeLeht), Juho Vainio Foundation, Paavo Nurmi Foundation, Finnish Foundation of Cardiovascular Research and Finnish Cultural Foundation, Tampere Tuberculosis Foundation and Emil Aaltonen Foundation.
